# Selectivity Effects of Hydrogen Acceptors and Catalyst Structures in Alcohol Oxidations Using (Cyclopentadienone)iron Tricarbonyl Compounds

**DOI:** 10.1021/acs.joc.4c02846

**Published:** 2025-01-23

**Authors:** Melanie Hempel, Auden Cameron Lampariello, Nicolle Elahian López, Cole Springer, Kimberly McCaskey, Sneha Jayaram, Kathryn M. J. Wnuk-Fink, Bryn K. Werley, Timothy W. Funk

**Affiliations:** † Department of Chemistry, Gettysburg College, Gettysburg, Pennsylvania 17325, United States; ‡ Department of Biochemistry, Virginia Tech, Blacksburg, Virginia 24061, United States; § Department of Chemistry and Biochemistry, University of California San Diego, San Diego, California 92093, United States; ∥ Department of Chemistry, University of North Carolina, Chapel Hill, North Carolina 27599, United States

## Abstract

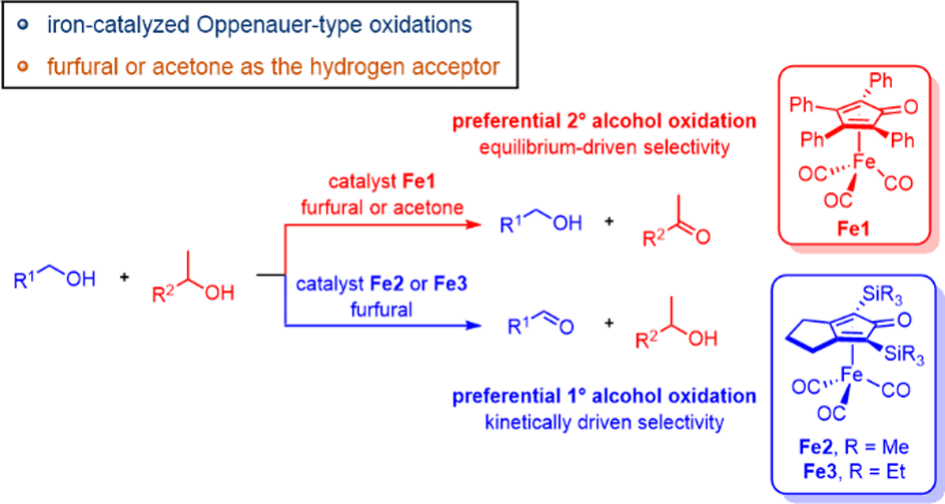

Oppenauer-type oxidations are catalyzed by air- and moisture-stable, sustainable, (cyclopentadienone)iron carbonyl compounds, but the substrate scope is limited due to the low reduction potential of acetone, which is the most commonly used hydrogen acceptor. We discovered that furfural, an aldehyde derived from cellulosic biomass, is an effective hydrogen acceptor with this class of catalysts. In general, reactions using furfural as the hydrogen acceptor led to higher isolated yields of ketones and aldehydes compared to those using acetone. Importantly, primary benzylic and allylic alcohols—typically a challenging class of alcohols to oxidize with these catalysts—could be oxidized. The selectivity for primary vs secondary alcohol oxidation with (cyclopentadienone)iron carbonyl catalysts was also explored using acetone and furfural as the hydrogen acceptors. Most of the catalysts tested preferentially oxidized unhindered secondary alcohols, but catalysts with trialkylsilyl groups in the 2- and 5-positions of the cyclopentadienone preferentially oxidized primary alcohols. A combination of substrate scope experiments and kinetic studies concluded that the selectivity with the trialkylsilyl-based catalysts was kinetically derived—primary alcohols were oxidized more quickly than secondary—and the selectivity for secondary alcohol oxidation with the other catalysts arose from the equilibrium-driven nature of the Oppenauer-type oxidation.

## Introduction

Alcohol oxidations are fundamental transformations that find widespread use and are increasingly important as chemical feedstocks transition from olefin-rich petrochemicals to oxygen-rich, biomass-derived compounds.^1−4^ There are many methods used to oxidize alcohols, including stoichiometric oxidants such as Cr(VI) species,^5−7^ activated sulfoxides,^8−10^ and hypervalent iodine^11,12^; catalytic processes^13,14^ using metal, organic, or enzymatic catalysts with NaOCl,^15,16^ hydrogen peroxide,^17−23^ or molecular oxygen^22,24−29^ as the terminal oxidant; and acceptorless dehydrogenations,^30−33^ which typically use a metal catalyst and no terminal oxidant and release molecular hydrogen as the only byproduct. Unfortunately, there are safety and sustainability concerns when performing oxidation reactions with strong oxidants or producing flammable gases, especially on large scales.^34−37^

**Figure 1:**
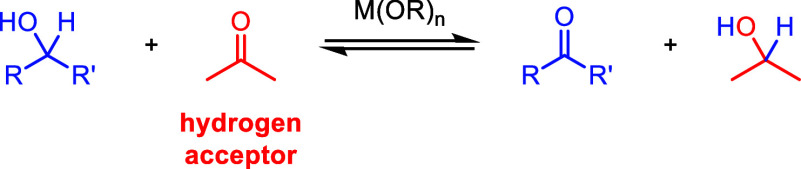
General transfer dehydrogenation (Oppenauer oxidation) reaction using acetone as the hydrogen acceptor.

Transfer dehydrogenations of alcohols to ketones and aldehydes, colloquially known as Oppenauer oxidations, are alternative, equilibrium-driven processes that do not use traditional strong oxidants or produce flammable dihydrogen.^38−41^ Instead, they use a Lewis acid catalyst—typically an aluminum or alkali metal alkoxide—and acetone or another simple carbonyl compound as the hydrogen acceptor/terminal oxidant (Figure 1). The highly Lewis acidic aluminum catalysts often must be added in stoichiometric amounts due to substrate and water deactivation and can cause side reactions (e.g., aldol condensation, Tishchenko reaction).^39,40^ Alkali metal alkoxides can lead to undesirable reactions with base-sensitive substrates. Alternative catalysts have been developed to address these problems, including those based on Ru,^42−45^ Ir,^46−50^ Rh,^43,49,50^ Zr,^51^ B,^52^ In,^53^ and Sm.^54^

**Scheme 1:**
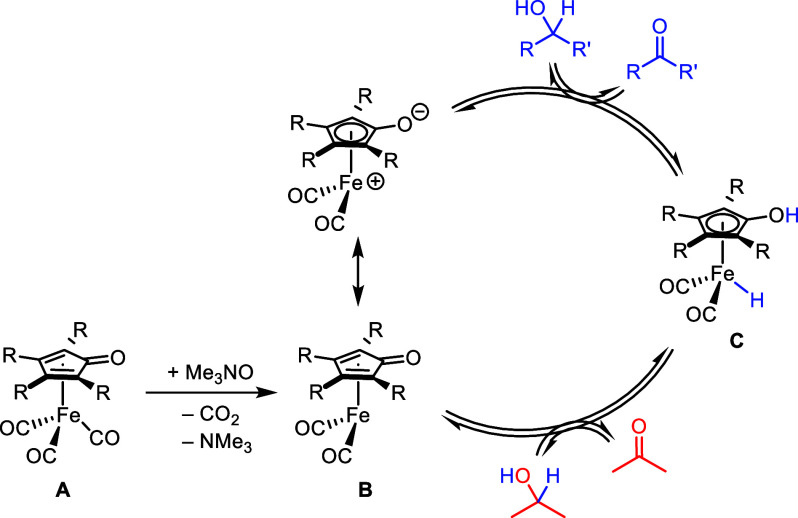
Simplified General Mechanism for (Cyclopentadienone)iron-Catalyzed Oppenauer Oxidations

Oppenauer-type oxidation catalysts based on Fe are attractive because of iron’s high natural abundance and low cost, and air-stable (cyclopentadienone)iron carbonyl catalysts are active in these reactions.^55−64^ A simplified version of the generally accepted mechanism is shown in Scheme 1.^65,66^ The tricarbonyl compound **A** is activated with trimethylamine *N*-oxide, leading to the unsaturated, active species **B**. Hydrogen is transferred from the alcohol to the catalyst, forming the ketone and iron hydride **C**. Finally, active species **B** is regenerated when hydrogen is delivered from hydride **C** to the hydrogen acceptor.

The equilibrium position of Oppenauer-type oxidations is determined by the reduction potentials of the carbonyl compounds: the equilibrium favors the formation of the alcohol of the carbonyl compound with the higher reduction potential. Therefore, the oxidation is favored for alcohols whose corresponding ketones or aldehydes have reduction potentials lower than that of the hydrogen acceptor (i.e., stronger hydrogen acceptors have higher reduction potentials).^39,67,68^ Acetone has been used as both the solvent and hydrogen acceptor in a majority of applications with (cyclopentadienone)iron carbonyl catalysts, but the scope of alcohols that can be oxidized are limited by its relatively low reduction potential (129 mV).^67^ Ketones derived from electron-poor secondary alcohols and most aldehydes are challenging to form via Oppenauer oxidation as their reduction potentials are higher than that of acetone, resulting in an unfavorable equilibrium. There are a few examples using hydrogen acceptors other than acetone with (cyclopentadienone)iron carbonyl catalysts. Bäckvall and co-workers developed an aerobic alcohol oxidation system mimicking the electron transport chain by coupling a (cyclopentadienone)iron carbonyl catalyst with catalytic amounts of a substituted 1,4-benzoquinone and a cobalt compound, but molecular oxygen was the terminal oxidant, leading to potential safety issues.^60^ Wills and co-workers showed that the use of simple aliphatic aldehydes as hydrogen acceptors surprisingly led to lower yields in the oxidation of primary and secondary benzylic alcohols compared to acetone. Interestingly, little to no ester formation (through a Tishchenko-type reaction) was observed.^57^ When they used paraformaldehyde as the hydrogen acceptor, yields increased but mixtures of ketone/aldehyde and formate esters formed.

Here we describe a (cyclopentadienone)iron-catalyzed Oppenauer-type oxidation using nontraditional hydrogen acceptors, including furfural. With use of a low-cost, earth-abundant iron catalyst and biomass-derived hydrogen acceptor, this work demonstrates a viable green alternative to traditional alcohol oxidations that is consistent with the global transition toward sustainable chemical practices Additionally, the equilibrium-driven nature of Oppenauer-type oxidations typically leads to secondary alcohols being selectively oxidized over primary alcohols, and we probed how hydrogen acceptor and catalyst structure affected primary vs secondary alcohol selectivity with this class of iron catalysts.

## Results and Discussion

### Reaction Optimization

There are a few examples of Oppenauer-type catalyst/hydrogen-acceptor systems that have been used to oxidize primary alcohols, including *t*-BuOSmI_2_/furfural or pivaldehyde,^54^ Al(O*i*Pr)_3_/furfural,^69^ (C_6_H_5_)_2_B(OH)/pivaldehyde,^52^ and AlMe_3_/3-nitrobenzaldehyde or 2,4-dinitrobenzaldehyde.^70^ These examples use aldehydes as hydrogen acceptors to take advantage of their higher reduction potentials,^67^ and limited side products were observed. Inspired by these reports, we tested the aldehydes and electron-poor ketones shown in Table 1 as hydrogen acceptors in the oxidation of 4-phenyl-2-butanol. Tetraphenylcyclopentadienone compound **1** was selected as the catalyst because of its low cost, simple preparation, and high activity in transfer dehydrogenations.^57,61^ 3-Nitrobenzaldehyde led to higher conversions at lower temperatures (Table 1, entries 1–3), and the opposite trend was observed with ethyl acetoacetate (Table 1, entries 4–6). Nitroarenes are reduced via transfer hydrogenation with alcohols using this class of catalysts at temperatures as low as 80 °C, which could have caused side reactions that hindered catalyst turnover when 3-nitrobenzaldehyde was used.^71−73^ The highest conversions were obtained when furfural was used as the hydrogen acceptor at 80 °C (Table 1, entry 8). We were able to decrease the amount of furfural to two equivalents without decreasing the conversion (Table 1, entries 8 and 10), but the conversion suffered when one equivalent was used (Table 1, entry 11). Lowering the catalyst loading to 1 mol % also decreased the conversion (Table 1, entry 12).

**Table 1:**
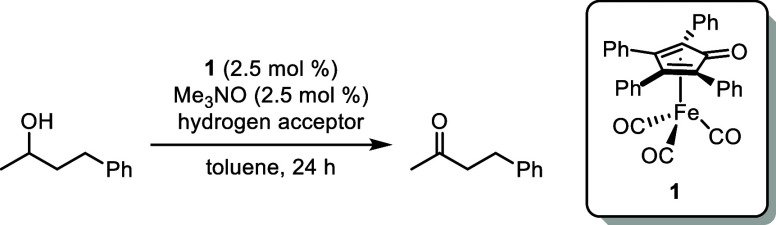
Hydrogen Acceptor Screening in the Oppenauer Oxidation of 4-Phenyl-2-butanol Using **1**

entry	H_2_ acceptor (eq^a^)	*T* (°C)	conversion^b^ (%)
1	3-nitrobenzaldehyde (5)	60	87
2	3-nitrobenzaldehyde (5)	80	76
3	3-nitrobenzaldehyde (5)	100	56
4	ethyl acetoacetate (5)	60	35
5	ethyl acetoacetate (5)	80	79
6	ethyl acetoacetate (5)	100	85
7	furfural (5)	60	82
8	furfural (5)	80	98
9	furfural (5)	100	76
10	furfural (2)	80	98 (93)^c^
11	furfural (1)	80	75
12^d^	furfural (2)	80	90

a Hydrogen acceptor equivalents relative to 4-phenyl-2-butanol.

b Determined by GC using biphenyl as an internal standard.

c Isolated yield.

d 1 mol % of catalyst **1** used.

There is a sustainability benefit from using furfural as the hydrogen acceptor: it is derived from cellulosic biomass.^74^ In an attempt to further decrease the reaction’s environmental impact, toluene was replaced with solvents deemed as safer and more sustainable with respect to green chemistry principles,^75,76^ but toluene afforded the highest conversions of the solvents examined (Supporting Information, Table S1). Interestingly, the conversion of 4-phenyl-2-butanol was only 57% when furfural was used as the solvent.

As mentioned earlier, traditional Oppenauer oxidations are often inefficient in the oxidation of primary alcohols. To further explore the scope of furfural as a hydrogen acceptor, reactions with cinnamyl alcohol were performed using the optimized conditions. The oxidation of cinnamyl alcohol using furfural as the hydrogen acceptor is favored based on their relative reduction potentials: 186 mV for cinnamaldehyde and 214 mV for furfural.^67^ As shown in Table 2, the conversion to cinnamaldehyde was high and increased as furfural equivalents increased, with five equivalents being the minimum amount producing conversions >90%.

**Table 2:**
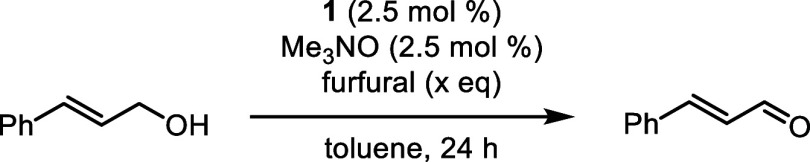
Screening of Hydrogen Acceptor Equivalents in the Oppenauer Oxidation of Cinnamyl Alcohol

entry	furfural eq^a^	conversion^b^ (%)
1	2	75
2	3	81
3	4	83
4	5	98

a Furfural equivalents relative to cinnamyl alcohol.

b Determined by GC using biphenyl as an internal standard.

### Reaction Details and Substrate Scope

**Figure 2:**
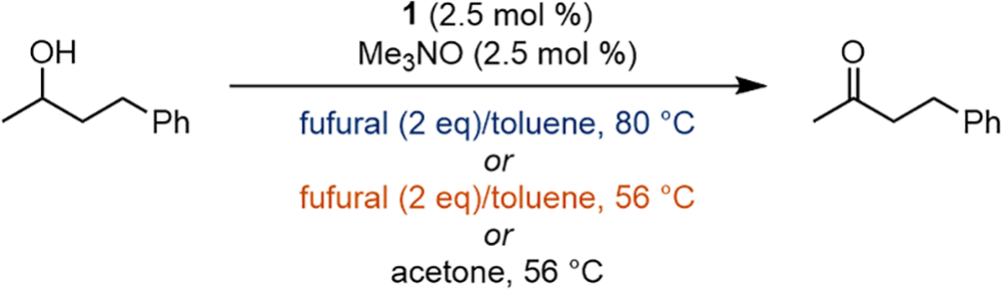
Conversion vs time for the Oppenauer oxidation of 4-phenyl-2-butanol using furfural at 56 and 80 °C and acetone at 56 °C. Conversions were determined by GC using biphenyl as the internal standard.

The optimal reaction temperature using furfural was 80 °C compared to 56 °C when acetone at reflux is used as both the solvent and hydrogen acceptor. We monitored the oxidation of 4-phenyl-2-butanol over 24 h to determine how the reaction temperature affected the reaction rate (Figure 2). Not surprisingly, the reaction with furfural in toluene at 80 °C is faster than the reaction in acetone at reflux. After 6 h, conversions were >90% when furfural at 80 °C was used but were 30% in the reaction in acetone at reflux. We hypothesized that the rate difference was due to the temperature difference, which was tested by performing the oxidation with furfural at 56 °C (reflux temperature of acetone) for a more direct comparison. Interestingly, the initial rate of the furfural/toluene reaction was faster than the reaction in acetone, but their rates were similar after 30 min. These data are consistent with the large rate difference being primarily due to the higher furfural reaction temperature.

We used the optimized conditions to explore the scope of secondary and primary alcohols we could oxidize (Table 3). For many alcohols, we compared the yields with furfural at 80 °C to the same reactions run in acetone at both reflux and 80 °C (in a sealed vessel). To make reasonable comparisons, all reactions were run for 24 h.

**Table 3:**
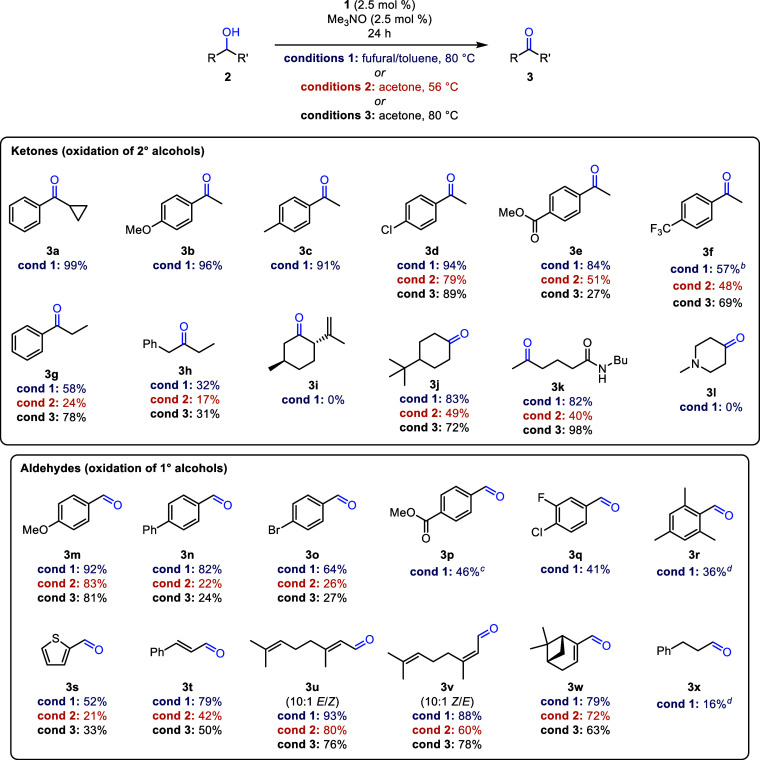
Oxidations of Primary and Secondary Alcohols Using Furfural as the Hydrogen Acceptor^a^

a Isolated yields. Conditions 1: alcohol (1 equiv), furfural (2 equiv with 2° alcohols or 5 equiv with 1° alcohols), **1** (2.5 mol %), and trimethylamine *N*-oxide (2.5 mol %) in toluene (0.5 M in alcohol) at 80 °C for 24 h; conditions 2: alcohol (1 equiv), **1** (2.5 mol %), and trimethylamine *N*-oxide (2.5 mol %) in acetone (0.5 M in alcohol) at 56 °C for 24 h; conditions 3: alcohol (1 equiv), **1** (2.5 mol %), and trimethylamine *N*-oxide (2.5 mol %) in acetone (0.5 M in alcohol) at 80 °C in a sealed vessel for 24 h.

b Conversion was 95% by GC; aldol condensation product was observed by ^1^H NMR spectroscopy.

c Isolated as a 1:3 mixture of **3p** to furfural.

d Conversion determined by ^1^H NMR spectroscopy.

**Figure 3:**
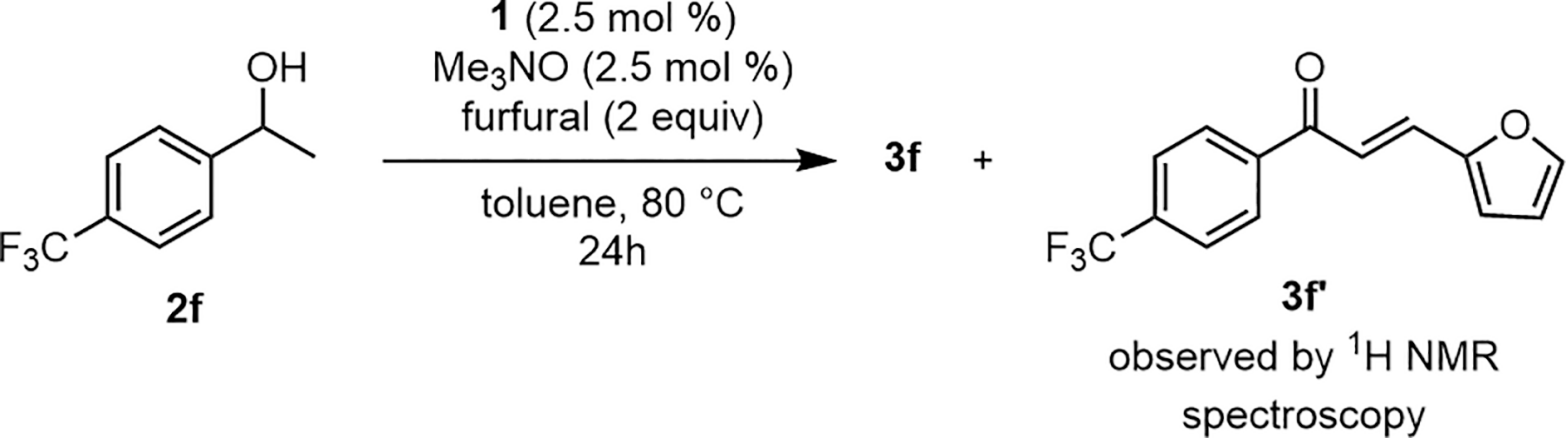
Formation of **3f′** from an aldol condensation of electron-poor ketone **3f** and furfural.

The oxidation of secondary benzylic alcohols worked well with furfural (**3a**–**3e**), and an electron-poor secondary alcohol was oxidized more effectively with furfural compared to acetone (**3e**). When the benzylic alcohol became increasingly electron-poor (**2f**), the oxidation was still successful but some of the resulting ketone (**3f**) underwent an aldol condensation with furfural to afford the α,β-unsaturated ketone **3f′** (Figure 3). The presence of the byproduct was confirmed by peaks corresponding to the furan ring and by doublets at 7.42 and 7.62 ppm—the vinyl hydrogen atoms—in the ^1^H NMR spectrum of the crude product.^77^ No aldol products were observed in the reactions with acetone. An unhindered cyclohexanol was oxidized to the corresponding cyclohexanone (**3j**), but a hindered cyclohexanol was completely unreactive (**3i**). Similarly, replacing a methyl group with an ethyl on the alcohol carbon decreased oxidation yields (**3g** and **3h**), which implies the oxidation of secondary alcohols is sensitive to mild increases in steric hindrance and is not driven exclusively by thermodynamics. Yamamoto and co-workers observed a similar trend in Oppenauer oxidations using a diarylborinic acid catalyst.^52^ An alcohol with an amide was oxidized using both furfural and acetone at 80 °C (**3k**), but an amine was not tolerated (**3l**). Other alcohols with basic nitrogen atoms, including pyridines and primary amines, were also unreactive. These results were unsurprising because amines are known to bind to the empty coordination site on iron and hinder catalysis.^66,78^ Three ketones (**3f**, **3g**, and **3k**) were isolated with yields 12–20% higher in acetone at 80 °C (conditions 3) compared to furfural as the hydrogen acceptor (conditions 1). However, reactions performed in acetone at 80 °C are potentially dangerous due to elevated pressures.

Primary benzylic and allylic alcohols were oxidized more effectively using furfural as the hydrogen acceptor compared to acetone (Table 3), presumably due to furfural’s higher reduction potential.^67^ Aldehyde yields decreased as the alcohols became more electron poor (**3m**–**3q**), which is consistent with increasing aldehyde reduction potentials. As was observed with the secondary alcohols, steric hindrance led to lower conversions (**3r**), which illustrates kinetic barriers to alcohol oxidation can override favorable thermodynamics when using catalyst **1**. A thiophene core was tolerated (**3s**), and alkene stereochemistry of allylic alcohols was not eroded during the oxidations: geraniol afforded geranial (**3u**) and nerol afforded neral (**3v**). Aliphatic primary alcohols were not oxidized effectively (**3x**), which is consistent with the reduction potentials of aliphatic aldehydes being higher than that of furfural.^67^ Quintard and co-workers observed similar trends when they studied the alcohol oxidation/carbonyl reduction component of a borrowing hydrogen process using crotonaldehyde and an aliphatic alcohol or crotyl alcohol and an aliphatic aldehyde.^79,80^

**Figure 4:**
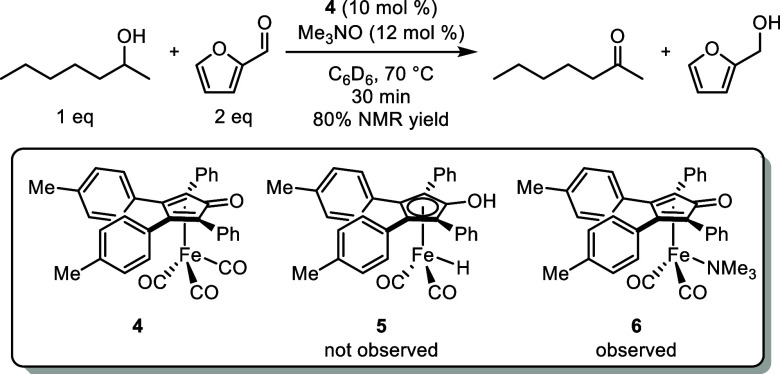
Oppenauer oxidation of 2-heptanol by **4** using furfural as the hydrogen acceptor monitored by ^1^H NMR spectroscopy.

While the reactions with furfural often led to higher conversions, furfuryl alcohol—a reaction byproduct (Figure 4)—and unreacted furfural introduced challenges to product isolation. Furfural and furfuryl alcohol are both soluble in water and could be removed by aqueous extractions. Unfortunately, many of the lower molecular-weight aldehydes and ketones in Table 3 were partially soluble in the aqueous layer and were removed by the extractions, decreasing the isolated yields relative to the conversions determined by GC and NMR spectroscopy. Therefore, a typical workup involved solvent evaporation followed by flash chromatography; however, chromatographic separation was challenging because many of the products in Table 3 eluted close to unreacted furfural. These challenges were substrate-dependent, but they were a disadvantage relative to the reactions run in acetone, where the solvent/hydrogen acceptor and byproduct (isopropanol) are volatile and easily removed by evaporation.

To gain insight into the species present in solution, we monitored the iron-catalyzed oxidation of 2-heptanol at 70 °C by ^1^H NMR spectroscopy using furfural as the hydrogen acceptor (Figure 4). We used the tolyl derivative of the catalyst (**4**) due to its favorable solubility in C_6_D_6_. Spectra were taken every 10 min over 30 min. As the reaction progressed, furfural was consumed and furfuryl alcohol formed in approximately a 1:1 ratio with 2-heptanone, which is consistent with a transfer dehydrogenation mechanism. No iron hydride (**5**) or bridging hydrides were observed in the spectra down to–30 ppm.^55^ A new signal at 8.3 ppm appeared when **4** was treated with trimethylamine *N*-oxide. We attributed this signal to trimethylamine-bound **6**, which is known to form in solution when transfer dehydrogenations are done in acetone and when this class of compounds is treated with trimethylamine *N*-oxide.^66,81^

### Selective Alcohol Oxidations: Primary vs Secondary

Oppenauer-type oxidations typically favor the oxidation of secondary over primary alcohols due to their equilibrium-driven nature.^39,40,82^ Additionally, we discovered in a previous study that **1** favored the oxidation of the secondary alcohol of a diol containing both primary and secondary alcohols when acetone was used as the hydrogen acceptor.^63^ This selectivity (2°/1°) was explored in more detail, comparing furfural (conditions A) and acetone (conditions B) as the hydrogen acceptors, and the results are shown in Table 4. Overall, there was not a large difference between the reactions using furfural and acetone (conditions A and B, respectively); both sets of conditions favored secondary alcohol oxidation over primary. When furfural was used as the hydrogen acceptor (conditions A: Table 4, entries 1, 3, and 5), the conversions of both the primary and secondary alcohols were 3–16% lower compared to acetone (conditions B: Table 4, entries 2, 4, and 6). However, the secondary-to-primary alcohol selectivity increased using conditions A. Only one equivalent of furfural was used because it can oxidize both secondary and benzylic primary alcohols. The decreased amount of furfural likely caused the lower conversions with conditions A. The secondary-to-primary alcohol selectivity more than tripled (with furfural) or doubled (with acetone) when the primary alcohol was an aliphatic alcohol (Table 4, entries 5 and 6). This high selectivity is likely due to the high reduction potentials of aliphatic aldehydes compared to benzylic and allylic aldehydes.^67^

**Table 4:**
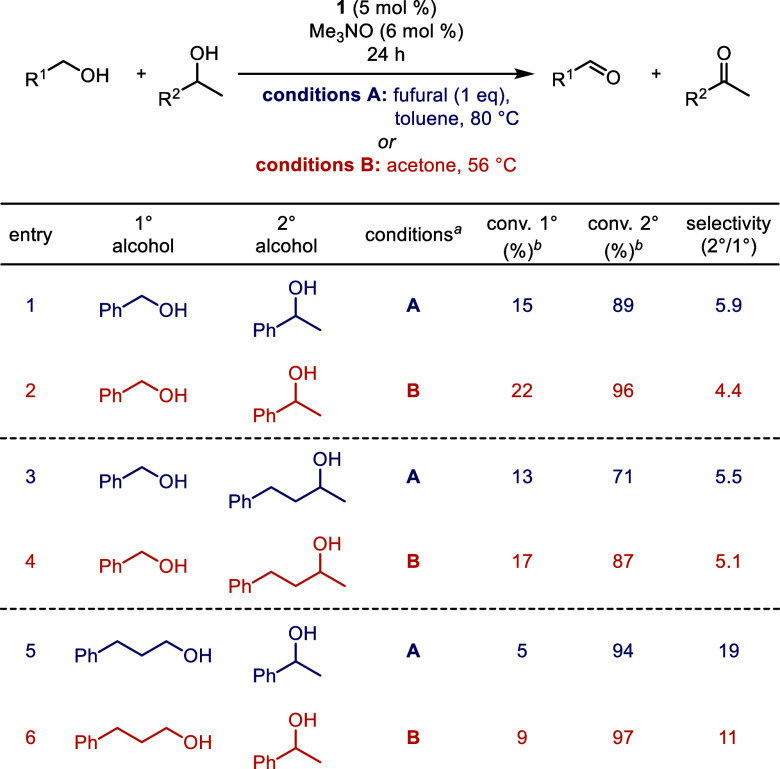
Selective Oxidations of Secondary vs Primary Alcohols with Catalyst **1**

a Conditions A: 1° alcohol (1 equiv), 2° alcohol (1 equiv), biphenyl (0.25 equiv, internal standard), furfural (1 equiv), **1** (5 mol %), and trimethylamine *N*-oxide (6 mol %) in toluene (0.5 M in 1° alcohol) at 80 °C for 24 h; conditions B: 1° alcohol (1 equiv), 2° alcohol (1 equiv), biphenyl (0.25 equiv, internal standard), **1** (5 mol %), and trimethylamine *N*-oxide (6 mol %) in acetone (0.5 M in 1° alcohol) at reflux for 24 h.

b Conversions determined by GC relative to biphenyl. A small excess of furfural likely caused the total conversion to be slightly above 100% in some reactions with conditions A.

The above experiments illustrated that hydrogen acceptors and alcohol structures affected oxidation selectivity. Modifications to the cyclopentadienone ring are known to affect the reactivity of this class of iron catalysts,^66^ so we explored the oxidation activity and secondary-to-primary alcohol selectivity of other (cyclopentadienone)iron carbonyl catalysts using furfural as they hydrogen acceptor (Table 5). Catalysts with aryl rings in the 3- and 4-positions of the cyclopentadienone selectively oxidized the secondary alcohol over the primary alcohol (Table 5, catalysts **7**–**10**). The selectivity switched when the trimethylsilyl (TMS) catalysts **11** and **12** were used, and the reversal in selectivity was not due to the introduction of the fused ring (Table 5, catalyst **13**). During our previous exploration of the lactonization of diols using this class of catalysts, we discovered that iron catalysts with TMS groups in the 2- and 5-positions of the cyclopentadienone ring reacted selectively with the primary alcohol of diols bearing both primary and secondary alcohols,^63^ and the current results are consistent with that discovery.

**Table 5:**
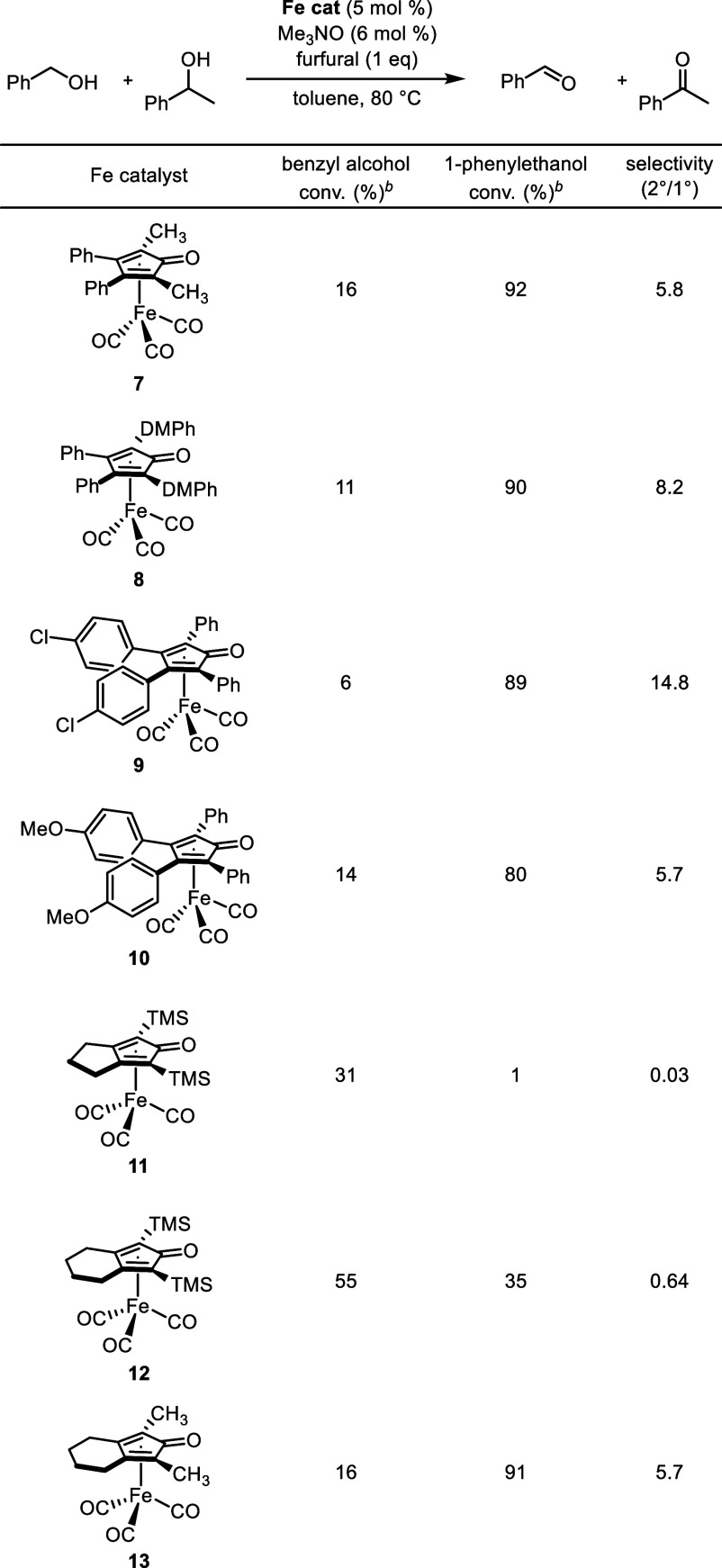
Selective Oxidation of Benzyl Alcohol and 1-Phenylethanol with (Cyclopentadienone)iron Carbonyl Catalysts^a^

a Reaction conditions: benzyl alcohol (1 equiv), 1-phenylethanol (1 equiv), biphenyl (0.25 equiv, internal standard), furfural (1 equiv), iron catalyst (5 mol %), and trimethylamine N-oxide (6 mol %) in toluene (0.5 M in benzyl alcohol) at 80 °C for 24 h.

b Conversions determined by GC relative to biphenyl. A small excess of furfural likely caused the total conversion to be slightly above 100% in some reactions. DMPh = 3,5-dimethylphenyl; TMS = trimethylsilyl.

### Synthesis and Comparative Reactivity of Sterically Bulky Catalysts

**Figure 5:**
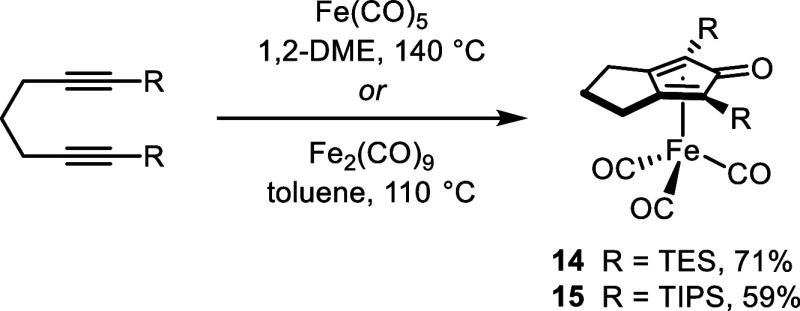
Synthesis of catalysts **14** and **15**. TES = triethylsilyl; TIPS = triisopropylsilyl.

The selectivity reversal with the TMS catalysts suggested their steric bulk may be dictating whether they react preferentially with a primary or a secondary alcohol (i.e., whether kinetic factors were overriding the thermodynamically favored product distribution). To test this hypothesis, we synthesized catalysts **14** and **15**,^83^ which contained triethylsilyl (TES) and triisopropylsilyl (TIPS) groups, respectively (Figure 5). A fused 5-membered ring in the 3- and 4-positions was used due to the high primary-to-secondary selectivity with catalyst **11**.

The activities and selectivities (1°/2°) of **11**, **14**, and **15** were tested in a series of primary vs secondary alcohol oxidation reactions and compared to **1** (Table 6). An increased amount of trimethylamine *N*-oxide (3 equiv relative to the iron catalyst) was required to obtain higher conversions when catalysts **11**, **14**, and **15** were used, which could be due to the bulky trialkylsilyl groups in the 2- and 5-positions hindering the activating agent. All of the catalysts bearing silyl groups on the cyclopentadienone preferentially oxidized the primary alcohol, as opposed to secondary alcohol oxidization being favored for **1**. The primary-to-secondary alcohol oxidation selectivity increased as the silyl groups became bulkier (Table 6, catalysts **11**, **14**, and **15**), which is consistent with the hypothesis that steric interactions are driving the selectivity. Importantly, these selectivities occurred even when five equivalents of furfural were used to push the reactions to higher conversions.

**Table 6:**
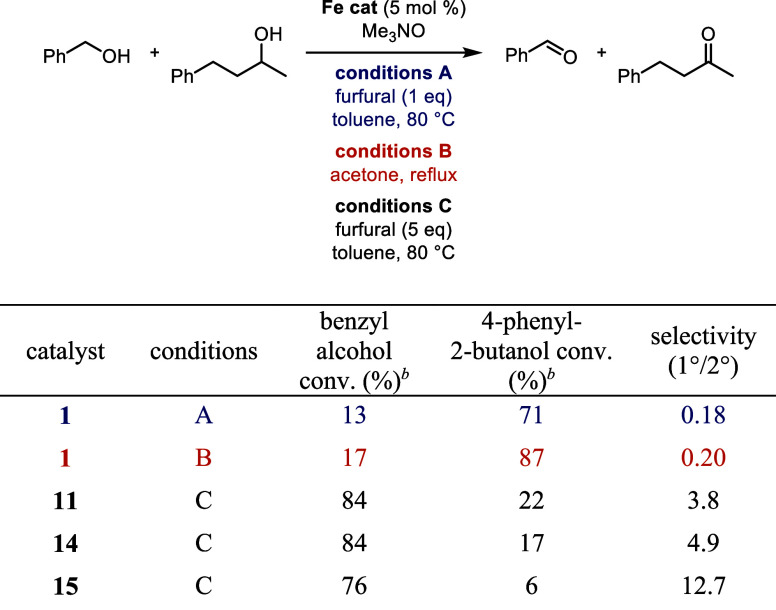
Selectivity in the Oxidation of Benzyl Alcohol and 4-Phenyl-2-butanol with (Cyclopentadienone)iron Carbonyl Catalysts^a^

a Conditions A: benzyl alcohol (1 equiv), 4-phenyl-2-butanol (1 equiv), biphenyl (0.25 equiv, internal standard), furfural (1 equiv), iron catalyst (5 mol %), and trimethylamine *N*-oxide (6 mol %) in toluene (0.5 M in benzyl alcohol) at 80 °C for 24 h; conditions B: benzyl alcohol (1 equiv), 4-phenyl-2-butanol (1 equiv), biphenyl (0.25 equiv, internal standard), iron catalyst (5 mol %), and trimethylamine *N*-oxide (6 mol %) in acetone (0.5 M in benzyl alcohol) at reflux for 24 h; conditions C: benzyl alcohol (1 equiv), 4-phenyl-2-butanol (1 equiv), biphenyl (0.25 equiv, internal standard), furfural (5 equiv), iron catalyst (5 mol %), and trimethylamine *N*-oxide (15 mol %) in toluene (0.5 M in benzyl alcohol) at 80 °C for 24 h.

b Conversions determined by GC relative to biphenyl.

Comparisons between these catalysts were made with alternative primary and secondary alcohols to examine the effect of alcohol structure and electronics on selectivity (Tables 7 and 8). In the reactions with cinnamyl alcohol and an aliphatic secondary alcohol (Table 7), there was almost no selectivity in the oxidations using **1** and only modest selectivity for the oxidation of a secondary benzylic alcohol with **1** (Table 8). These results are consistent with cinnamaldehyde having a lower reduction potential than benzaldehyde (186 mV vs 197 mV, respectively), causing cinnamyl alcohol to be easier to oxidize and decreasing the selectivity.^67^ When the silyl catalysts **11**, **14**, and **15** were used with furfural as the hydrogen acceptor, primary alcohol oxidation was favored and there was an increase in primary-to-secondary alcohol selectivity with larger silyl groups (Tables 7 and 8, catalysts **11**, **14**, and **15**, conditions C). The catalyst with the largest silyl groups (**15**) maintained high selectivity for the primary alcohol at the cost of conversion efficiency; steric hindrance between the alcohols and the bulky TIPS groups on catalyst **15** likely caused the decreased conversions. The selectivity of the silyl catalysts was also explored using acetone as the hydrogen acceptor instead of furfural, and the conversions and selectivities were 2–4 times lower (Table 7, catalysts **11**, **14**, and **15**, conditions B). These results are consistent with acetone’s lower reduction potential compared to furfural and cinnamaldehyde; the primary alcohol oxidations are less thermodynamically favored.^67^

**Table 7:**
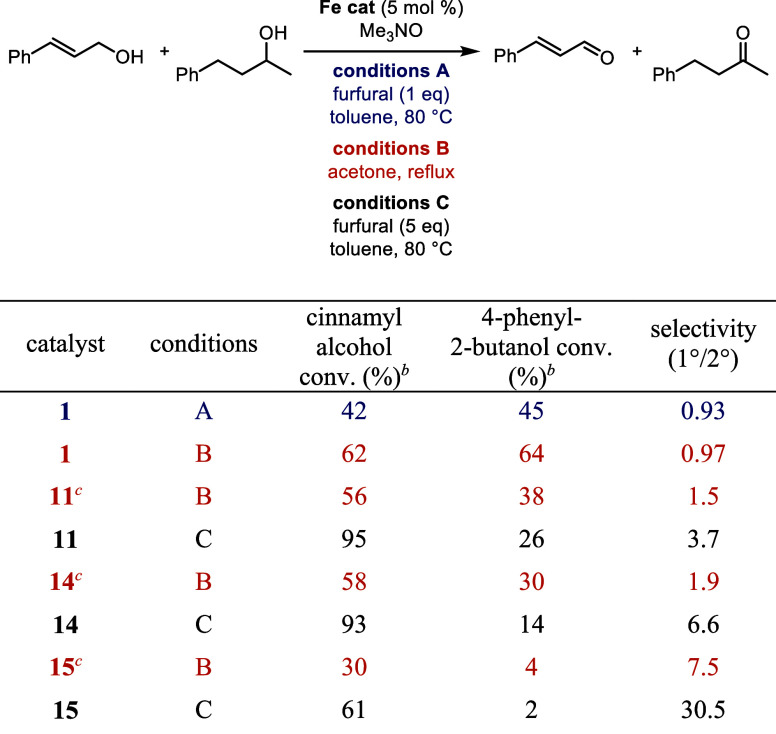
Selectivity in the Oxidation of Cinnamyl Alcohol and 4-Phenyl-2-butanol with (Cyclopentadienone)iron Carbonyl Catalysts^a^

a Conditions A: benzyl alcohol (1 equiv), 4-phenyl-2-butanol (1 equiv), biphenyl (0.25 equiv, internal standard), furfural (1 equiv), iron catalyst (5 mol %), and trimethylamine *N*-oxide (6 mol %) in toluene (0.5 M in benzyl alcohol) at 80 °C for 24 h; conditions B: benzyl alcohol (1 equiv), 4-phenyl-2-butanol (1 equiv), biphenyl (0.25 equiv, internal standard), iron catalyst (5 mol %), and trimethylamine *N*-oxide (6 mol %) in acetone (0.5 M in benzyl alcohol) at reflux for 24 h; conditions C: benzyl alcohol (1 equiv), 4-phenyl-2-butanol (1 equiv), biphenyl (0.25 equiv, internal standard), furfural (5 equiv), iron catalyst (5 mol %), and trimethylamine *N*-oxide (15 mol %) in toluene (0.5 M in benzyl alcohol) at 80 °C for 24 h.

b Conversions determined by GC relative to biphenyl.

c 15 mol % trimethylamine *N*-oxide used.

**Table 8:**
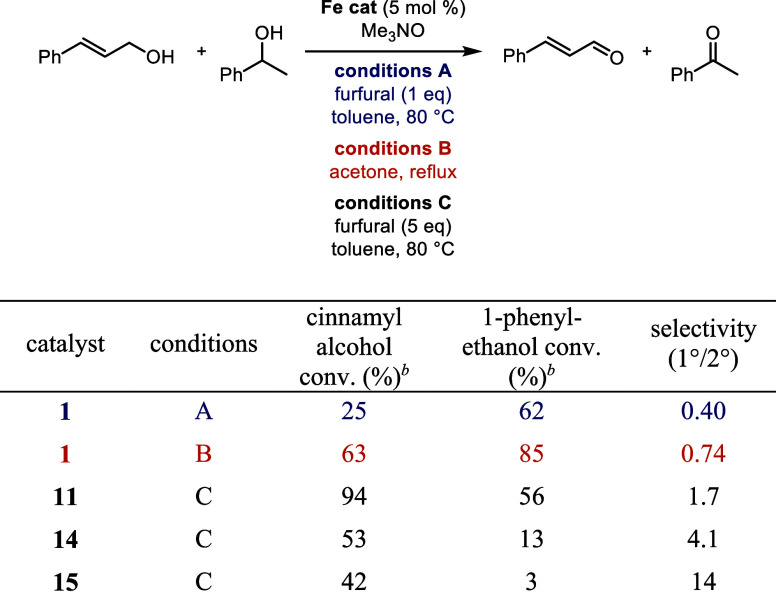
Selectivity in the Oxidation of Cinnamyl Alcohol and 1-Phenylethanol with (Cyclopentadienone)iron Carbonyl Catalysts^a^

a Conditions A: benzyl alcohol (1 equiv), 4-phenyl-2-butanol (1 equiv), biphenyl (0.25 equiv, internal standard), furfural (1 equiv), iron catalyst (5 mol %), and trimethylamine *N*-oxide (6 mol %) in toluene (0.5 M in benzyl alcohol) at 80 °C for 24 h; conditions B: benzyl alcohol (1 equiv), 4-phenyl-2-butanol (1 equiv), biphenyl (0.25 equiv, internal standard), iron catalyst (5 mol %), and trimethylamine *N*-oxide (6 mol %) in acetone (0.5 M in benzyl alcohol) at reflux for 24 h; conditions C: benzyl alcohol (1 equiv), 4-phenyl-2-butanol (1 equiv), biphenyl (0.25 equiv, internal standard), furfural (5 equiv), iron catalyst (5 mol %), and trimethylamine *N*-oxide (15 mol %) in toluene (0.5 M in benzyl alcohol) at 80 °C for 24 h.

b Conversions determined by GC relative to biphenyl.

Selectivities similar to those in the previous competition experiments between primary and secondary alcohols were observed when diol **16-Me** was oxidized (Table 9). The selectivity of catalyst **15** was not explored because it led to low conversions in the previous experiments. The oxidation of the secondary alcohol (affording **18-Me**) was high when **1** was used with either furfural (conditions A) or acetone (conditions B). When silyl catalysts **11** and **14** were used, the overall conversion was lower but primary alcohol oxidation (**17-Me)** was favored. Contrary to the previous selectivity results, catalyst **1** produced similar trends to **11** and **14** in the oxidation of a diol with a more sterically hindered secondary alcohol (**16-Ph**). All catalysts tested favored the oxidation of the primary alcohol to form **17-Ph** (Table 9), presumably because the secondary alcohol was too hindered to be efficiently oxidized by this class of catalysts, including **1**. While all catalysts demonstrated selectivity for the primary alcohol over the secondary alcohol, **11** and **14** were significantly more selective, affording no detectable amounts of the products resulting from secondary alcohol oxidations (**18-Ph** and **19-Ph**) and giving yields 20–30% higher than those obtained using catalyst **1**.

**Table 9:**
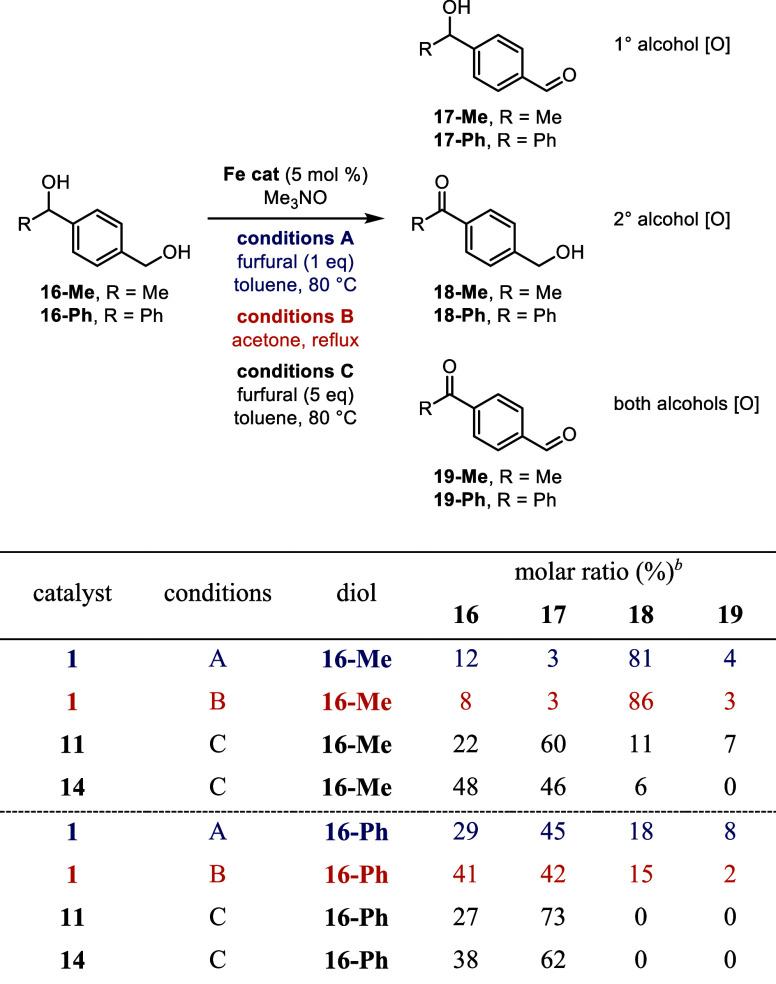
Selectivity in the Oxidation of Diols 16-Me and 16-Ph with (Cyclopentadienone)iron Carbonyl Catalysts^a^

a Conditions A: **16** (1 equiv), furfural (1 equiv), iron catalyst (5 mol %), and trimethylamine *N*-oxide (6 mol %) in toluene (0.5 M in **16**) at 80 °C for 24 h; conditions B: **16** (1 equiv), iron catalyst (5 mol %), and trimethylamine *N*-oxide (6 mol %) in acetone (0.5 M in **16**) at reflux for 24 h; conditions C: **16** (1 equiv), furfural (5 equiv), iron catalyst (5 mol %), and trimethylamine *N*-oxide (15 mol %) in toluene (0.5 M in **16**) at 80 °C for 24 h.

b Molar ratios determined by ^1^H NMR spectroscopy.

Our competition experiments showed that **1** generally favors the oxidation of secondary alcohols as long as they are not too hindered, and silyl catalysts **11**, **14**, and **15** favor the oxidation of primary benzylic and allylic alcohols over secondary alcohols. These results are consistent with our previous observations of how these catalysts behave in the lactonization of unsymmetrical diols.^63^ Additionally, Quintard and co-workers recently showed that TMS catalyst **12** selectively and reversibly oxidizes primary allylic alcohols in the presence of secondary benzylic alcohols as part of their studies on dual metal- and organocatalysis,^84^ which further supports the role of bulky silyl groups in favoring primary alcohol oxidations.

**Figure 6:**
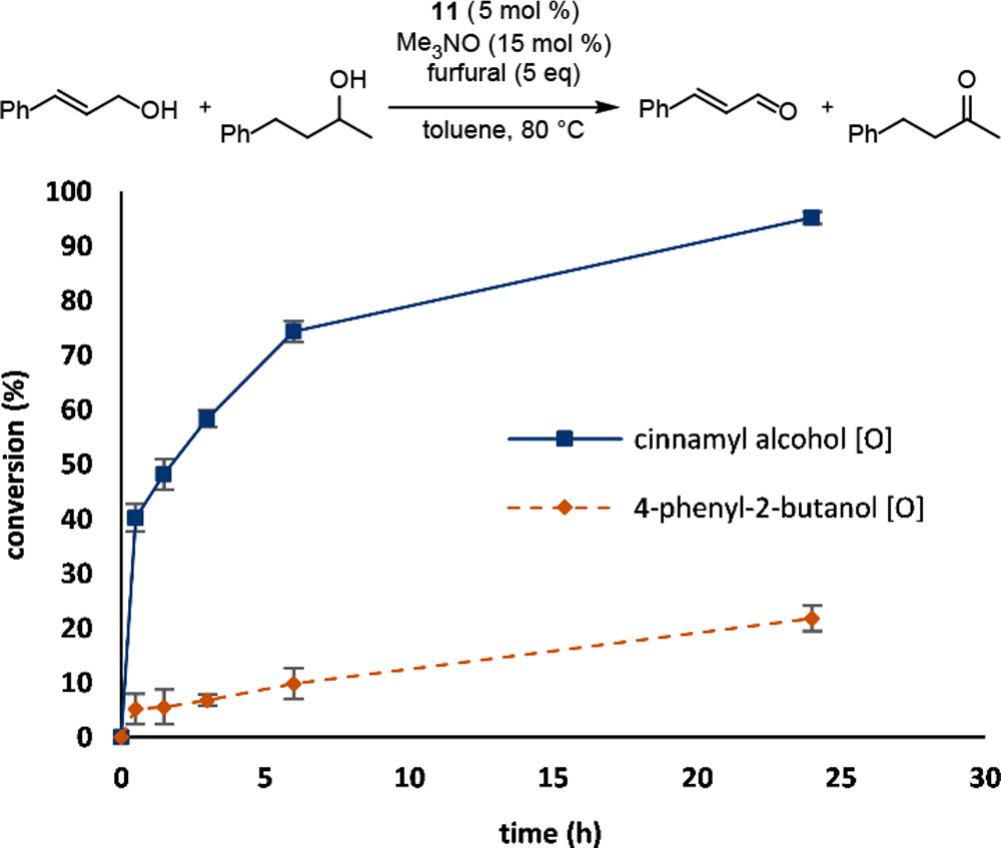
Conversion vs time for the competitive oxidation of cinnamyl alcohol and 4-phenyl-2-butanol using catalyst **11** and furfural as the hydrogen acceptor. Conversions were determined by GC using biphenyl as the internal standard.

Kinetic data was collected to probe the origin of the selectivity. A competitive oxidation reaction with cinnamyl alcohol and 4-phenyl-2-butanol using catalyst **11** was monitored over the standard 24 h reaction period (Figure 6). The primary alcohol was oxidized more quickly than the secondary alcohol, especially during the first 6 h. These results are consistent with a kinetic difference between primary and secondary alcohol oxidation when using a catalyst with a bulky silyl group.

**Figure 7:**
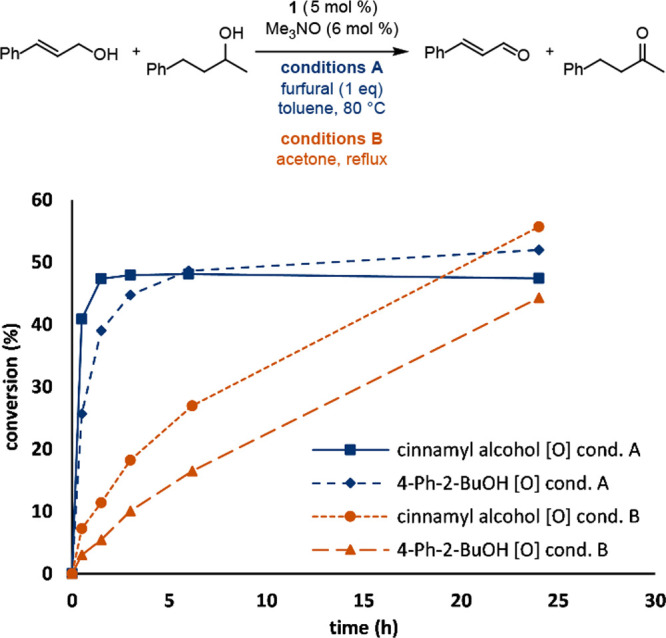
Conversion vs time for the competitive oxidation of cinnamyl alcohol and 4-phenyl-2-butanol using catalyst **1** and either furfural (blue) or acetone (orange) as the hydrogen acceptor. Conversions were determined by GC using biphenyl as the internal standard.

The same reaction was monitored using catalyst **1** with only one equivalent of furfural, and the initial rate for the oxidation of the primary alcohol was faster than the secondary alcohol but its conversion eventually surpassed that of the primary alcohol (Figure 7, conditions A). Additionally, the conversion of the primary alcohol slightly decreased after 6 h, which could be a result of cinnamaldehyde acting as a hydrogen acceptor, regenerating cinnamyl alcohol. The same reaction using acetone as the hydrogen acceptor and solvent resulted in a less severe contrast in initial oxidation rates between the primary and secondary alcohol followed by comparable oxidation rates over time (Figure 7, conditions B), but the primary alcohol was still being oxidized more quickly.

**Figure 8:**
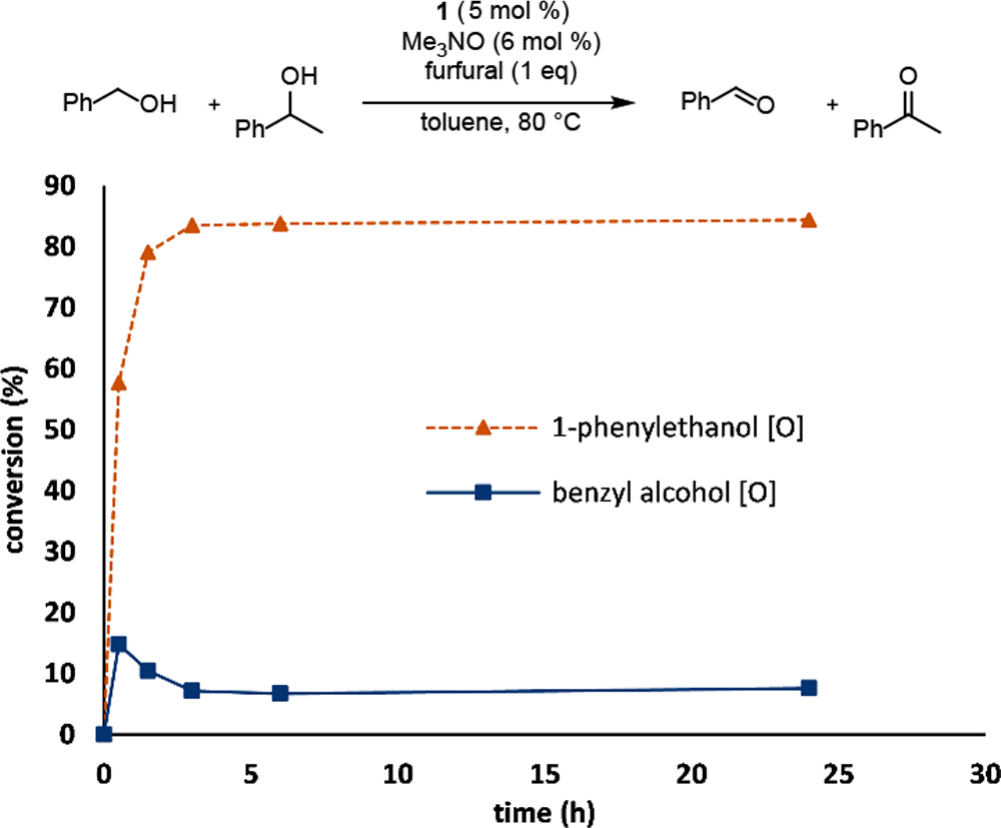
Conversion vs time for the competitive oxidation of benzyl alcohol and 1-phenylethanol using catalyst **1** and furfural as the hydrogen acceptor. Conversions were determined by GC using biphenyl as the internal standard.

The results in Figure 7 were not surprising considering the low selectivity obtained using catalyst **1** in previous competition experiments with cinnamyl alcohol as the primary alcohol (Table 7). To further characterize the kinetics of these oxidation reactions with catalyst **1**, entry 1 from Table 4—a reaction selective for secondary alcohol oxidation—was repeated with time points taken over 24 h (Figure 8). Indeed, in the reaction with 1-phenylethanol (secondary alcohol) and benzyl alcohol (primary alcohol), the secondary alcohol was rapidly oxidized within the first 30 min and conversion plateaued after just a few hours. The conversion of the primary alcohol showed an interesting trend: benzyl alcohol was initially consumed but regenerated as the reaction progressed. These results are consistent with benzyl alcohol initially being rapidly oxidized to benzaldehyde, followed by both furfural and benzaldehyde serving as hydrogen acceptors in the oxidation of 1-phenylethanol. They are also consistent with the slight decrease in conversion for cinnamyl alcohol observed in Figure 7 and with acetophenone’s much lower reduction potential relative to benzaldehyde.^67^

The primary-vs-secondary competition experiments and reaction monitoring data provided insight into the origins of selectivity with this class of iron catalysts. The use of catalyst **1** with either furfural or acetone typically led to selective oxidations of secondary alcohols over primary alcohols, but **1** was less reactive with sterically bulky secondary alcohols. The selectivity did not entirely arise from secondary alcohols undergoing oxidations more rapidly than primary alcohols; instead, it was due in large part to the reversibility of the hydrogen transfer reaction. When the primary alcohol was oxidized, the corresponding aldehyde served as a hydrogen acceptor, regenerating the primary alcohol. Because **1** oxidized both primary and secondary alcohols, the selectivity only occurred when one equivalent of furfural or when acetone was used. In both cases, selectivity was highest when the aldehyde of the primary alcohol had a significantly higher reduction potential than the ketone of the secondary alcohol. These results are in contrast to an AlMe_3_-catalyzed Oppenauer oxidation reported by Nguyen and co-workers, where they observed a secondary alcohol being oxidized more quickly than a primary alcohol in the presence of their electron-poor hydrogen acceptor.^70^ They were also able to oxidize sterically hindered secondary alcohols that **1** had limited reactivity toward. Many of the typical reagents used to selectively oxidize secondary alcohols are electrophilic and react irreversibly, and therefore are driven by a kinetic selectivity for the more electron-rich alcohol.^82^

Sterically bulky catalysts (**11**, **14**, **15**) were even more sensitive to sterically hindered secondary alcohols than **1** and preferentially oxidized primary allylic and benzylic alcohols. The selectivity was kinetically driven by alcohol steric hindrance—primary alcohols were oxidized more quickly than secondary alcohols. Other metal-mediated and -catalyzed methods that are selective for primary alcohol oxidations have also been postulated to be driven by steric hindrance.^27,85,86^

## Conclusions

We discovered that furfural was an effective hydrogen acceptor in (cyclopentadienone)iron-catalyzed, Oppenauer-type oxidations of primary and secondary alcohols. The reactions proceeded through a transfer dehydrogenation mechanism, producing furfuryl alcohol as a byproduct. No iron hydrides or bridging hydrides were observed during catalysis. In general, secondary alcohols and benzylic and allylic primary alcohols were isolated in higher yields when furfural was used as a hydrogen acceptor compared to acetone; however, sterically hindered secondary alcohols had low reactivity. The reactions occurred more quickly using the furfural/toluene conditions relative to acetone, and the rate increase was primarily attributed to the higher reaction temperature.

The iron catalysts explored in this study were sensitive to the steric hindrance of the alcohols to varying degrees. The tetraphenylcyclopentadienone catalyst **1** oxidized both primary and secondary alcohols. Due to the reversible nature of the hydrogen transfer process, equilibria based on reduction potentials typically drove the selectivity for secondary alcohols over primary alcohols.

Catalysts with sterically bulky trialkylsilyl groups (**11**, **14**, **15**) reacted more slowly than **1** with secondary alcohols and preferentially oxidized primary alcohols. The results are consistent with the selectivity originating from differences in reaction rates between primary and secondary alcohols. Primary vs secondary oxidation selectivity increased as the trialkylsilyl group increased in size, but conversions suffered.

Overall, these results illustrate that hydrogen acceptors other than acetone can be used to expand the substrate scope of Oppenauer-type oxidations with (cyclopentadienone)iron carbonyl catalysts. The selectivity of the oxidations is dependent on the alcohol and catalyst steric hindrance, and hydrogen acceptor and carbonyl product reduction potentials. Steric hindrance in the 2- and 5-positions of the cyclopentadienone impacts alcohol oxidation rates to the point where selectivity flips: the thermodynamically favored ketone—typically the major product in Oppenauer-type oxidations—can become the minor product if the substituents adjacent to the carbonyl are large enough.

## Experimental Section

### General Information

All reactions were done under an atmosphere of nitrogen in a round-bottom flask unless otherwise noted. Reactions performed above room temperature were heated using an oil bath. Commercial chemicals were used as received with the exception of furfural, which was vacuum distilled from K_2_CO_3_ and stored under nitrogen. Reagent grade acetone and toluene were degassed by bubbling N_2_ through them for at least 15 min prior to use, but no attempt was made to remove residual water. Iron compounds **1**,^57,87^**4**,^55^**7**,^88^**8**,^61^**9**,^66^**10**,^89^**11** and **12**(90−92) and **15**,^83^ alcohol **2e**,^93^ and diols **16-Ph**(94) and **16-Me**(95) were prepared according to published procedures. All ^1^H and ^13^C{^1^H} NMR spectra were recorded at ambient temperature at 400 and 100 MHz, respectively, on a Bruker Avance Neo 400 MHz FT-NMR spectrometer unless otherwise noted. Chemical shifts are reported in parts per million (ppm) relative to tetramethylsilane (TMS) for spectra taken in CDCl_3_. ^1^H NMR spectra taken in benzene-*d*_6_ used the residual solvent peak (7.16 ppm) as a reference. High resolution mass spectrometry data were collected at the Johns Hopkins University Mass Spectrometry Facility. Analytical thin-layer chromatography (TLC) was performed using silica gel 60 F254 precoated plates (0.25 mm thickness) with a fluorescent indicator. Flash column chromatography was performed using silica gel 60 (230–400 mesh). Gas chromatograms were collected on an Agilent Technologies 7890A gas chromatography system with a 7693 autosampler and an FID. A GsBP-5 (5% diphenyl/95% dimethylpolysiloxane) column (17 m length x 0.20 mm ID x 0.33 μm film thickness) was used under the following method conditions: 85 °C for 2 min, ramp 20 °C/min to 195 °C, hold at 195 °C for 7.5 min. The carrier gas was hydrogen, used at a constant flow rate of 1 mL/min. A sample volume of 1 μL was added to the 250 °C injector at a 50:1 split ratio, and the FID temperature was 300 °C. Retention times (4.45 min for 4-phenyl-2-butanol, 2.35 min for benzyl alcohol, 2.64 min for 1-phenylethanol, 4.81 min for cinnamyl alcohol, and 5.36 min for biphenyl) were determined using pure samples.

#### *n*-Butyl-5-hydroxyhexanamide (**2k**)

δ-Hexalactone (2.00 g, 17.5 mmol) and *n*-butylamine (2.56 g, 35.0 mmol) were heated to 130 °C in a thick-walled tube with a PTFE screw cap. **Caution!***Elevated pressure could cause the tube to explode. Perform the reaction in a fume hood behind a blast shield.* After 20 h, the reaction was cooled to rt and the crude product was purified by flash chromatography (5% methanol/95% ethyl acetate) to afford 3.04 g (92%) of **2k** as a light-yellow oil. ^1^H NMR (400 MHz, CDCl_3_, ppm): δ 5.61 (br s, 1H), 3.80 (sextet, *J* = 6.0 Hz, 1H), 3.25 (q, *J* = 6.8 Hz, 2H), 2.21 (t, *J* = 7.6 Hz, 2H), 2.01 (br s, 1H), 1.74 (quintet, *J* = 8.0 Hz, 2H), 1.48 (quintet, *J* = 6.8 Hz, 4H), 1.34 (sextet, *J* = 7.6 Hz, 2H), 1.19 (d, *J* = 6.2 Hz, 3H), 0.93 (t, *J* = 7.2 Hz, 3H). ^13^C{^1^H} NMR (100 MHz, CDCl_3_, ppm): δ 173.6, 66.9, 39.2, 38.4, 36.2, 31.6, 23.4, 21.8, 20.0, 13.7. HRMS (EI) for C_10_H_21_NO_2_: calculated for M^+^*m*/*z* = 187.1572, found *m*/*z* = 187.1575.

#### [1,3-Dimethyl-4,5,6,7-tetrahydro-2H-inden-2-one]iron tricarbonyl (**13**)

A solution of iron pentacarbonyl (0.98 mL, 7.4 mmol) and 2,8-decadiyne^96^ (0.50 g, 3.7 mmol) in 24 mL of anhydrous 1,2-dimethoxyethane in a thick-walled round-bottom flask with a PTFE screw cap was heated to 140 °C for 20 h. **Caution!***Elevated pressure could cause the flask to explode. Perform the reaction in a fume hood behind a blast shield.* The volatiles were removed under reduced pressure and the crude product was purified by flash chromatography (95% CH_2_Cl_2_/5% MeOH). The resulting yellow solid was triturated in cold hexanes to afford 518 mg (46%) of **13** as a yellow solid. ^1^H NMR (400 MHz, CDCl_3_, ppm): δ 2.39–2.56 (m, 4H), 1.82–1.87 (m, 4H), 1.73 (s, 6H). ^13^C{^1^H} NMR (100 MHz, CDCl_3_, ppm): δ 209.4, 171.3 (br), 101.2, 78.7 (br), 22.0, 21.3, 8.5. HRMS (EI) for C_14_H_14_FeO_4_: calculated for M^+^*m*/*z* = 302.0242, found *m*/*z* = 302.0237.

#### 1,7-Bis(triethylsilyl)-1,6-heptadiyne

*n*-Butyllithium (1.6 M in THF; 15 mL, 24 mmol) was added dropwise to a solution of 1,6-heptadiyne (1.01 g, 10.9 mmol) in 30 mL of THF at–78 °C. After 15 min at–78 °C, chlorotriethylsilane (4.0 mL, 23.8 mmol) was slowly added. The reaction stirred at–78 °C for 1 h and at rt for 16 h. It was quenched with 30 mL of saturated aqueous NH_4_Cl and extracted with 2 × 25 mL of MTBE. The organic layers were combined, dried over sodium sulfate, filtered, and evaporated to a yellow oil that was purified by flash chromatography (99.5% hexanes/0.5% ethyl acetate) to afford 2.81 g (80%) of 1,7-bis(triethylsilyl)-1,6-heptadiyne as a colorless oil. ^1^H NMR (400 MHz, CDCl_3_, ppm): δ 2.37 (t, *J* = 7.2 Hz, 4H), 1.74 (quintet, *J* = 7.2 Hz, 2H), 0.98 (t, *J* = 8.0 Hz, 18H), 0.57 (q, *J* = 8.0 Hz, 12H). ^13^C{^1^H} NMR (100 MHz, CDCl_3_, ppm): δ 107.4, 82.2, 27.9, 18.9, 7.4, 4.5. HRMS (EI) for C_19_H_36_Si_2_: calculated for M^+^*m*/*z* = 320.2356, found *m*/*z* = 320.2362.

#### [2,5-Bis(triethylsilyl)-3,4-diphenylcyclopentadienone]iron tricarbonyl (**14**)

A solution of iron pentacarbonyl (0.75 mL, 5.7 mmol) and 1,7-bis(triethylsilyl)-1,6-heptadiyne (0.89 g, 2.78 mmol) in 23 mL of anhydrous 1,2-dimethoxyethane in a thick-walled round-bottom flask with a PTFE screw cap was heated to 140 °C for 20 h. **Caution!***Elevated pressure could cause the flask to explode. Perform the reaction in a fume hood behind a blast shield.* The volatiles were removed under reduced pressure and the crude product was purified by flash chromatography (95% hexanes/5% MTBE) to afford 962 mg (71%) of **14** as a yellow solid. ^1^H NMR (400 MHz, CDCl_3_, ppm): δ 2.56–2.60 (m, 4H), 2.33–2.36 (m, 1H), 1.81–1.91 (m, 1H), 1.0 (t, *J* = 8.0 Hz, 18H), 0.72–0.90 (m, 12H). ^13^C{^1^H} NMR (100 MHz, CDCl_3_, ppm): δ 208.9, 183.6, 117.9, 69.4, 27.8, 25.7, 7.5, 3.5. HRMS (EI) for C_23_H_36_FeO_4_Si_2_: calculated for M^+^*m*/*z* = 488.1502, found *m*/*z* = 488.1514.

### General Hydrogen Acceptor Screening Procedure

A solution of 4-phenyl-2-butanol (1 equiv) and biphenyl (0.25 equiv) was prepared in toluene (0.5 M in 4-phenyl-2-butanol) (Table 1). A 50 μL aliquot was removed, diluted with 1 mL of acetone, and analyzed by gas chromatography to give the *t* = 0 chromatogram. Hydrogen acceptor (see Table 1 for number of equivalents), **1** (0.025 equiv), and anhydrous trimethylamine *N*-oxide (0.025 equiv) were added and the reaction stirred at the desired temperature. After 24 h, a 200 μL sample of the reaction solution was diluted with 1 mL hexanes. Residual iron was removed from the sample by adding it to a Pasteur pipet half filled with silica gel and eluting with 4 mL 1:1 hexanes/ethyl acetate. A 1.2 mL sample of the eluted solution was analyzed by gas chromatography. Conversion was determined based on how much reactant had been consumed compared to the amount of reactant in the *t* = 0 chromatogram relative to the internal standard (biphenyl).

### Monitoring the Oxidation of 4-Phenyl-2-butanol over Time Using Furfural as the Hydrogen Acceptor

A solution of 4-phenyl-2-butanol (376 mg, 2.5 mmol) and biphenyl (96.4 mg, 0.625 mmol) was prepared in 5 mL of toluene (Figure 2). A 50 μL sample was removed, diluted with 1 mL of acetone, and analyzed by gas chromatography to give the *t* = 0 chromatogram. Furfural (480 mg, 5 mmol), catalyst **1** (32.8 mg, 0.0625 mmol), and anhydrous trimethylamine *N*-oxide (4.7 mg, 0.0625 mmol) were added and the reaction stirred at either 56 or 80 °C. Reaction samples (200 μL) were removed at the desired times and diluted with 1 mL of hexanes. Residual iron was removed from the sample by adding it to a Pasteur pipet half filled with silica gel and eluting with 4 mL 1:1 hexanes/ethyl acetate. A 1.2 mL sample of the eluted solution was analyzed by gas chromatography. Conversion was determined based on how much reactant had been consumed compared to the amount of reactant in the *t* = 0 chromatogram relative to the internal standard (biphenyl).

### Monitoring the Oxidation of 4-Phenyl-2-butanol over Time Using Acetone as the Hydrogen Acceptor

A solution of 4-phenyl-2-butanol (376 mg, 2.5 mmol) and biphenyl (96.4 mg, 0.625 mmol) was prepared in 5 mL of acetone (Figure 2). A 50 μL sample was removed, diluted with 1 mL of acetone, and analyzed by gas chromatography to give the *t* = 0 chromatogram. Catalyst **1** (32.8 mg, 0.0625 mmol) and anhydrous trimethylamine *N*-oxide (4.7 mg, 0.0625 mmol) were added and the reaction stirred at reflux. Reaction samples (200 μL) were removed at the desired times and diluted with 1 mL of hexanes. Residual iron was removed from the sample by adding it to a Pasteur pipet half filled with silica gel and eluting with 4 mL 1:1 hexanes/ethyl acetate. A 1.2 mL sample of the eluted solution was analyzed by gas chromatography. Conversion was determined based on how much reactant had been consumed compared to the amount of reactant in the *t* = 0 chromatogram relative to the internal standard (biphenyl).

### General Oxidation and Isolation Procedure Using Furfural as the Hydrogen Acceptor

A solution of alcohol (1 equiv), catalyst **1** (0.025 equiv), anhydrous trimethylamine *N*-oxide (0.025 equiv), and furfural (2 equiv for secondary alcohol or 5 equiv for primary alcohol) in toluene (0.5 M in alcohol) were stirred in a round-bottom flask with a condenser at 80 °C for 24 h (Table 3, Conditions 1). The volatiles were removed under reduced pressure and the crude product was purified by flash chromatography.

#### 4-Phenyl-2-butanone

Following the general procedure, 4-phenyl-2-butanol (458 mg, 3.05 mmol), **1** (39.7 mg, 0.0757 mmol), anhydrous trimethylamine *N*-oxide (5.7 mg, 0.0757 mmol), and furfural (590 mg, 6.2 mmol) in 6.1 mL toluene afforded 422 mg (93%) of 4-phenyl-2-butanone as a colorless oil after flash chromatography (94% cyclohexane/6% ethyl acetate) (Table 1 Entry 10).^97^^1^H NMR (400 MHz, CDCl_3_, ppm): δ 7.26–7.30 (m, 2H), 7.17–7.21 (m, 3H), 2.90 (t, *J* = 7.2 Hz, 2H), 2.76 (t, *J* = 7.6 Hz, 2H), 2.14 (s, 3H). ^13^C{^1^H} NMR (100 MHz, CDCl_3_, ppm): δ 208.0, 141.0, 128.5, 128.3, 126.1, 45.2, 30.1, 29.7.

#### Cyclopropyl Phenyl Ketone (**3a**)

Following the general procedure, α-cyclopropylbenzyl alcohol (**2a**) (226 mg, 1.53 mmol), **1** (20.8 mg, 0.0397 mmol), anhydrous trimethylamine *N*-oxide (3.0 mg, 0.040 mmol), and furfural (293 mg, 3.1 mmol) in 3.1 mL toluene afforded 221 mg (99%) of **3a** as a light-purple oil (due to coelution with tetraphenylcyclopentadienone) after flash chromatography (96% cyclohexane/4% ethyl acetate).^98^^1^H NMR (400 MHz, CDCl_3_, ppm): δ 8.02 (d, *J* = 7.2 Hz, 2H), 7.57 (t, *J* = 7.2 Hz, 1H), 7.48 (t, *J* = 7.2 Hz, 2H), 2.71–2.65 (m, 1H), 1.27–1.23 (m, 2H), 1.07–1.02 (m, 2H). ^13^C{^1^H} NMR (100 MHz, CDCl_3_, ppm): δ 200.7, 138.0, 132.7, 128.5, 128.0, 17.2, 11.7.

#### 4′-Methoxyacetophenone (**3b**)

Following the general procedure, 1-(4-methoxyphenyl)ethanol (**2b**) (347 mg, 2.28 mmol), **1** (30 mg, 0.057 mmol), anhydrous trimethylamine *N*-oxide (4.3 mg, 0.057 mmol), and furfural (440 mg, 4.58 mmol) in 4.6 mL toluene afforded 329 mg (96%) of **3b** as a light-yellow, low-melting, amorphous solid after flash chromatography (92% cyclohexane/8% ethyl acetate).^45^^1^H NMR (400 MHz, CDCl_3_, ppm): δ 7.94 (d, *J* = 9.2 Hz, 2H), 6.93 (d, *J* = 8.8 Hz, 2H), 3.87 (s, 3H), 2.55 (s, 3H). ^13^C{^1^H} NMR (100 MHz, CDCl_3_, ppm): δ 196.8, 163.5, 130.6, 130.3, 113.7, 55.5, 26.3.

#### 4′-Methylacetophenone (**3c**)

Following the general procedure, 1-(*p*-tolyl)ethanol (**2c**) (311 mg, 2.28 mmol), **1** (30 mg, 0.057 mmol), anhydrous trimethylamine *N*-oxide (4.3 mg, 0.057 mmol), and furfural (440 mg, 4.58 mmol) in 4.6 mL toluene afforded 279 mg (91%) of **3c** as a colorless oil after flash chromatography (97% cyclohexane/3% ethyl acetate).^99^^1^H NMR (400 MHz, CDCl_3_, ppm): δ 7.85 (d, *J* = 8.4 Hz, 2H), 7.24 (d, *J* = 8.0 Hz, 2H), 2.56 (s, 3H), 2.40 (s, 3H). ^13^C{^1^H} NMR (100 MHz, CDCl_3_, ppm): δ 197.8, 143.9, 134.7, 129.2, 128.4, 26.5, 21.6.

#### 4′-Chloroacetophenone (**3d**)

Following the general procedure, 1-(4-chlorophenyl)ethanol (**2d**) (357 mg, 2.28 mmol), **1** (30 mg, 0.057 mmol), anhydrous trimethylamine *N*-oxide (4.3 mg, 0.057 mmol), and furfural (440 mg, 4.58 mmol) in 4.6 mL toluene afforded 331 mg (94%) of **3d** as a colorless oil after flash chromatography (97% cyclohexane/3% ethyl acetate).^100^^1^H NMR (400 MHz, CDCl_3_, ppm): δ 7.89 (d, *J* = 8.8 Hz, 2H), 7.42 (d, *J* = 8.4 Hz, 2H), 2.58 (s, 3H). ^13^C{^1^H} NMR (100 MHz, CDCl_3_, ppm): δ 196.8, 139.5, 135.4, 129.7, 128.9, 26.5.

#### Methyl 4-Acetylbenzoate (**3e**)

Following the general procedure, methyl 4-(1-hydroxyethyl)benzoate (**2e**)^93^ (240 mg, 1.33 mmol), **1** (17.4 mg, 0.033 mmol), anhydrous trimethylamine *N*-oxide (2.5 mg, 0.033 mmol), and furfural (256 mg, 2.66 mmol) in 2.7 mL toluene afforded 199 mg (84%) of **3e** as an off-white, amorphous solid after flash chromatography (88% cyclohexane/12% ethyl acetate).^101^^1^H NMR (400 MHz, CDCl_3_, ppm): δ 8.12 (d, *J* = 8.0 Hz, 2H), 8.00 (d, *J* = 8.4 Hz, 2H), 3.95 (s, 3H), 2.64 (s, 3H). ^13^C{^1^H} NMR (100 MHz, CDCl_3_, ppm): δ 197.5, 166.2, 140.2, 133.9, 129.8, 128.2, 52.4, 26.8.

#### 4′-(Trifluoromethyl)acetophenone (**3f**)

Following the general procedure, 1-[4-(trifluoromethyl)phenyl]ethanol (**2f**) (253 mg, 1.33 mmol), **1** (17.4 mg, 0.033 mmol), anhydrous trimethylamine *N*-oxide (2.5 mg, 0.033 mmol), and furfural (256 mg, 2.66 mmol) in 2.7 mL toluene afforded 143 mg (57%) of **3f** as a colorless oil after flash chromatography (92% cyclohexane/8% methyl *t*-butyl ether).^101^^1^H NMR (400 MHz, CDCl_3_, ppm): δ 8.06 (d, *J* = 8.0 Hz, 2H), 7.73 (d, *J* = 8.0 Hz, 2H), 2.65 (s, 3H). ^13^C{^1^H} NMR (100 MHz, CDCl_3_, ppm): δ 197.0, 139.7, 134.4 (q, *J* = 32.5 Hz), 128.6, 125.7 (q, *J* = 3.8 Hz), 123.6 (q, *J* = 271.2 Hz), 26.8.

#### Propiophenone (**3g**)

Following the general procedure, 1-phenylpropanol (**2g**) (181 mg, 1.33 mmol), **1** (17.4 mg, 0.033 mmol), anhydrous trimethylamine *N*-oxide (2.5 mg, 0.033 mmol), and furfural (256 mg, 2.66 mmol) in 2.7 mL toluene afforded 103 mg (58%) of **3g** as a light-yellow oil after flash chromatography (98% cyclohexane/2% ethyl acetate).^99^^1^H NMR (400 MHz, CDCl_3_, ppm): δ 7.96 (d, *H* = 8.4 Hz, 2H), 7.54 (t, *J* = 7.6 Hz, 1H), 7.44 (t, *J* = 8.0 Hz, 2H), 2.99 (q, *J* = 7.6 Hz, 2H), 1.22 (t, *J* = 7.2 Hz, 3H). ^13^C{^1^H} NMR (100 MHz, CDCl_3_, ppm): δ 200.8, 136.9, 132.9, 128.5, 128.0, 31.8, 8.2.

#### 1-Phenyl-2-butanone (**3h**)

Following the general procedure, 1-phenyl-2-butanol (**2h**) (230 mg, 1.53 mmol), **1** (20.0 mg, 0.0381 mmol), anhydrous trimethylamine *N*-oxide (2.9 mg, 0.038 mmol), and furfural (293 g, 3.1 mmol) in 3.1 mL toluene afforded 72 mg (32%) of **3h** as a colorless oil after flash chromatography (92% cyclohexane/8% ethyl acetate).^102^^1^H NMR (400 MHz, CDCl_3_, ppm): δ 7.33 (t, *J* = 7.2 Hz, 2H), 7.26 (t, *J* = 7.2 Hz, 1H), 7.21 (d, *J* = 6.8 Hz, 2H), 3.69 (s, 2H), 2.48 (q, *J* = 7.2 Hz, 2H), 1.03 (t, *J* = 7.6 Hz, 3H). ^13^C{^1^H} NMR (100 MHz, CDCl_3_, ppm): δ 209.0, 134.5, 129.4, 128.7, 127.0, 49.8, 35.2, 7.8.

#### 4-*t*-Butylcyclohexanone (**3j**)

Following the general procedure, 4-*t*-butylcyclohexanol as mixture of isomers (**2j**) (238 mg, 1.52 mmol), **1** (20.9 mg, 0.0399 mmol), anhydrous trimethylamine *N*-oxide (3.0 mg, 0.040 mmol), and furfural (293 g, 3.1 mmol) in 3.1 mL toluene afforded 194 mg (83%) of **3j** as light-yellow crystals after flash chromatography (94% cyclohexane/6% ethyl acetate).^103^^1^H NMR (400 MHz, CDCl_3_, ppm): δ 2.43–2.31 (m, 4H), 2.10–2.05 (m, 2H), 1.53–1.39 (m, 3H), 0.92 (s, 9H). ^13^C{^1^H} NMR (100 MHz, CDCl_3_, ppm): δ 212.7, 46.7, 41.3, 32.5, 27.6.

#### *n*-Butyl-5-oxohexanamide (**3k**)

Following the general procedure, *n*-butyl-5-hydroxyhexanamide (**2k**) (249 mg, 1.33 mmol), **1** (17.5 mg, 0.033 mmol), anhydrous trimethylamine *N*-oxide (2.5 mg, 0.033 mmol), and furfural (256 mg, 2.66 mmol) in 2.7 mL toluene afforded 203 mg (82%) of **3k** as an off-white, amorphous solid after flash chromatography (5% cyclohexane/95% ethyl acetate). ^1^H NMR (400 MHz, CDCl_3_, ppm): δ 6.19 (br s, 1H), 3.23 (q, *J* = 6.8 Hz, 2H, 2.52 (t, *J* = 7.2 Hz, 2H), 2.20 (t, 7.2 Hz, 2H), 2.14 (s, 3H) 1.89 (quintet, *J* = 7.2 Hz, 2H), 1.48 (quintet, *J* = 7.2 Hz, 2H), 1.34 (sextet, *J* = 7.2 Hz, 2H), 0.92 (t, *J* = 7.2 Hz, 3H). ^13^C{^1^H} NMR (100 MHz, CDCl_3_, ppm): δ 208.6, 172.4, 42.5, 39.1, 35.3, 31.6, 29.8, 20.0, 19.7, 13.7. HRMS (EI) for C_10_H_19_NO_2_: calculated for M^+^*m*/*z* = 185.1416, found *m*/*z* = 185.1416.

#### p-Anisaldehyde (**3m**)

Following the general procedure, 4-methoxybenzyl alcohol (**2m**) (184 mg, 1.33 mmol), **1** (17.5 mg, 0.033 mmol), anhydrous trimethylamine *N*-oxide (2.5 mg, 0.033 mmol), and furfural (641 mg, 6.67 mmol) in 2.7 mL toluene afforded 166 mg (92%) of **3m** as a light-yellow oil after flash chromatography (94% cyclohexane/6% ethyl acetate).^86^^1^H NMR (400 MHz, CDCl_3_, ppm): δ 9.88 (s, 1H), 7.83 (d, *J* = 8.8 Hz, 2H), 7.00 (d, *J* = 8.8 Hz, 2H), 3.88 (s, 3H). ^13^C{^1^H} NMR (100 MHz, CDCl_3_, ppm): δ 190.8, 164.6, 132.0, 130.0, 114.3, 55.6.

#### 4-Phenylbenzaldehyde (**3n**)

Following the general procedure, 4-phenylbenzyl alcohol (**2n**) (286 mg, 1.55 mmol), **1** (20 mg, 0.039 mmol), anhydrous trimethylamine *N*-oxide (2.9 mg, 0.039 mmol), and furfural (746 mg, 7.77 mmol) in 3.1 mL toluene afforded 231 mg (82%) of **3n** as a white, microcrystalline solid after flash chromatography (99% cyclohexane/1% ethyl acetate).^99^^1^H NMR (400 MHz, CDCl_3_, ppm): δ 9.99 (s, 1H), 7.89 (d, *J* = 8.0 Hz, 2H), 7.68 (d, *J* = 8.0 Hz, 2H), 7.58 (d, *J* = 8.0 Hz, 2H), 7.43 (t, *J* = 7.2 Hz, 2H), 7.37 (t, *J* = 7.2 Hz, 1H). ^13^C{^1^H} NMR (100 MHz, CDCl_3_, ppm): δ 191.9, 147.1, 139.7, 135.3, 130.3, 129.1, 128.5, 127.7, 127.4.

#### 4-Bromobenzaldehyde (**3o**)

Following the general procedure, 4-bromobenzyl alcohol (**2o**) (426 mg, 2.28 mmol), **1** (30 mg, 0.057 mmol), anhydrous trimethylamine *N*-oxide (4.3 mg, 0.057 mmol), and furfural (1.10 g, 11.4 mmol) in 4.6 mL toluene afforded 271 mg (64%) of **3o** as a pale purple, microcrystalline solid (due to coelution with tetraphenylcyclopentadienone) after flash chromatography (97% cyclohexane/3% ethyl acetate).^100^^1^H NMR (400 MHz, CDCl_3_, ppm): δ 9.98 (s, 1H), 7.75 (d, *J* = 7.6 Hz, 2H), 7.69 (d, *J* = 8.0 Hz, 2H). ^13^C{^1^H} NMR (100 MHz, CDCl_3_, ppm): δ 191.1, 135.1, 132.4, 131.0, 129.8.

#### Methyl 4-Formylbenzoate (**3p**)

Following the general procedure, methyl 4-(hydroxymethyl)benzoate (**2p**) (258 mg, 1.55 mmol), **1** (20 mg, 0.039 mmol), anhydrous trimethylamine *N*-oxide (2.9 mg, 0.039 mmol), and furfural (746 mg, 7.77 mmol) in 3.1 mL toluene afforded 329 mg of a mixture of **3p** and furfural^86^ as a pale yellow oil in a 1:3 molar ratio (which corresponds to 119 mg of **3p** (46%)) after flash chromatography (88% cyclohexane/12% ethyl acetate).^104^ Spectroscopic data provided is for **3p**. ^1^H NMR (400 MHz, CDCl_3_, ppm): δ 10.11 (s, 1H), 8.20 (d, *J* = 8.0 Hz, 2H), 7.96 (d, *J* = 8.0 Hz, 2H), 3.96 (s, 3H). ^13^C{^1^H} NMR (100 MHz, CDCl_3_, ppm): δ 191.7, 166.0, 139.1, 135.0, 130.1, 129.5, 52.5.

#### 4-Chloro-3-fluorobenzaldehyde (**3q**)

Following the general procedure, 4-chloro-3-fluorobenzyl alcohol (**2q**) (366 mg, 2.28 mmol), **1** (30 mg, 0.057 mmol), anhydrous trimethylamine *N*-oxide (4.3 mg, 0.057 mmol), and furfural (1.10 g, 11.4 mmol) in 4.6 mL toluene afforded 150 mg (41%) of **3q** as an off-white, amorphous solid after flash chromatography (97% cyclohexane/3% ethyl acetate).^105^^1^H NMR (400 MHz, CDCl_3_, ppm): δ 9.96 (d, *J* = 1.6 Hz, 1H), 7.58–7.66 (m, 3H). ^13^C{^1^H} NMR (100 MHz, CDCl_3_, ppm): δ 189.7 (d, *J* = 1.8 Hz), 158.6 (d, *J* = 250.9 Hz), 136.5 (d, *J* = 5.5 Hz), 131.5, 128.0 (d, *J* = 18.1 Hz), 126.3 (d, *J* = 3.7 Hz), 116.4 (d, *J* = 21.4 Hz).

#### 2-Thiophenecarboxaldehyde (**3s**)

Following the general procedure, thiophen-2-ylmethanol (**2s**) (348 mg, 3.05 mmol), **1** (40 mg, 0.076 mmol), anhydrous trimethylamine *N*-oxide (5.7 mg, 0.076 mmol), and furfural (1.47 g, 15.3 mmol) in 6.1 mL toluene afforded 173 mg (52%) of **3s** as a yellow oil after flash chromatography (94% cyclohexane/6% ethyl acetate).^86^^1^H NMR (400 MHz, CDCl_3_, ppm): δ 9.95 (s, 1H), 7.79 (d, *J* = 4.0 Hz, 1H), 7.78 (d, *J* = 5.2 Hz, 1H), 7.23 (t, *J* = 4.0 Hz, 1H). ^13^C{^1^H} NMR (100 MHz, CDCl_3_, ppm): δ 183.1, 144.0, 136.4, 135.2, 128.4.

#### Cinnamaldehyde (**3t**)

Following the general procedure, cinnamyl alcohol (**2t**) (306 mg, 2.28 mmol), **1** (30 mg, 0.057 mmol), anhydrous trimethylamine *N*-oxide (4.3 mg, 0.057 mmol), and furfural (1.10 g, 11.4 mmol) in 4.6 mL toluene afforded 239 mg (79%) of **3t** as a light-yellow oil after flash chromatography (95% cyclohexane/5% ethyl acetate).^86^^1^H NMR (400 MHz, CDCl_3_, ppm): δ 9.70 (d, *J* = 7.6 Hz, 1H), 7.55–7.57 (m, 2H), 7.47 (d, *J* = 16.0 Hz, 1H), 7.42–7.44 (m, 3H), 6.71 (dd, *J* = 7.6, 16 Hz, 1H). ^13^C{^1^H} NMR (100 MHz, CDCl_3_, ppm): δ 193.8, 152.8, 134.0, 131.3, 129.1, 128.6, 128.5.

#### Geranial (**3u**)

Following the general procedure, geraniol (**2u**) (352 mg, 2.28 mmol), **1** (30 mg, 0.057 mmol), anhydrous trimethylamine *N*-oxide (4.3 mg, 0.057 mmol), and furfural (1.10 g, 11.4 mmol) in 4.6 mL toluene afforded 323 mg (93%) of **3u** (10:1 ratio with neral) as a colorless oil after flash chromatography (97% cyclohexane/3% ethyl acetate).^86^^1^H NMR (400 MHz, CDCl_3_, ppm): δ 9.99 (d, *J* = 8.4 Hz, 1H), 5.88 (dd, *J* = 1.2, 8.0 Hz, 1H), 5.06–5.09 (m, 1H), 2.19–2.27 (m, 4H), 2.17 (d, *J* = 1.2 Hz, 3H), 1.69 (s, 3H), 1.61 (s, 3H). ^13^C{^1^H} NMR (100 MHz, CDCl_3_, ppm): δ 191.2, 163.8, 132.8, 127.4, 122.6, 40.6, 25.7, 25.6, 17.7, 17.5.

#### Neral (**3v**)

Following the general procedure, nerol (**2v**) (352 mg, 2.28 mmol), **1** (30 mg, 0.057 mmol), anhydrous trimethylamine *N*-oxide (4.3 mg, 0.057 mmol), and furfural (1.10 g, 11.4 mmol) in 4.6 mL toluene afforded 305 mg (88%) of **3v** (10:1 ratio with geranial) as a colorless oil after flash chromatography (97% cyclohexane/3% ethyl acetate).^106^^1^H NMR (400 MHz, CDCl_3_, ppm): δ 9.90 (d, *J* = 8.4 Hz, 1H), 5.88 (d, *J* = 8.4 Hz, 1H), 5.10 (t, *J* = 7.2 Hz, 1H), 2.59 (t, *J* = 7.6 Hz, 2H), 2.24 (q, *J* = 7.6 Hz, 2H), 1.99 (s, 3H), 1.68 (s, 3H), 1.60 (s, 3H). ^13^C{^1^H} NMR (100 MHz, CDCl_3_, ppm): δ 190.8, 163.8, 133.6, 128.6, 122.3, 32.5, 27.0, 25.6, 25.0, 17.7.

#### Myrtenal (**3w**)

Following the general procedure, myrtenol (**2w**) (347 mg, 2.28 mmol), **1** (30 mg, 0.057 mmol), anhydrous trimethylamine *N*-oxide (4.3 mg, 0.057 mmol), and furfural (1.10 g, 11.4 mmol) in 4.6 mL toluene afforded 271 mg (79%) of **3w** as a colorless oil after flash chromatography (97% cyclohexane/3% ethyl acetate).^107^^1^H NMR (400 MHz, CDCl_3_, ppm): δ 9.44 (s, 1H), 6.70–6.73 (m, 1H), 2.87 (t, *J* = 5.6 Hz, 1H), 2.57 (td, *J* = 3.2, 10.0 Hz, 2H), 2.49 (td, *J* = 5.6, 9.2 Hz, 1H), 2.19–2.20 (m, 1H), 1.34 (s, 3H), 1.06 (d, *J* = 9.2 Hz, 1H), 0.75 (s, 3H). ^13^C{^1^H} NMR (100 MHz, CDCl_3_, ppm): δ 191.2, 151.5, 147.8, 40.7, 38.1, 37.6, 33.0, 31.1, 25.6, 20.9.

### General Oxidation and Isolation Procedure Using Acetone as the Hydrogen Acceptor

A solution of alcohol (1 equiv), catalyst **1** (0.025 equiv), and anhydrous trimethylamine *N*-oxide (0.025 equiv) in acetone (0.5 M in alcohol) were stirred in a 60 °C oil bath in a round-bottom flask with a condenser or in an 80 °C oil bath in a thick-walled, glass tube with a PTFE screw cap for 24 h (Table 3, Conditions 2 and 3). **Caution!***Elevated pressure could cause the tube to explode. Perform the reaction in a fume hood behind a blast shield.* The volatiles were removed under reduced pressure and the crude product was purified by flash chromatography using the same eluent system noted in the above procedures.

### Oxidation of 2-Heptanol Monitored by ^1^H NMR Spectroscopy

2-Heptanol (32 mg, 0.27 mmol) and furfural (52 mg, 0.54 mmol) were added to a solution of **4** (15 mg, 0.027 mmol) and anhydrous trimethylamine *N*-oxide (2.5 mg, 0.033 mmol) in 0.7 mL benzene-*d*_6_ (Figure 4). The solution stirred at rt for 10 min and at 60 °C for 2 min and was transferred to a screw-cap NMR tube under nitrogen. A ^1^H NMR spectrum was collected at 27 °C. The NMR tube was heated to 70 °C in the spectrometer, and ^1^H NMR spectra were collected at 10, 20, and 30 min after the sample reached 70 °C. The observed spectral range was from 15 to −30 ppm.

### General Oxidation Procedure for Determining Reaction Selectivity Using Furfural as the Hydrogen Acceptor

A solution of primary alcohol (1 equiv), secondary alcohol (1 equiv), and biphenyl (0.25 equiv) was prepared in toluene (0.5 M in primary alcohol) (Tables 4–8). A 50 μL aliquot was removed, diluted with 1 mL of acetone, and analyzed by gas chromatography to give the *t* = 0 chromatogram. Furfural (1 equiv when **1** was used or 5 equiv when **11**, **14**, or **15** were used), iron catalyst (0.05 equiv), and anhydrous trimethylamine *N*-oxide (0.06 equiv when **1** was used or 0.15 equiv when **11**, **14**, or **15** was used) were added and the reaction stirred at 80 °C. After 24 h, a 200 μL sample of the reaction solution was diluted with 1 mL hexanes. Residual iron was removed from the sample by adding it to a Pasteur pipet half filled with silica gel and eluting with 4 mL 1:1 hexanes/ethyl acetate. A 1.2 mL sample of the eluted solution was analyzed by gas chromatography. Conversion was determined based on how much reactant had been consumed compared to the amount of reactant in the *t* = 0 chromatogram relative to the internal standard (biphenyl).

### General Oxidation Procedure for Determining Reaction Selectivity Using Acetone as the Hydrogen Acceptor

A solution of primary alcohol (1 equiv), secondary alcohol (1 equiv), and biphenyl (0.25 equiv) was prepared in acetone (0.5 M in primary alcohol) (Tables 4 and 6–8). A 50 μL aliquot was removed, diluted with 1 mL of acetone, and analyzed by gas chromatography to give the *t* = 0 chromatogram. Iron catalyst (0.05 equiv) and anhydrous trimethylamine *N*-oxide (0.06 equiv when **1** was used or 0.15 equiv when **11**, **14**, or **15** was used) were added and the reaction stirred in a 60 °C oil bath. After 24 h, a 200 μL sample of the reaction solution was diluted with 1 mL hexanes. Residual iron was removed from the sample by adding it to a Pasteur pipet half filled with silica gel and eluting with 4 mL 1:1 hexanes/ethyl acetate. A 1.2 mL sample of the eluted solution was analyzed by gas chromatography. Conversion was determined based on how much reactant had been consumed compared to the amount of reactant in the *t* = 0 chromatogram relative to the internal standard (biphenyl).

### General Diol Oxidation Procedure Using Furfural as the Hydrogen Acceptor

A solution of diol **16-Ph** or **16-Me** (1 equiv), furfural (1 equiv when **1** was used or 5 equiv when **11** or **14** were used), iron catalyst (0.05 equiv), and anhydrous trimethylamine *N*-oxide (0.06 equiv when **1** was used or 0.15 equiv when **11** or **14** was used) in toluene (0.5 M in diol) was stirred at 80 °C for 24 h (Table 9). The reaction mixture was filtered through Celite, washed with ethyl acetate, and the volatiles were removed under reduced pressure. A ^1^H NMR spectrum (400 MHz, CDCl_3_) of the crude product was taken and conversions were determined based on relative integrations of specific peaks corresponding to compounds **16**–**19**. **16-Me**(108): 4.89 ppm (q, 1H). **17-Me**(86): 9.97 ppm (s, 1H). **18-Me**(109): 4.76 ppm (s, 2H). **19-Me**(110): 10.09 ppm (s, 1H). **16-Ph**(111): 5.84 ppm (s, 1H). **17-Ph**(112): 9.96 ppm (s, 1H). **18-Ph**(113): 4.79 ppm (s, 2H). **19-Ph**(114): 10.12 ppm (s, 1H).

### General Diol Oxidation Procedure Using Acetone as the Hydrogen Acceptor

A solution of diol **16-Ph** or **16-Me** (1 equiv), iron catalyst (0.05 equiv), and anhydrous trimethylamine *N*-oxide (0.06 equiv when **1** was used or 0.15 equiv when **11** or **14** was used) in acetone (0.5 M in diol) was stirred at reflux for 24 h (Table 9). The reaction mixture was filtered through Celite, washed with ethyl acetate, and the volatiles were removed under reduced pressure. A ^1^H NMR spectrum (400 MHz, CDCl_3_) of the crude product was taken and conversions were determined based on relative integrations of specific peaks corresponding to compounds **16**–**19**. **16-Me**(108): 4.89 ppm (q, 1H). **17-Me**(86): 9.97 ppm (s, 1H). **18-Me**(109): 4.66 ppm (s, 2H). **19-Me**(110): 10.09 ppm (s, 1H). **16-Ph**(111): 5.84 ppm (s, 1H). **17-Ph**(112): 9.96 ppm (s, 1H). **18-Ph**(113): 4.79 ppm (s, 2H). **19-Ph**(114): 10.12 ppm (s, 1H).

### General Procedure for Monitoring Competitive Oxidation Reactions over Time Using Furfural as the Hydrogen Acceptor

A solution of primary alcohol (1 equiv), secondary alcohol (1 equiv), and biphenyl (0.25 equiv) was prepared in toluene (0.5 M in primary alcohol) (Figures 6–8). A 50 μL aliquot was removed, diluted with 1 mL of acetone, and analyzed by gas chromatography to give the *t* = 0 chromatogram. Furfural (1 equiv when **1** was used or 5 equiv when **11** was used), iron catalyst (0.05 equiv), and anhydrous trimethylamine *N*-oxide (0.06 equiv when **1** was used or 0.15 equiv when **11** was used) were added and the reaction stirred at 80 °C. Reaction samples (200 μL) were removed at the desired times and diluted with 1 mL of hexanes. Residual iron was removed from the sample by adding it to a Pasteur pipet half filled with silica gel and eluting with 4 mL 1:1 hexanes/ethyl acetate. A 1.2 mL sample of the eluted solution was analyzed by gas chromatography. Conversion was determined based on how much reactant had been consumed compared to the amount of reactant in the *t* = 0 chromatogram relative to the internal standard (biphenyl).

### Monitoring the Competitive Oxidation of Cinnamyl Alcohol and 4-Phenyl-2-butanol over Time Using Acetone as the Hydrogen Acceptor

A solution of cinnamyl alcohol (336 mg, 2.5 mmol), 4-phenyl-2-butanol (376 mg, 2.5 mmol), and biphenyl (96.4 mg, 0.625 mmol) was prepared in 5 mL of acetone (Figure 7). A 50 μL aliquot was removed, diluted with 1 mL of acetone, and analyzed by gas chromatography to give the *t* = 0 chromatogram. Catalyst **1** (65.5 mg, 0.125 mmol) and anhydrous trimethylamine *N*-oxide (11.3 mg, 0.15 mmol) were added and the reaction stirred at reflux. Reaction samples (200 μL) were removed at the desired times and diluted with 1 mL of hexanes. Residual iron was removed from the sample by adding it to a Pasteur pipet half filled with silica gel and eluting with 4 mL 1:1 hexanes/ethyl acetate. A 1.2 mL sample of the eluted solution was analyzed by gas chromatography. Conversion was determined based on how much reactant had been consumed compared to the amount of reactant in the *t* = 0 chromatogram relative to the internal standard (biphenyl).

## Data Availability

The data underlying this study are available in the published article and its Supporting Information.

## Supporting Information Available

Solvent screen table, NMR spectra for all compounds, and example NMR spectra for reaction monitoring and diol oxidation selectivity (PDF)
