# Supplementary material for: Selectivity Effects of Hydrogen Acceptors and Catalyst Structures in Alcohol Oxidations Using (Cyclopentadienone)iron Tricarbonyl Compounds
Source: J Org Chem. 2025 Jan 23;90(5):2036–51. doi: 10.1021/acs.joc.4c02846 (PMC11812019; doi:10.1021/acs.joc.4c02846)

Supporting Information

Selectivity Effects of Hydrogen Acceptors and Catalyst Structures in Alcohol Oxidations Using (Cyclopentadienone)iron Tricarbonyl Compounds

Melanie Hempel, Auden Cameron Lampariello, Nicolle Elahian López, Cole Springer, Kimberly McCaskey, Sneha Jayaram, Kathryn M. J. Wnuk-Fink, Bryn K. Werley, and Timothy W. Funk

Department of Chemistry, Gettysburg College, Gettysburg, Pennsylvania, 17325, United States

Corresponding author: tfunk@gettysburg.edu

Contents:

General procedure for the solvent screen of the Oppenauer-type oxidation S2

Solvent screen using 4-phenyl-2-butanol Oppenauer-type oxidation (Table S1) S2

NMR spectroscopy general information S2

NMR spectra (Figures S1–S54) S3–S56

General procedure for solvent screen (Table S1). A solution of 4-phenyl-2-butanol (1 equiv) and biphenyl (0.25 equiv) was prepared in the desired solvent (0.5 M in 4-phenyl-2-butanol). A 50 µL aliquot was removed, diluted with 1 mL of acetone, and analyzed by gas chromatography to give the t = 0 chromatogram. Furfural (2 equiv), **1** (0.025 equiv), and anhydrous trimethylamine *N*-oxide (0.025 equiv) were added and the reaction was submerged in an 80 °C oil bath. After stirring for 24 h, a 200 µL sample of the reaction solution was diluted with 1 mL hexanes. Residual iron was removed from the sample by adding it to a Pasteur pipet half filled with silica gel and eluting with 4 mL 1:1 hexanes/ethyl acetate. A 1.2 mL sample of the eluted solution was analyzed by gas chromatography. Conversion was determined based on how much reactant had been consumed compared to the amount of reactant in the t = 0 chromatogram relative to the internal standard (biphenyl).

Table S1. Solvent screen of 4-phenyl-2-butanol Oppenauer-type oxidation with **1**.

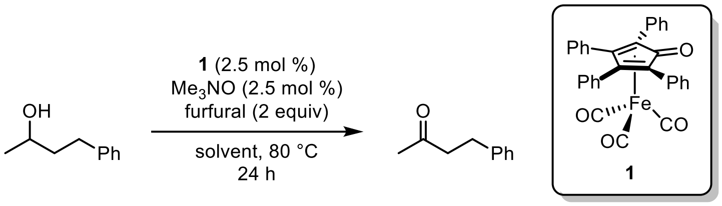

entry	solvent	conversion (%)
1	toluene	98
2	*tert*-amyl alcohol	53
3	2-methyltetrahydrofuran	55
4	ethyl acetate	80
5	isopropyl acetate	75
6	*tert*-butanol	63
7	furfural	57

NMR Spectra

All ^{1}H and ^{13}C{^{1}H} NMR spectra were recorded at ambient temperature at 400 MHz and 100 MHz, respectively, on a Bruker Avance Neo 400 MHz FT-NMR spectrometer unless otherwise noted. Chemical shifts are reported in parts per million (ppm) relative to tetramethylsilane (TMS) for spectra taken in $CDCl_3$. ^{1}H NMR spectra taken in benzene-d_6 used the residual solvent peak at 7.16 ppm as a reference. Samples were prepared in 0.7 mL of solvent unless otherwise noted.

Figure S1. ^{1}H NMR spectrum (400 MHz, $CDCl_3$) of **2k**

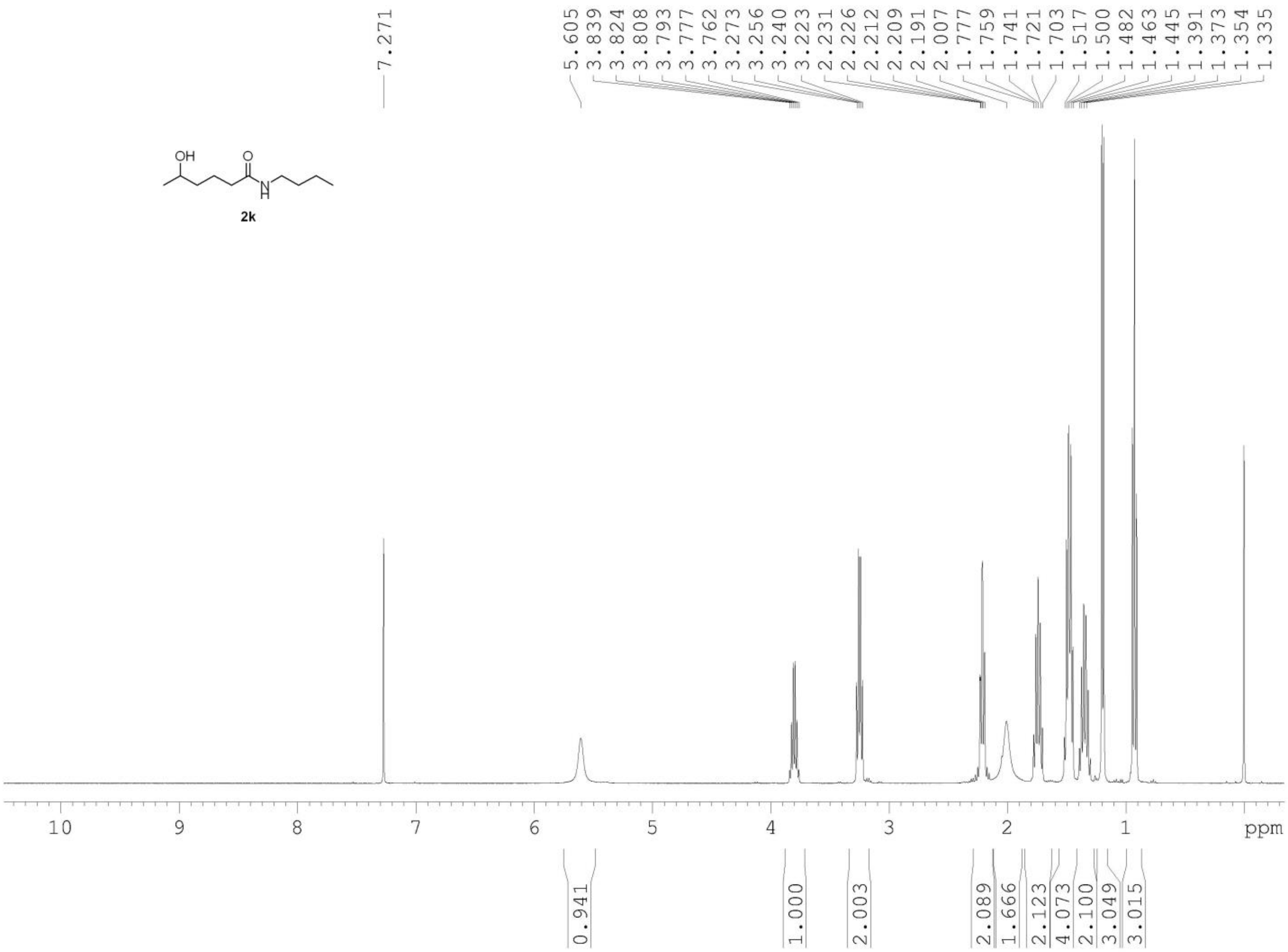

Figure S2. $^{13}C\{^{1}H\}$ NMR spectrum (100 MHz, $CDCl_3$) of **2k**

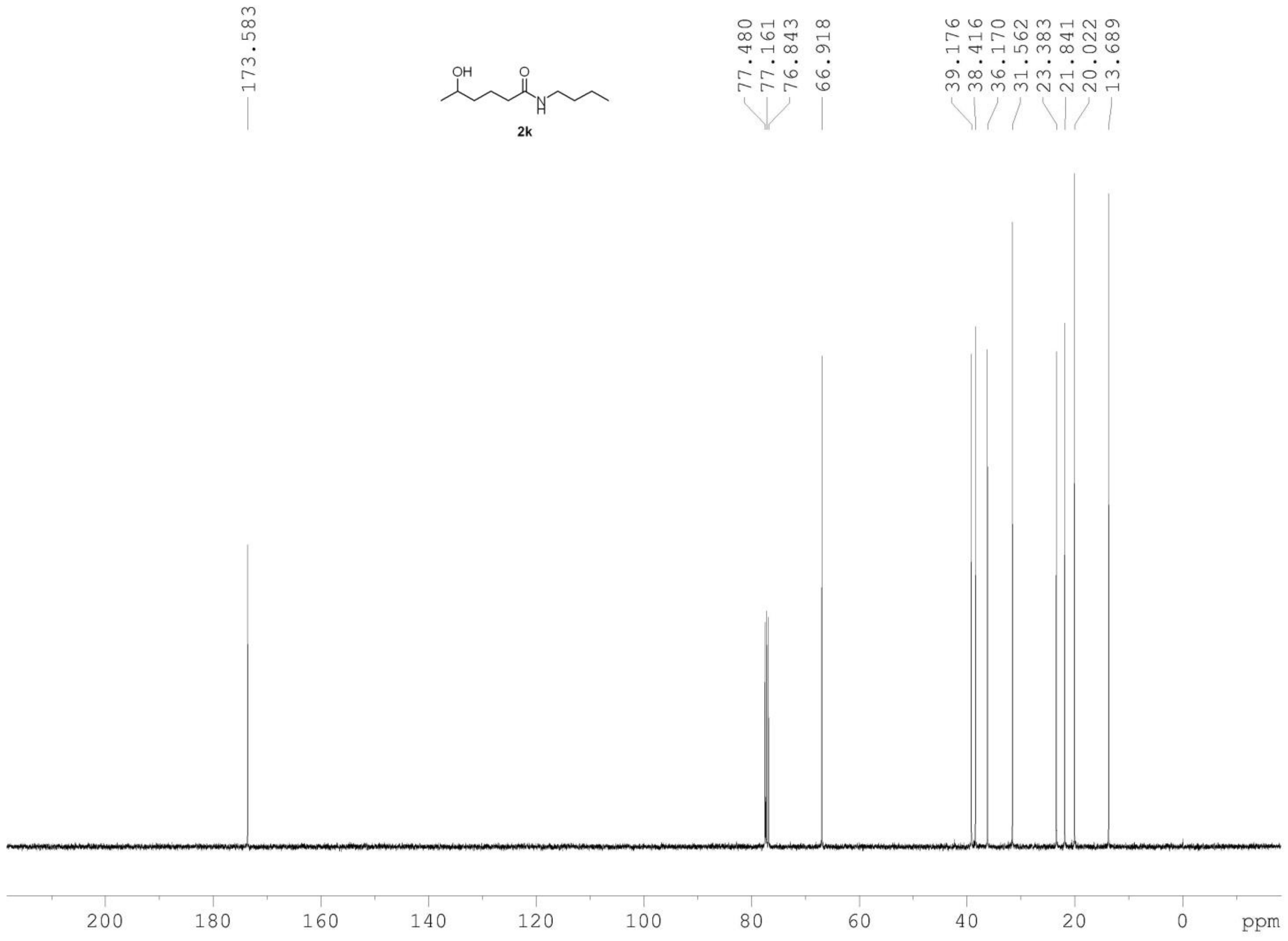

Figure S3. ^{1}H NMR spectrum (400 MHz, $CDCl_3$) of **13**

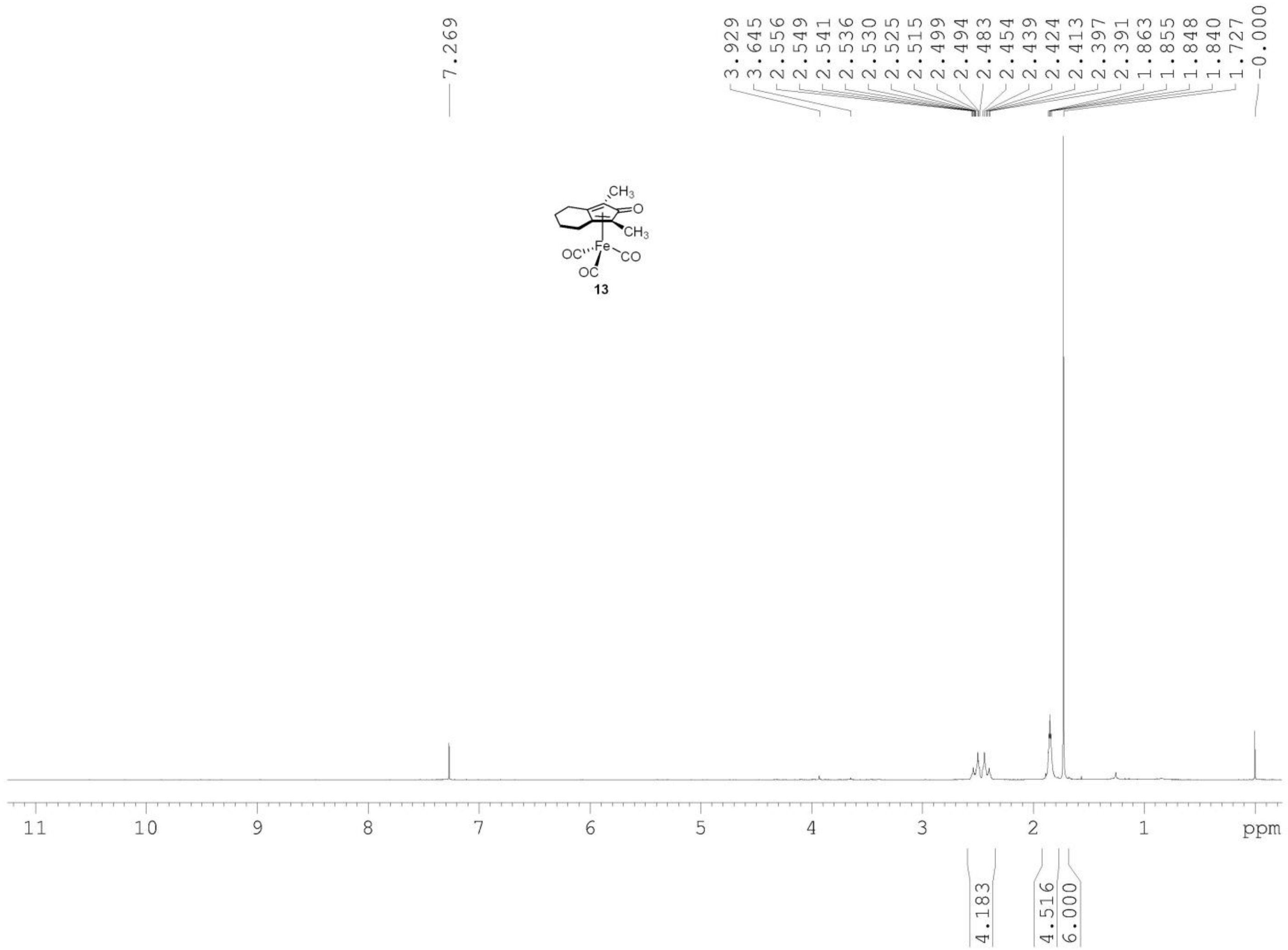

Figure S4. $^{13}C\{^{1}H\}$ NMR spectrum (100 MHz, $CDCl_3$) of **13**

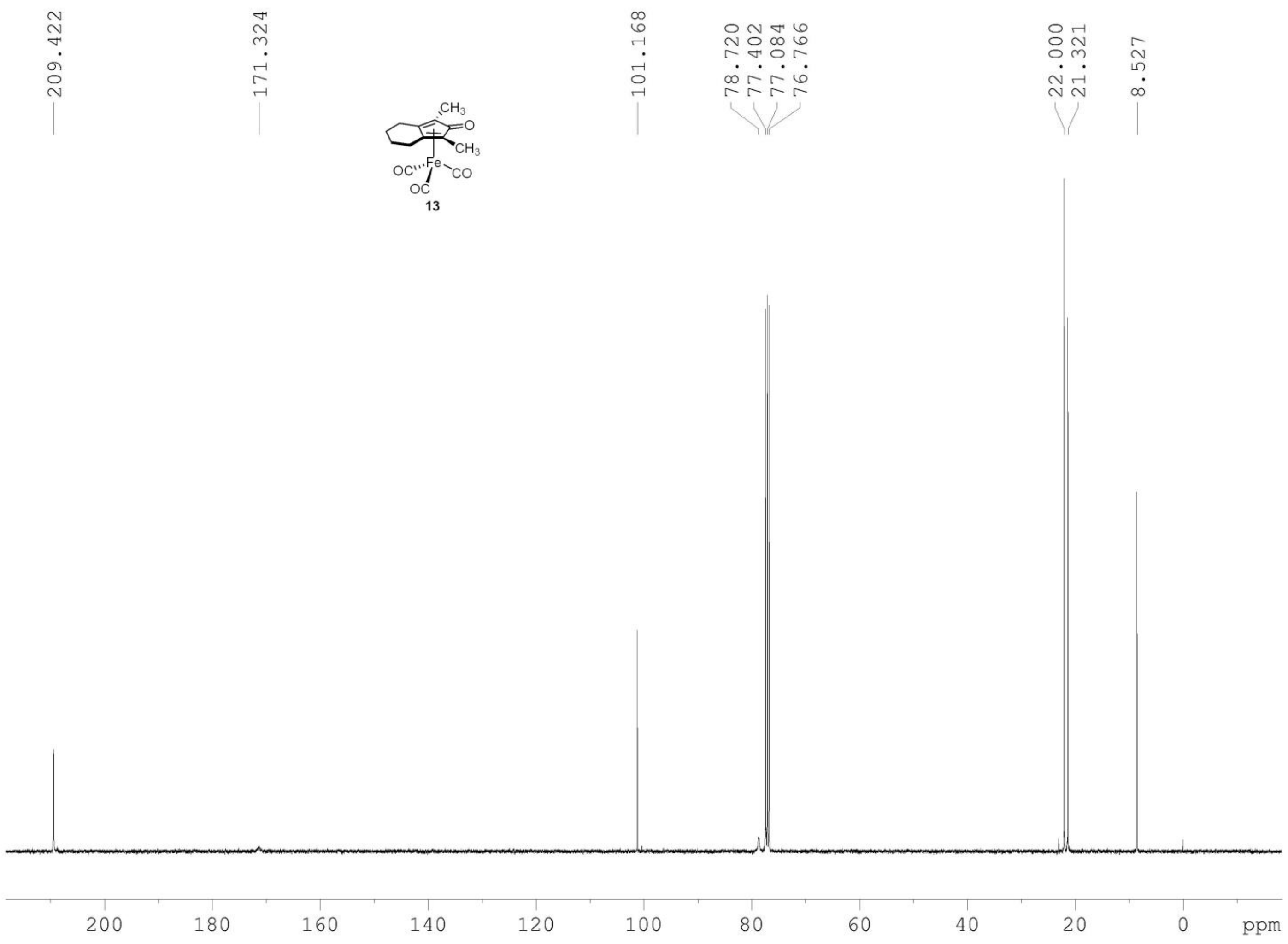

Figure S5. ^{1}H NMR spectrum (400 MHz, $CDCl_3$) of 1,7-bis(triethylsilyl)-1,6-heptadiyne

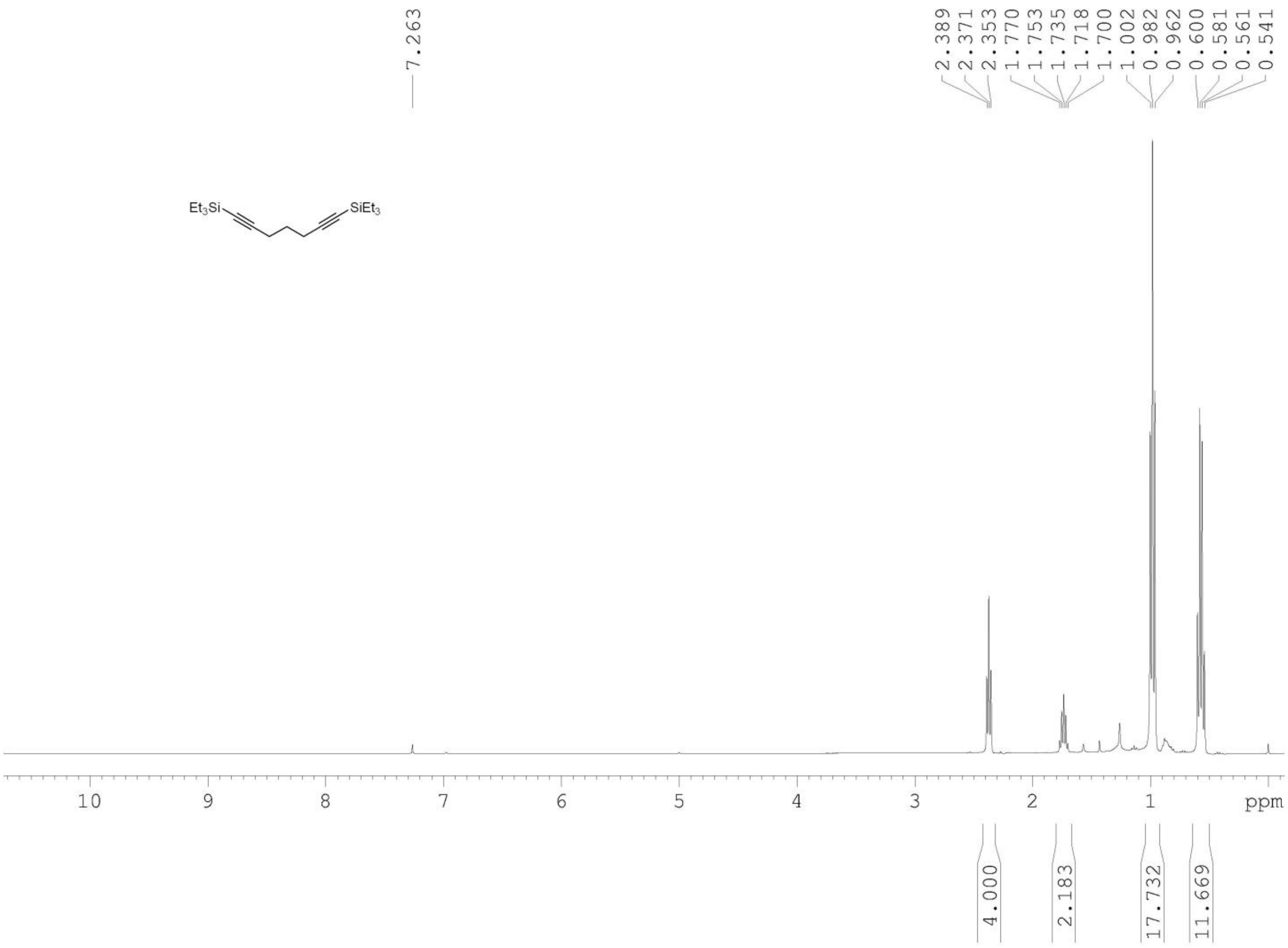

Figure S6. $^{13}C\{^{1}H\}$ NMR spectrum (100 MHz, $CDCl_3$) of 1,7-bis(triethylsilyl)-1,6-heptadiyne

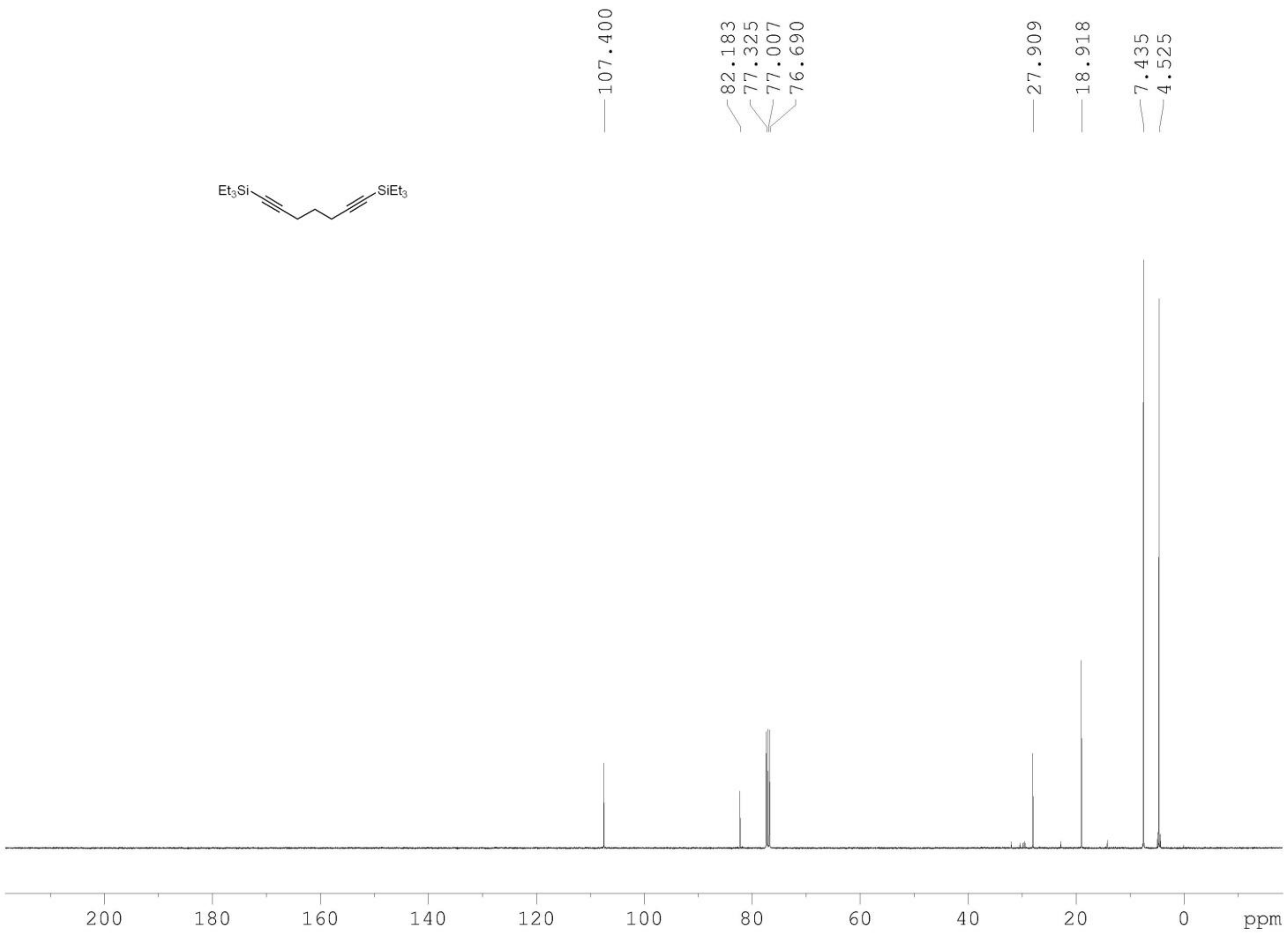

Figure S7. ^{1}H NMR spectrum (400 MHz, $CDCl_3$) of **14**

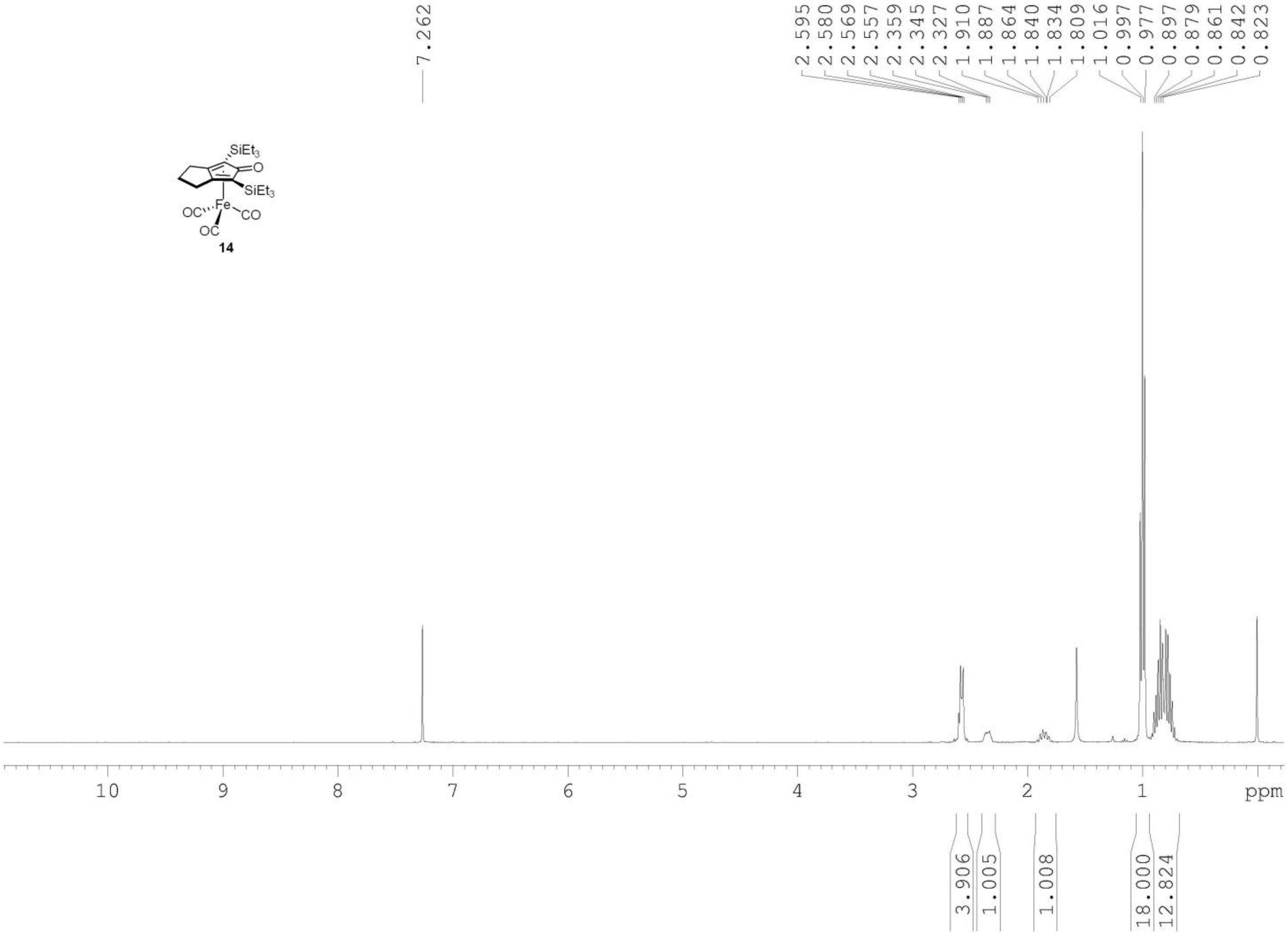

Figure S8. $^{13}C\{^{1}H\}$ NMR spectrum (100 MHz, $CDCl_3$) of **14**

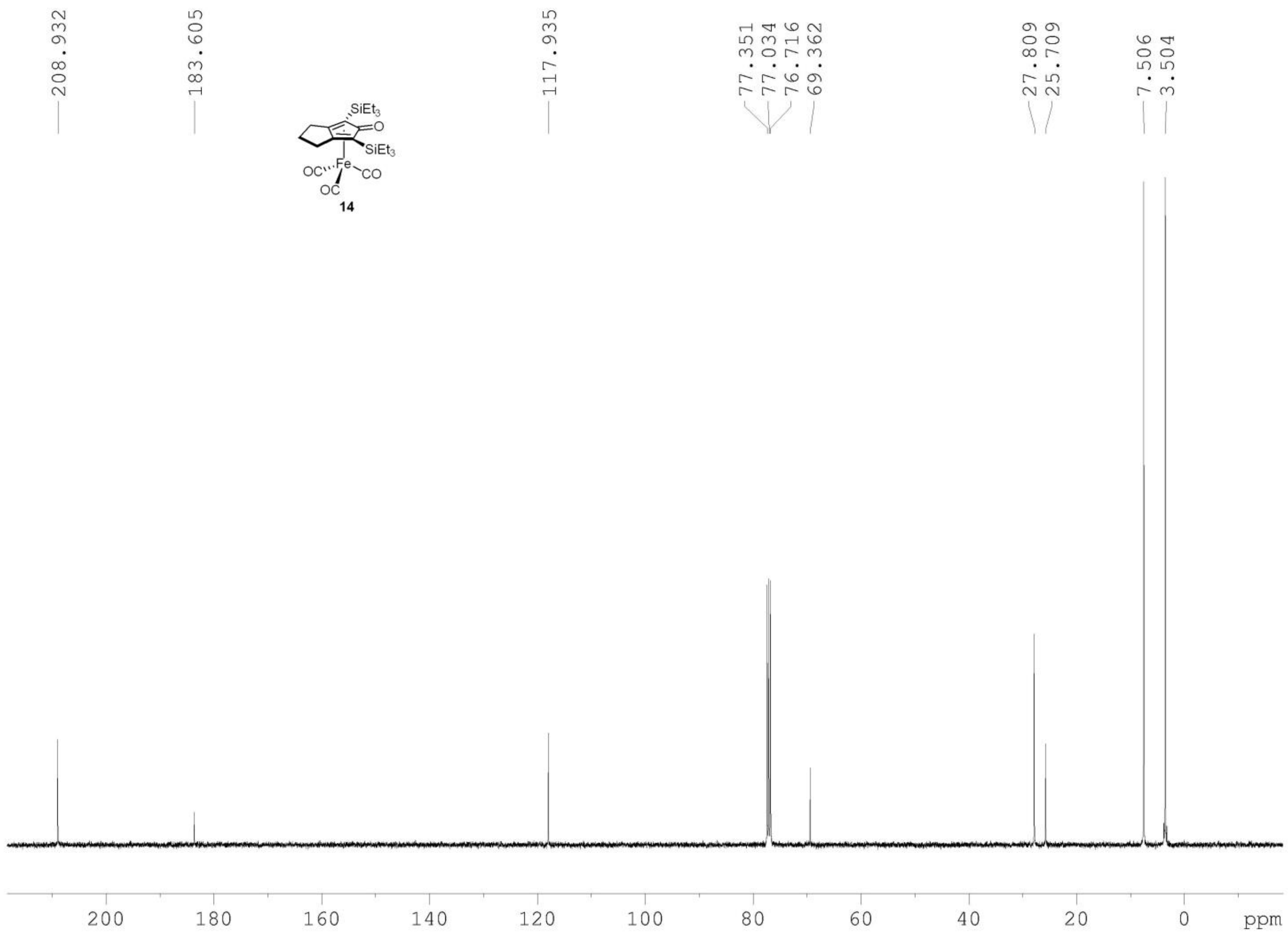

Figure S9. ^{1}H NMR spectrum (400 MHz, $CDCl_3$) of 4-phenyl-2-butanone

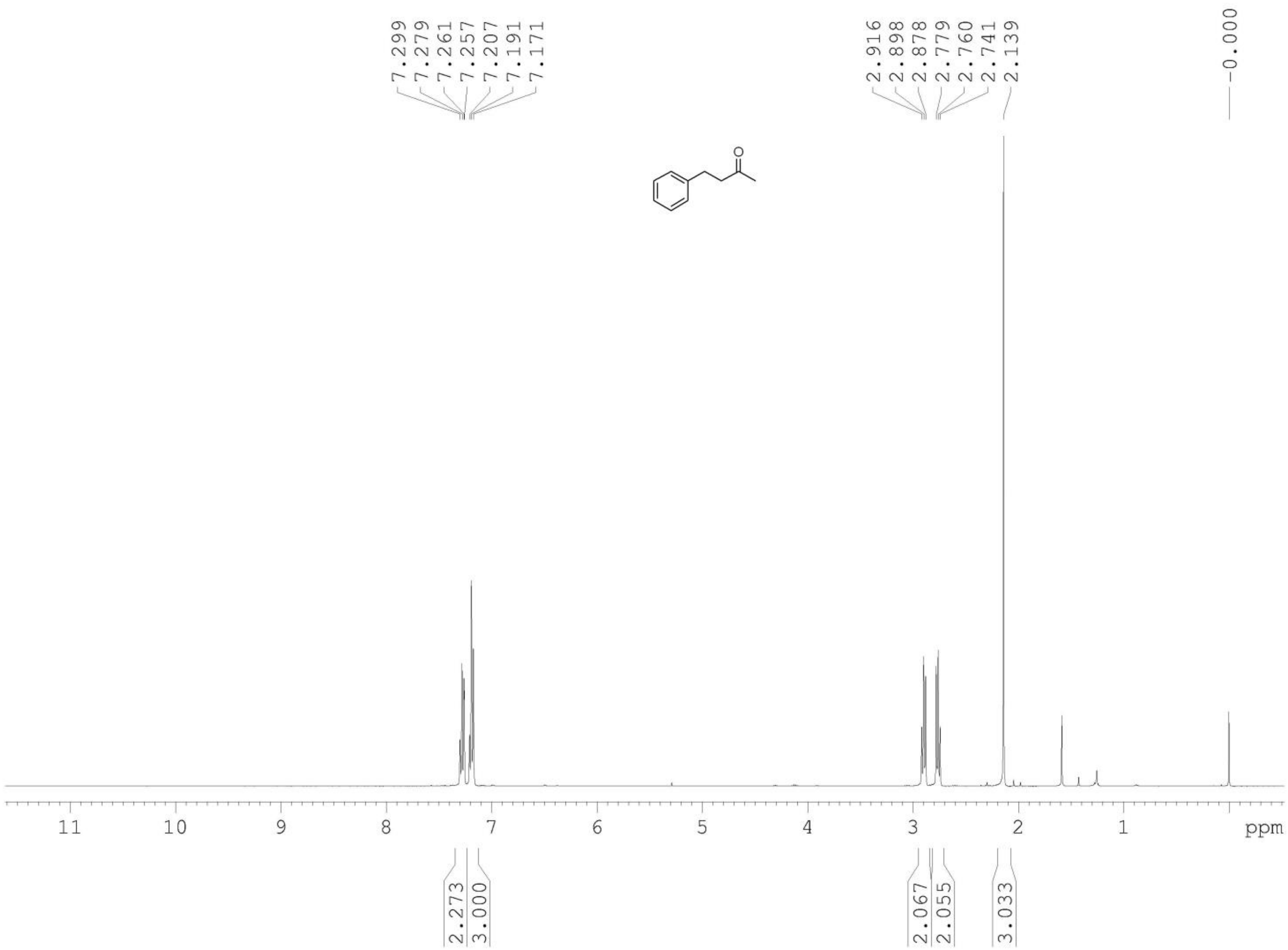

Figure S10. $^{13}C\{^{1}H\}$ NMR spectrum (100 MHz, $CDCl_3$) of 4-phenyl-2-butanone

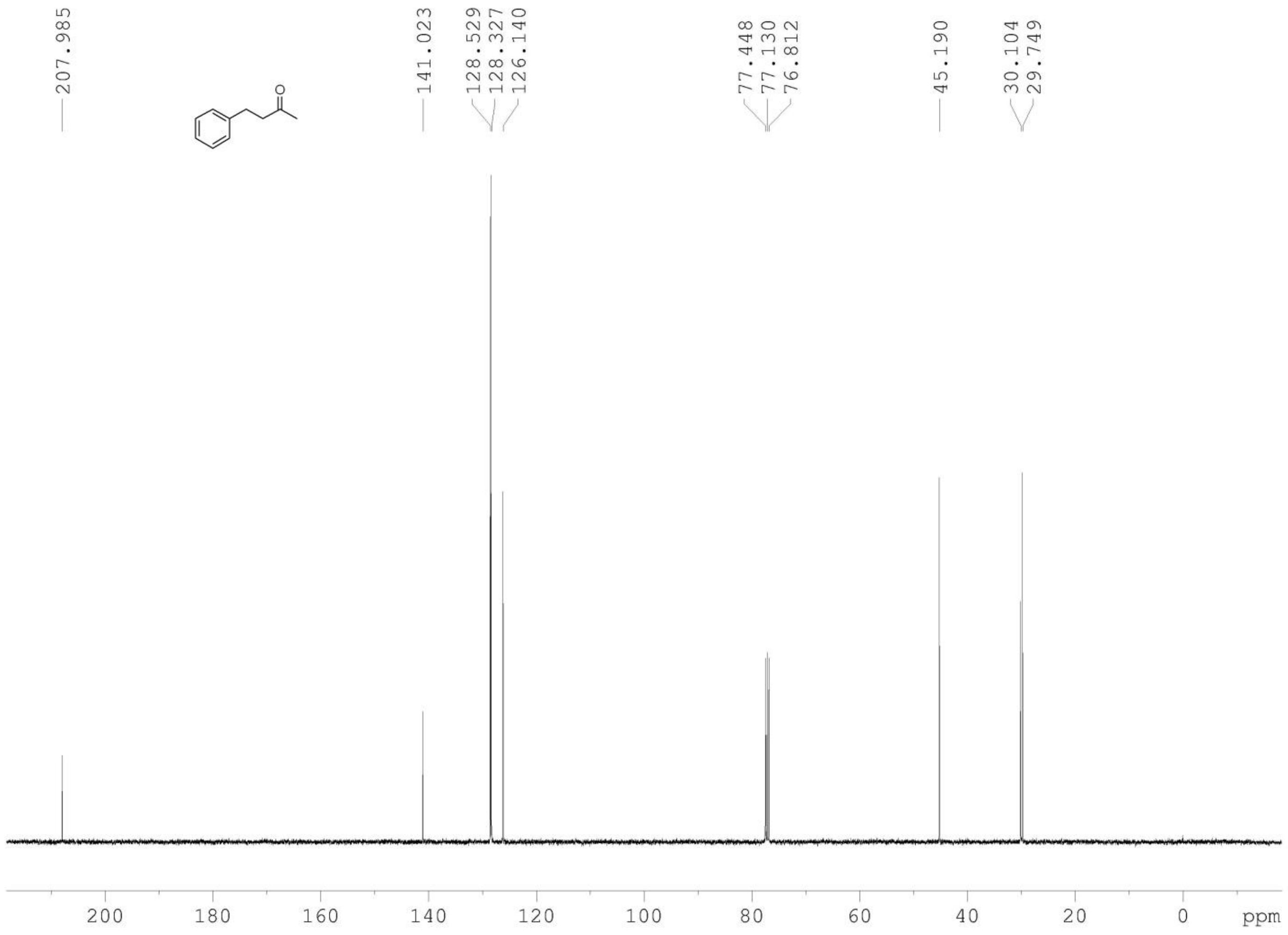

Figure S11. ^{1}H NMR spectrum (400 MHz, $CDCl_3$) of **3a**

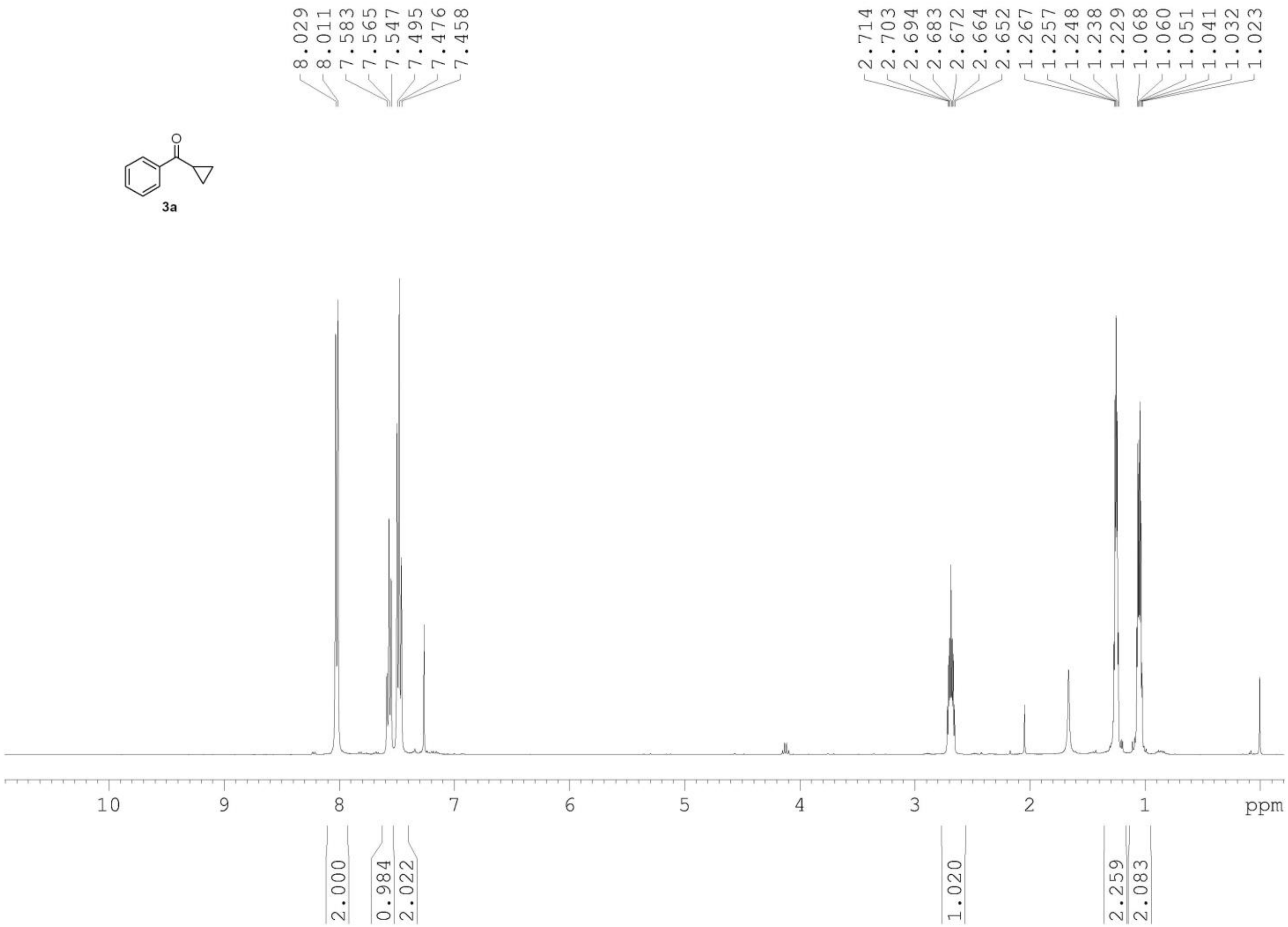

Figure S12. $^{13}C\{^{1}H\}$ NMR spectrum (100 MHz, $CDCl_3$) of **3a**

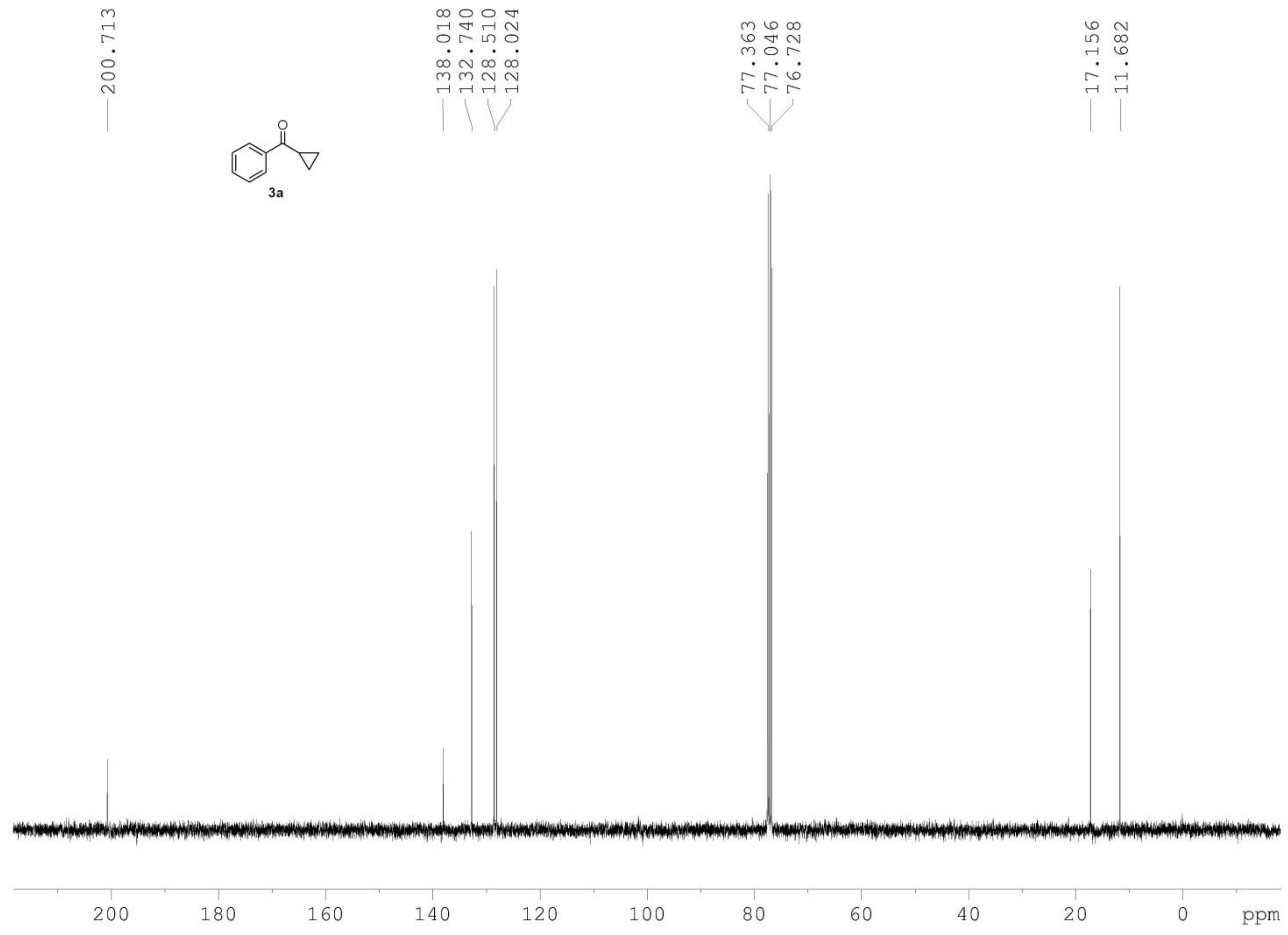

Figure S13. ^{1}H NMR spectrum (400 MHz, $CDCl_3$) of **3b**

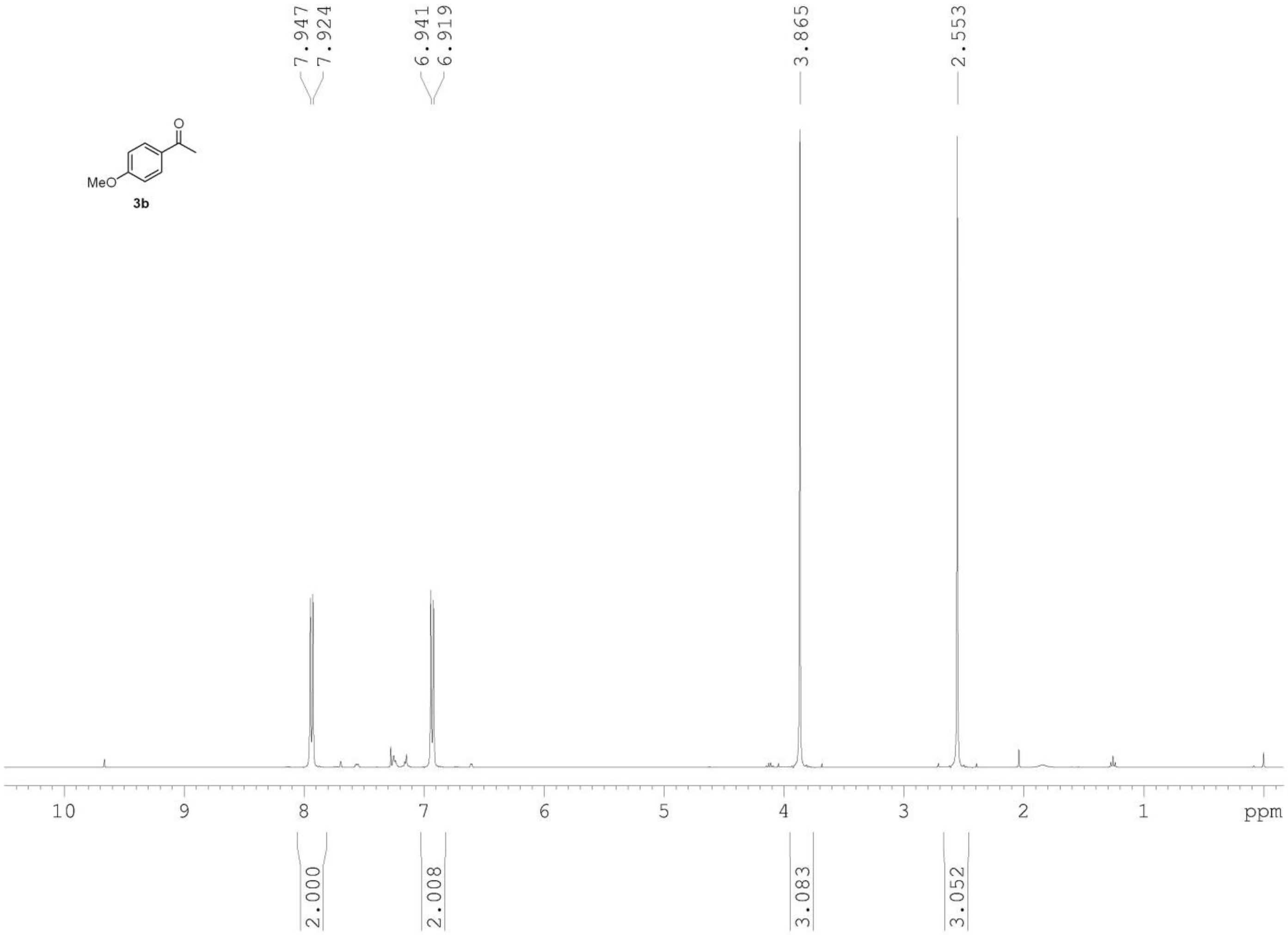

Figure S14. $^{13}C\{^{1}H\}$ NMR spectrum (100 MHz, $CDCl_3$) of **3b**

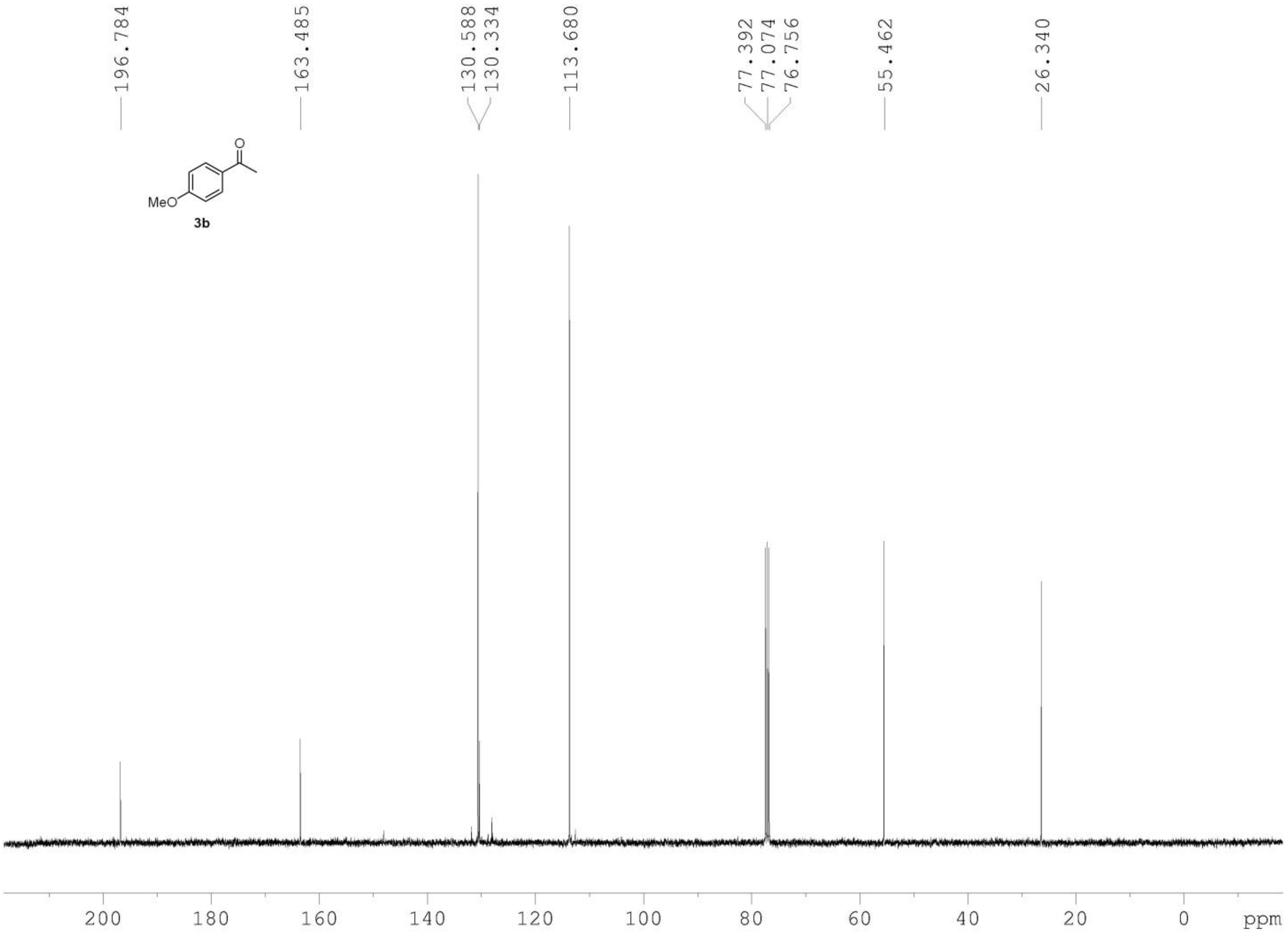

Figure S15. ^{1}H NMR spectrum (400 MHz, $CDCl_3$) of **3c**

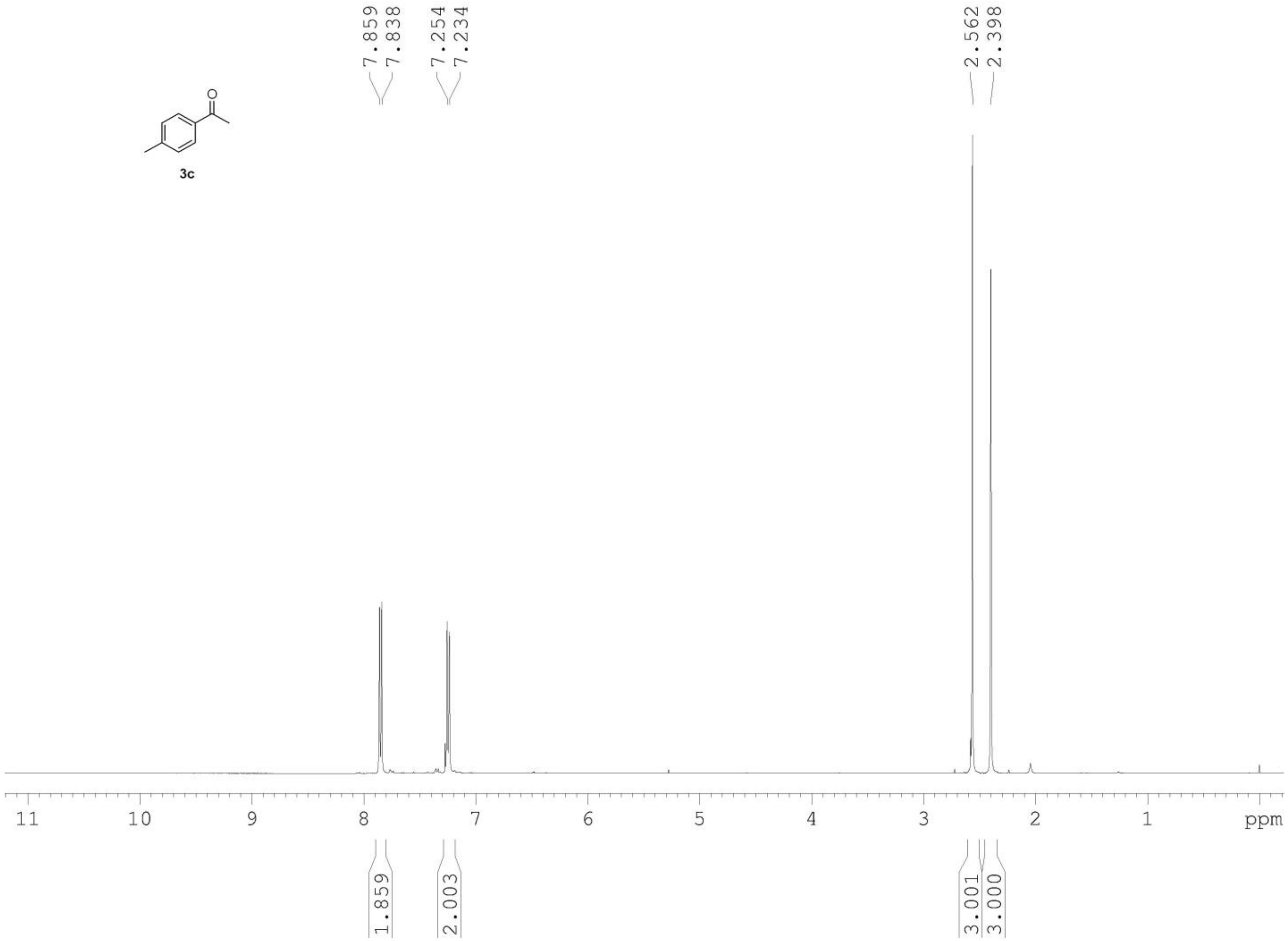

Figure S16. $^{13}C\{^1H\}$ NMR spectrum (100 MHz, $CDCl_3$) of **3c**

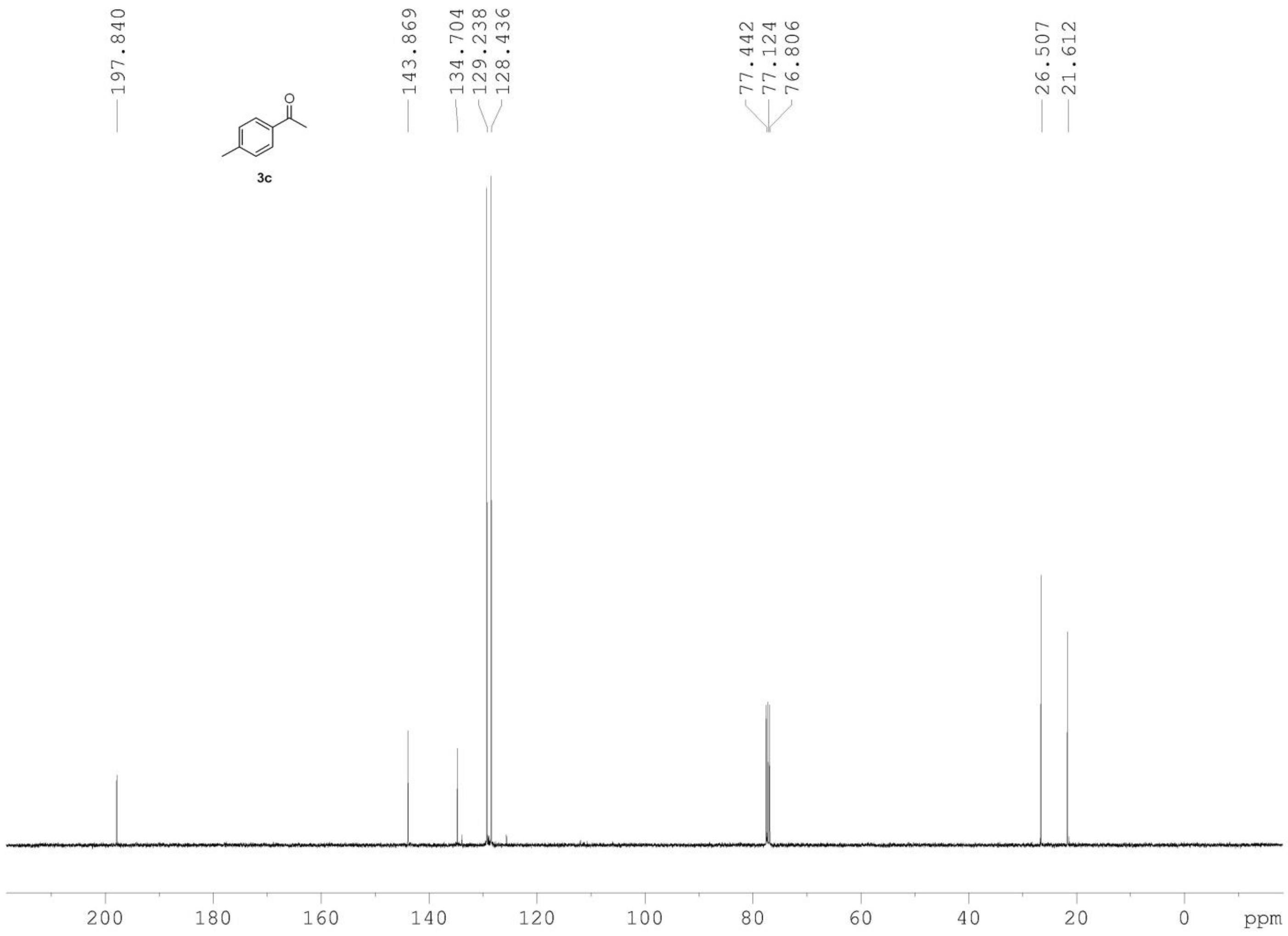

Figure S17. ^{1}H NMR spectrum (400 MHz, $CDCl_3$) of **3d**

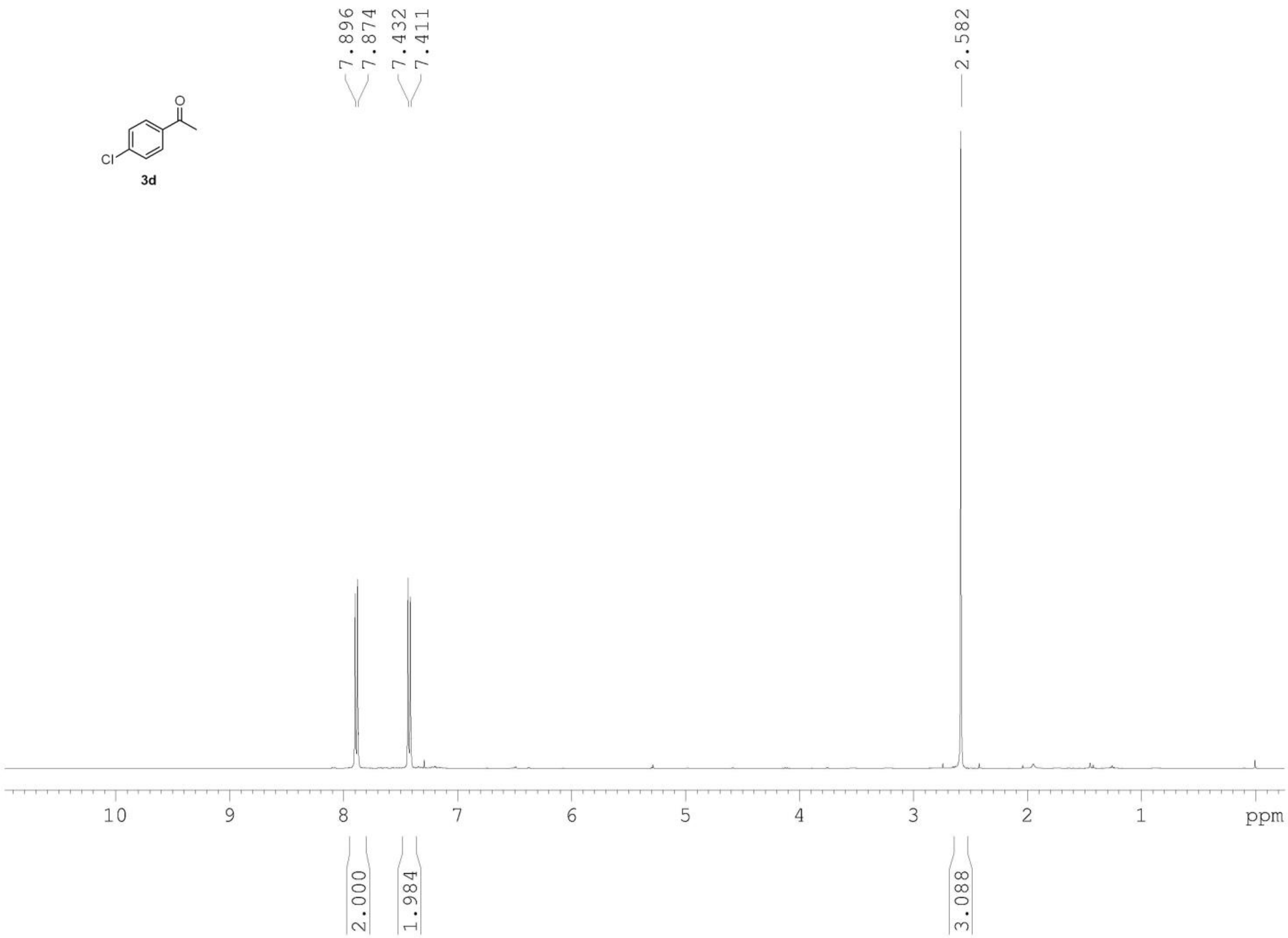

Figure S18. $^{13}C\{^1H\}$ NMR spectrum (100 MHz, $CDCl_3$) of **3d**

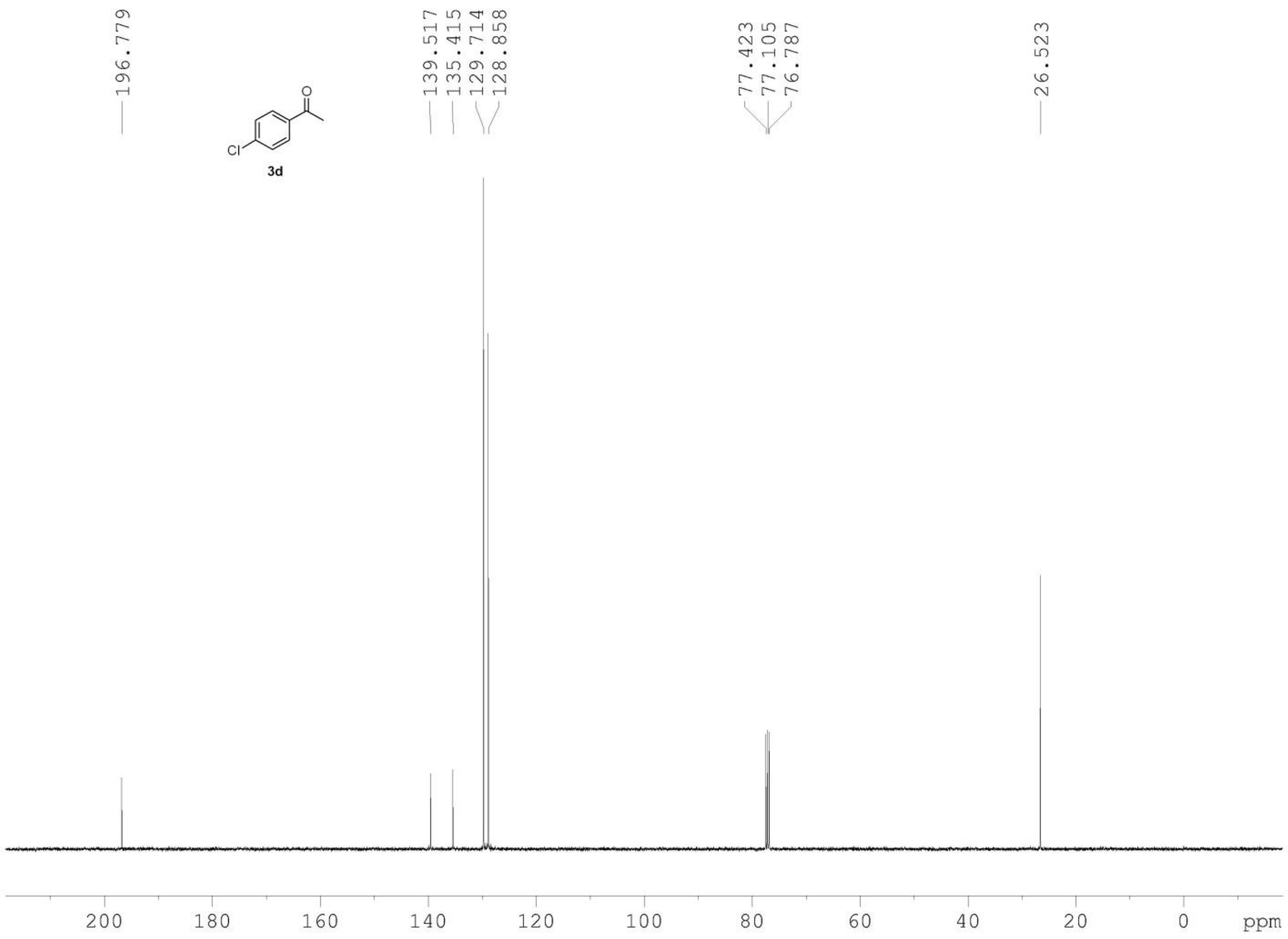

Figure S19. ^{1}H NMR spectrum (400 MHz, $CDCl_3$) of **3e**

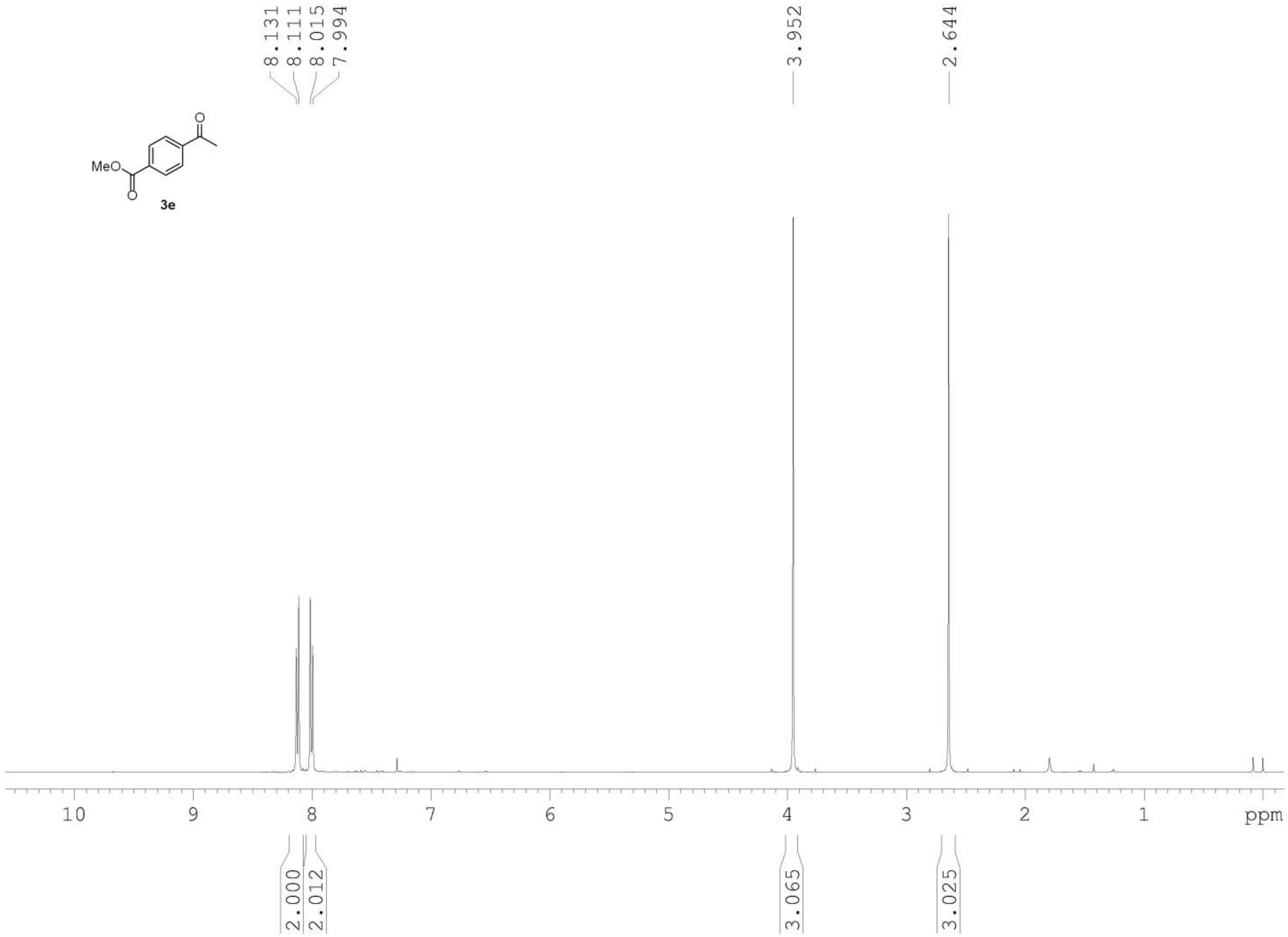

Figure S20. $^{13}C\{^1H\}$ NMR spectrum (100 MHz, $CDCl_3$) of **3e**

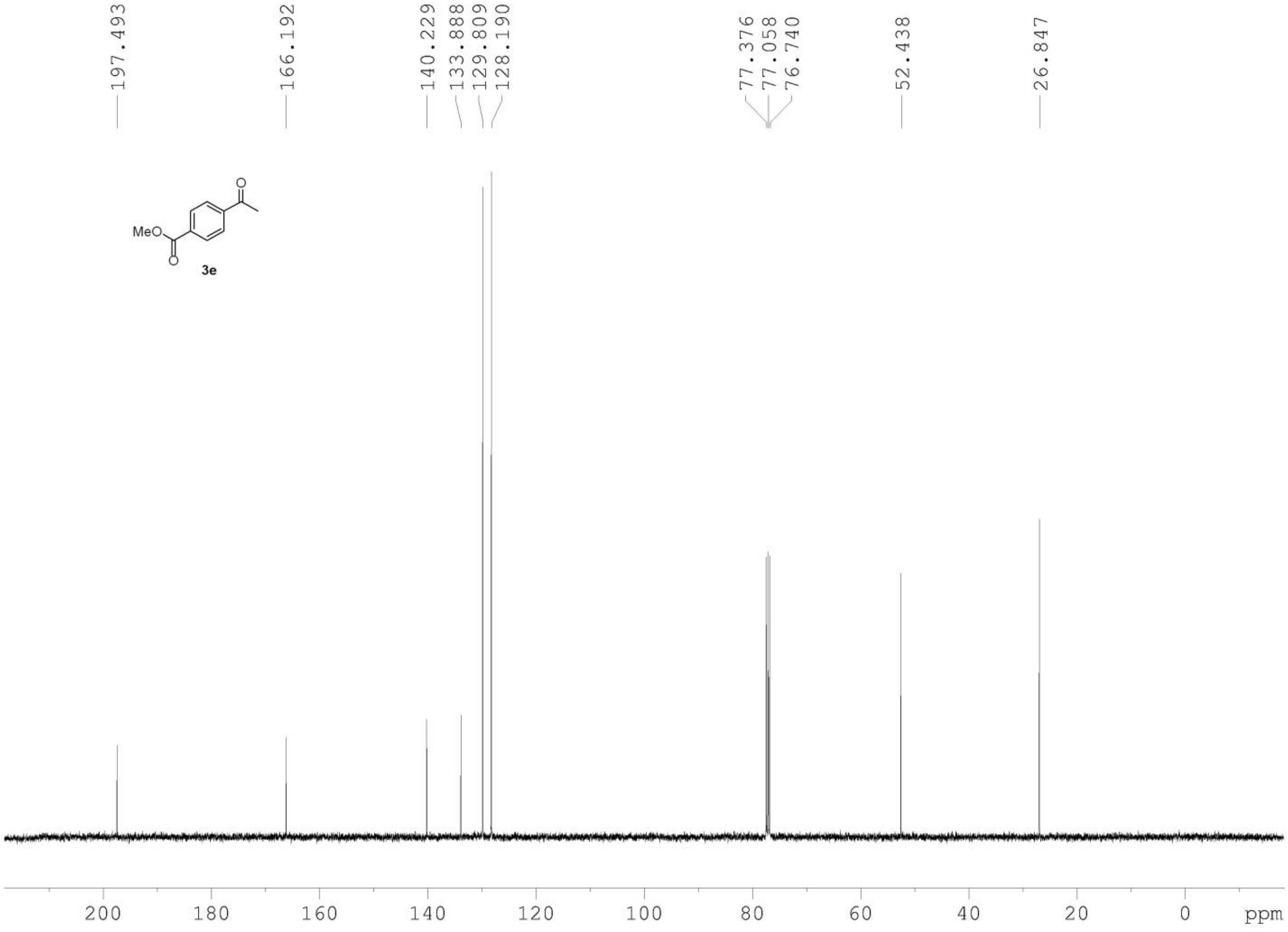

Figure S21. ^{1}H NMR spectrum (400 MHz, $CDCl_3$) of **3f**

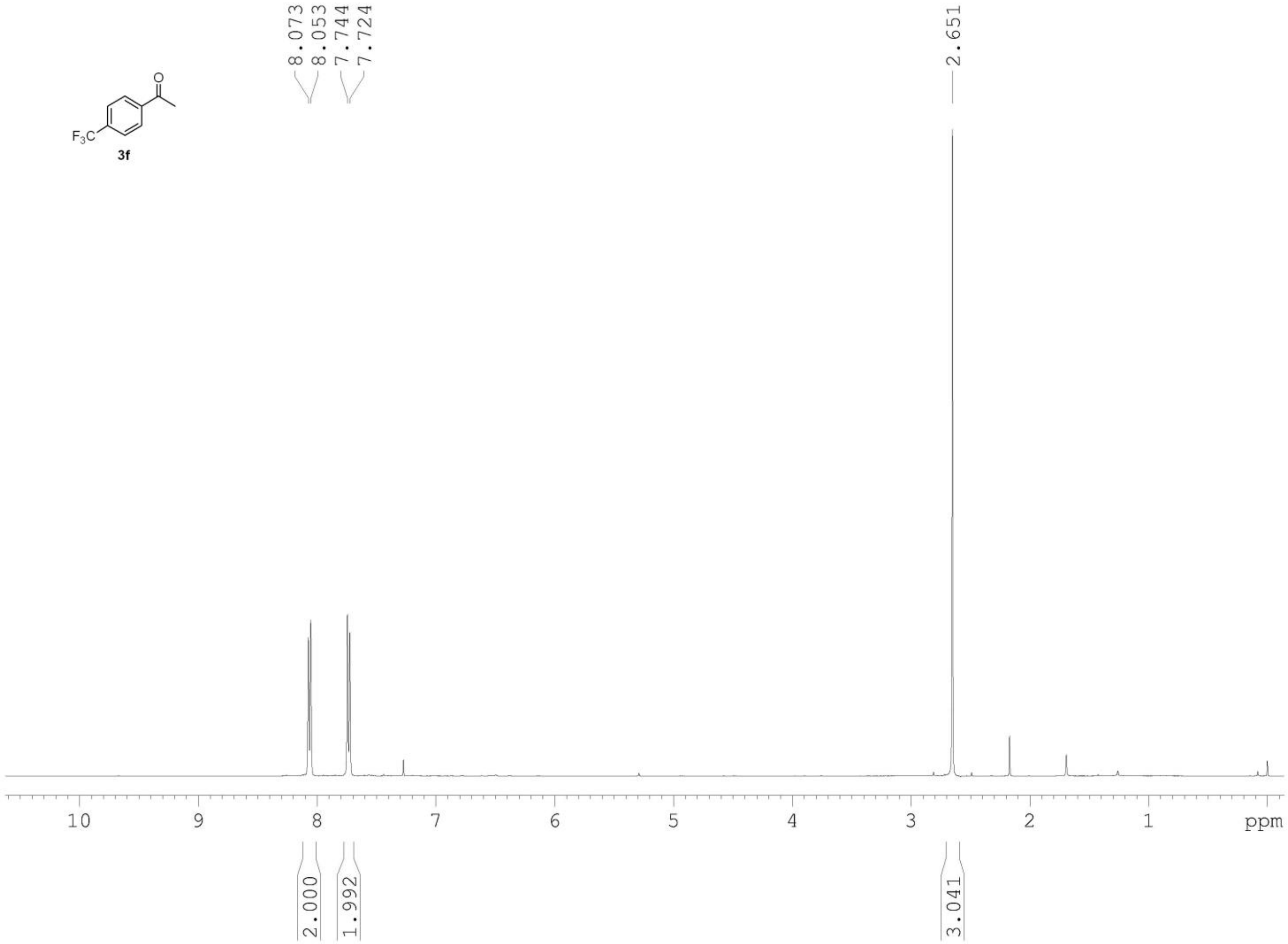

Figure S22. $^{13}C\{^{1}H\}$ NMR spectrum (100 MHz, $CDCl_3$) of **3f**

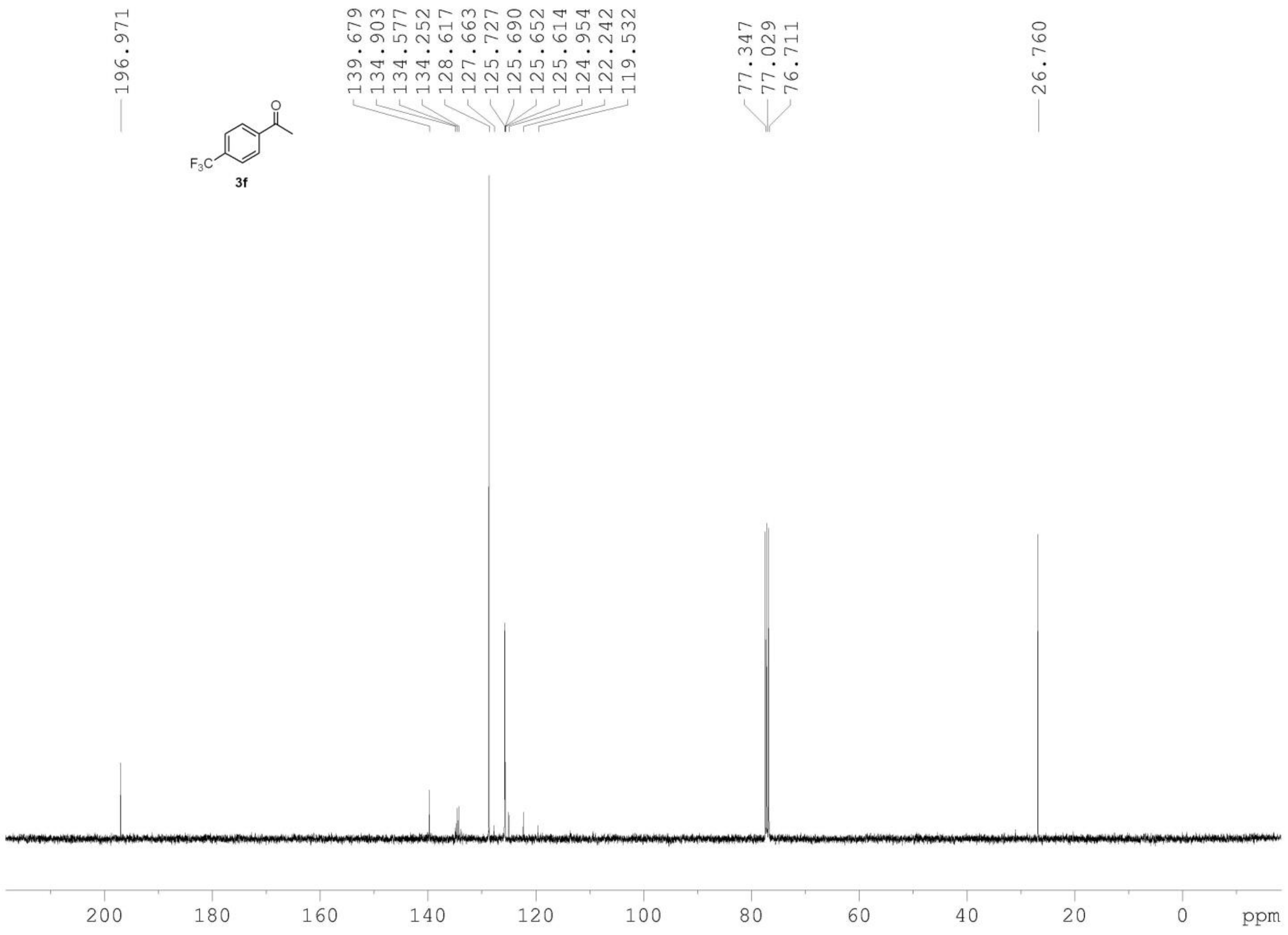

Figure S23. ^{1}H NMR spectrum (400 MHz, $CDCl_3$) of **3g**

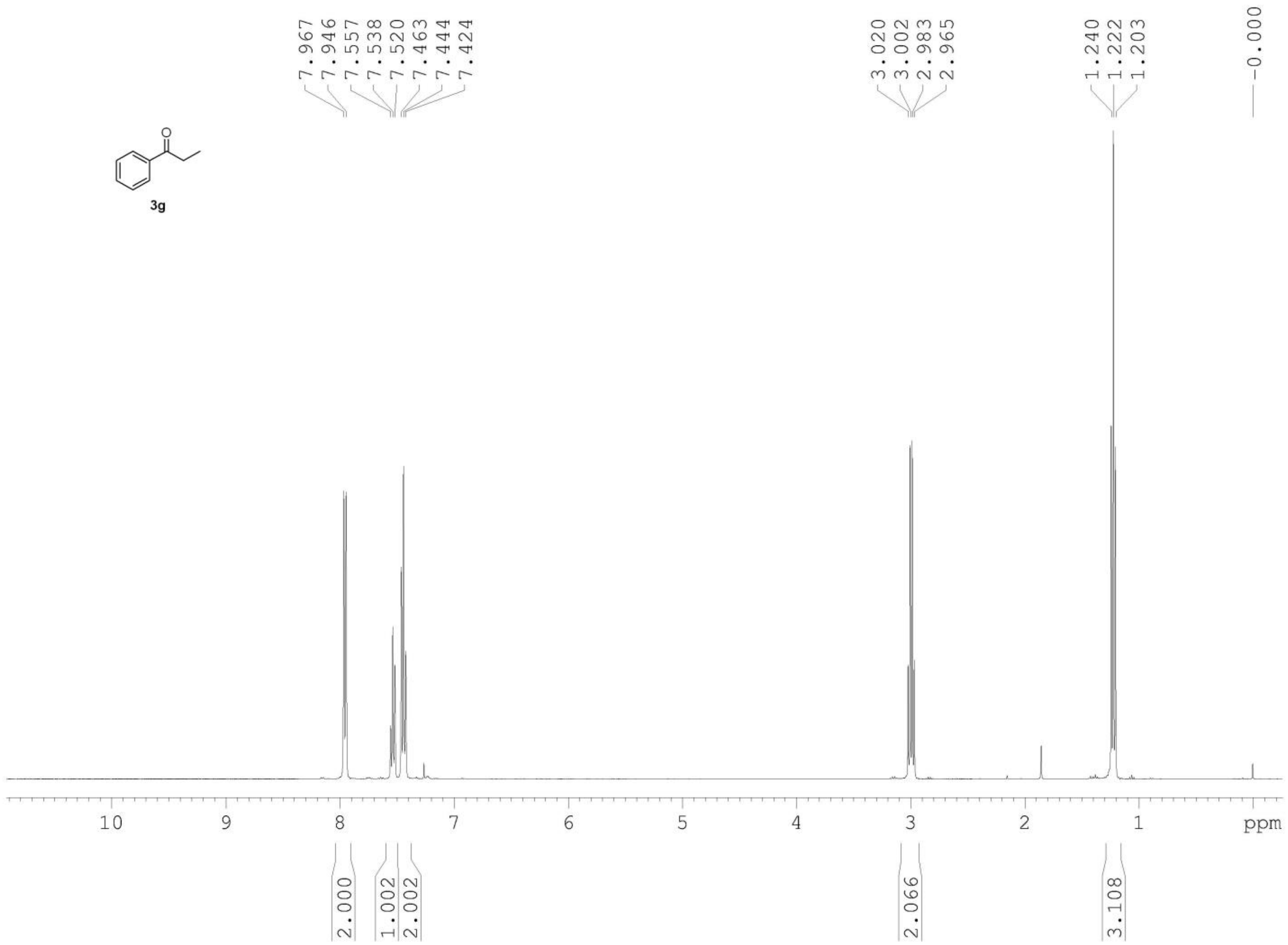

Figure S24. $^{13}C\{^{1}H\}$ NMR spectrum (100 MHz, $CDCl_3$) of **3g**

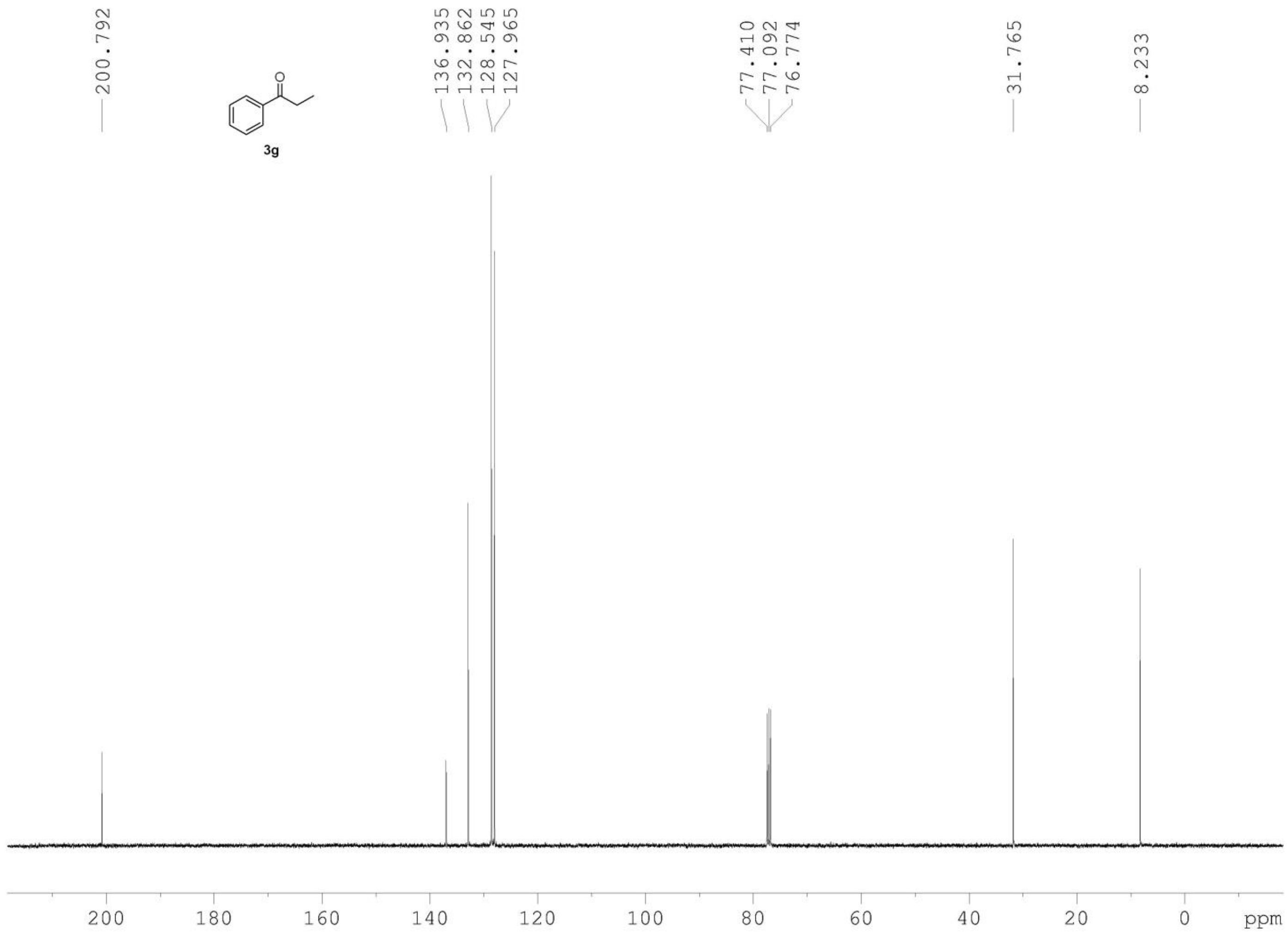

Figure S25. ^{1}H NMR spectrum (400 MHz, $CDCl_3$) of **3h**

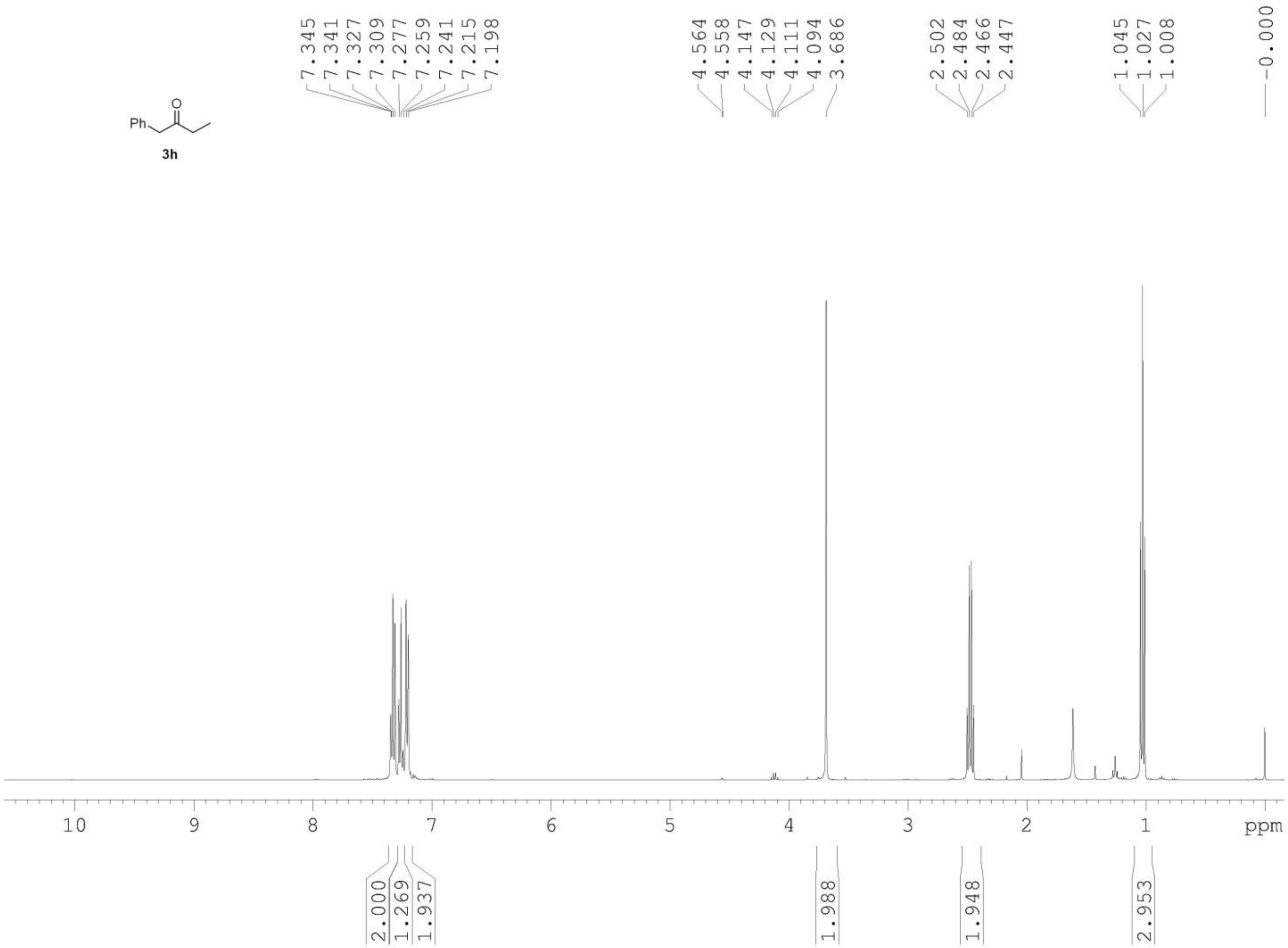

Figure S26. $^{13}C\{^{1}H\}$ NMR spectrum (100 MHz, $CDCl_3$) of **3h**

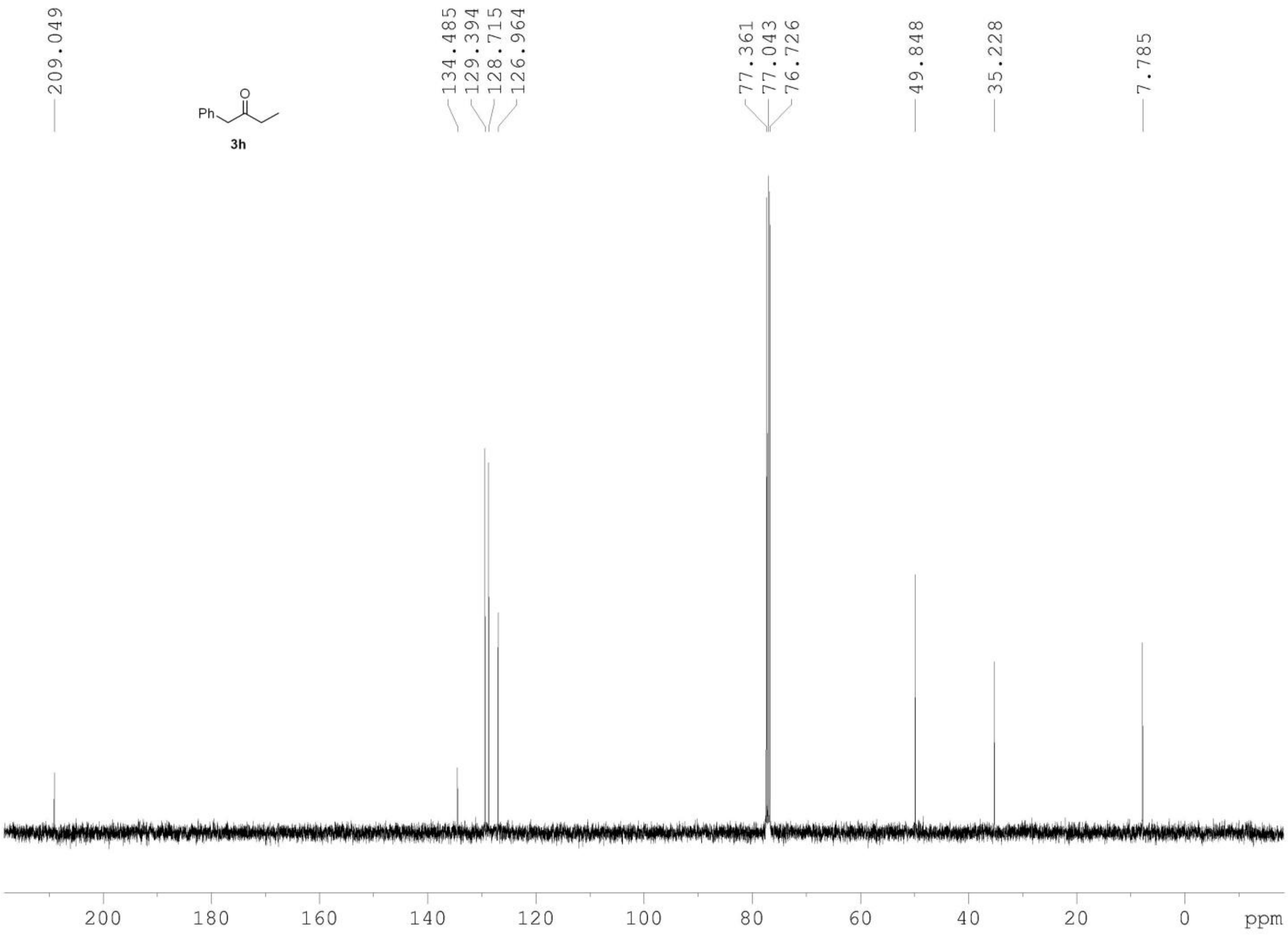

Figure S27. ^{1}H NMR spectrum (400 MHz, $CDCl_3$) of **3j**

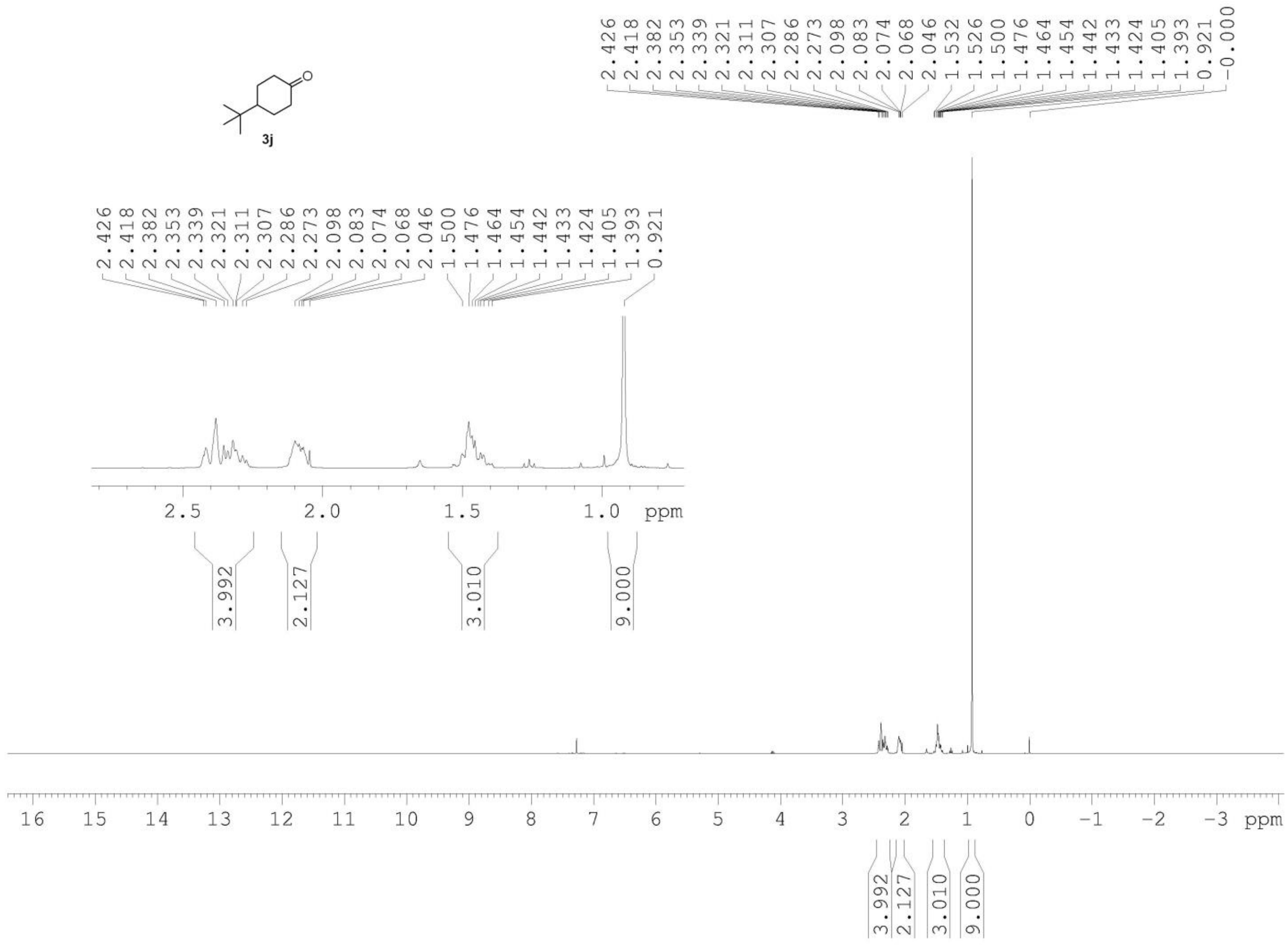

Figure S28. $^{13}C\{^{1}H\}$ NMR spectrum (100 MHz, $CDCl_3$) of **3j**

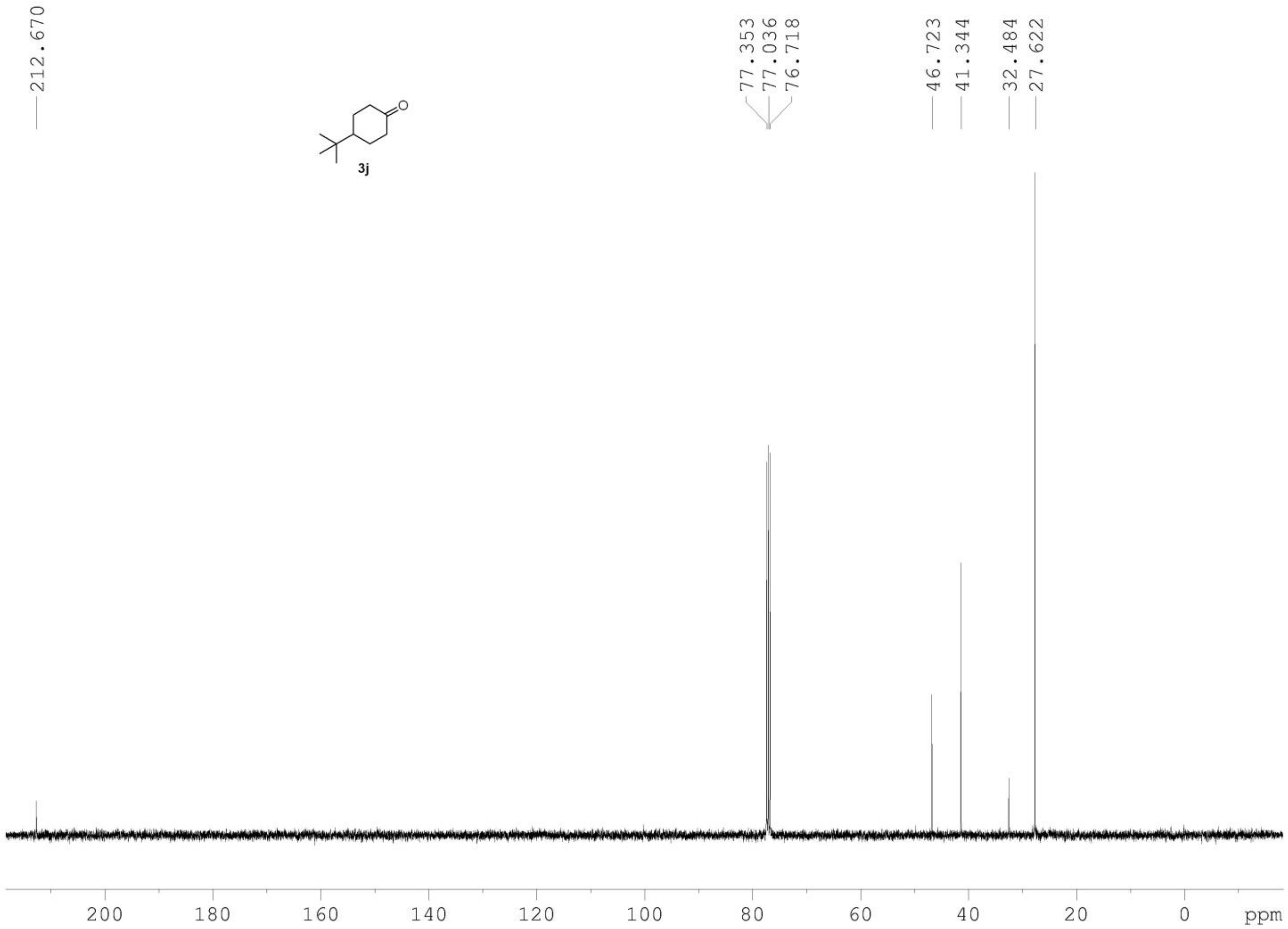

Figure S29. ^{1}H NMR spectrum (400 MHz, $CDCl_3$) of **3k**

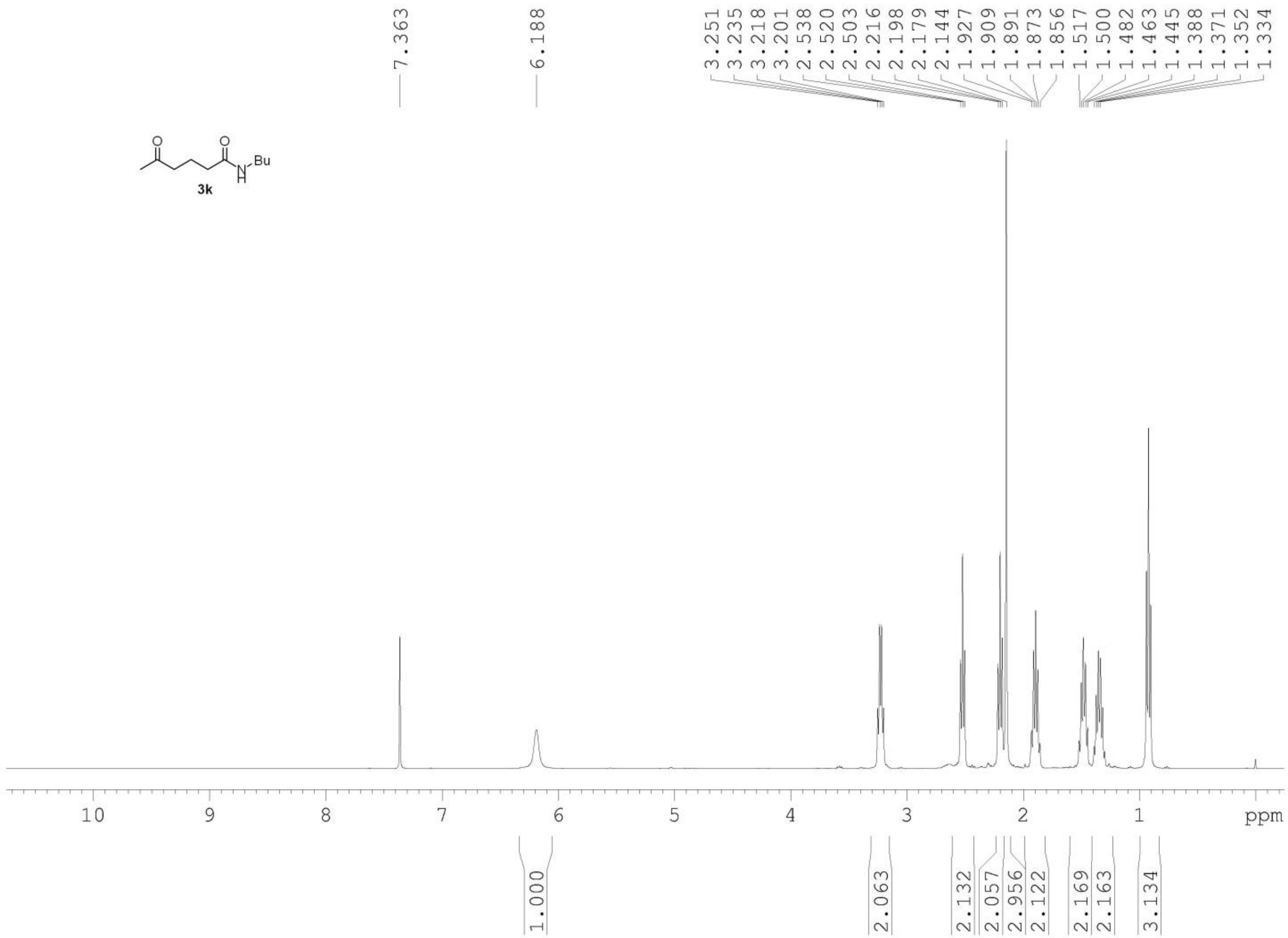

Figure S30. $^{13}C\{^1H\}$ NMR spectrum (100 MHz, $CDCl_3$) of **3k**

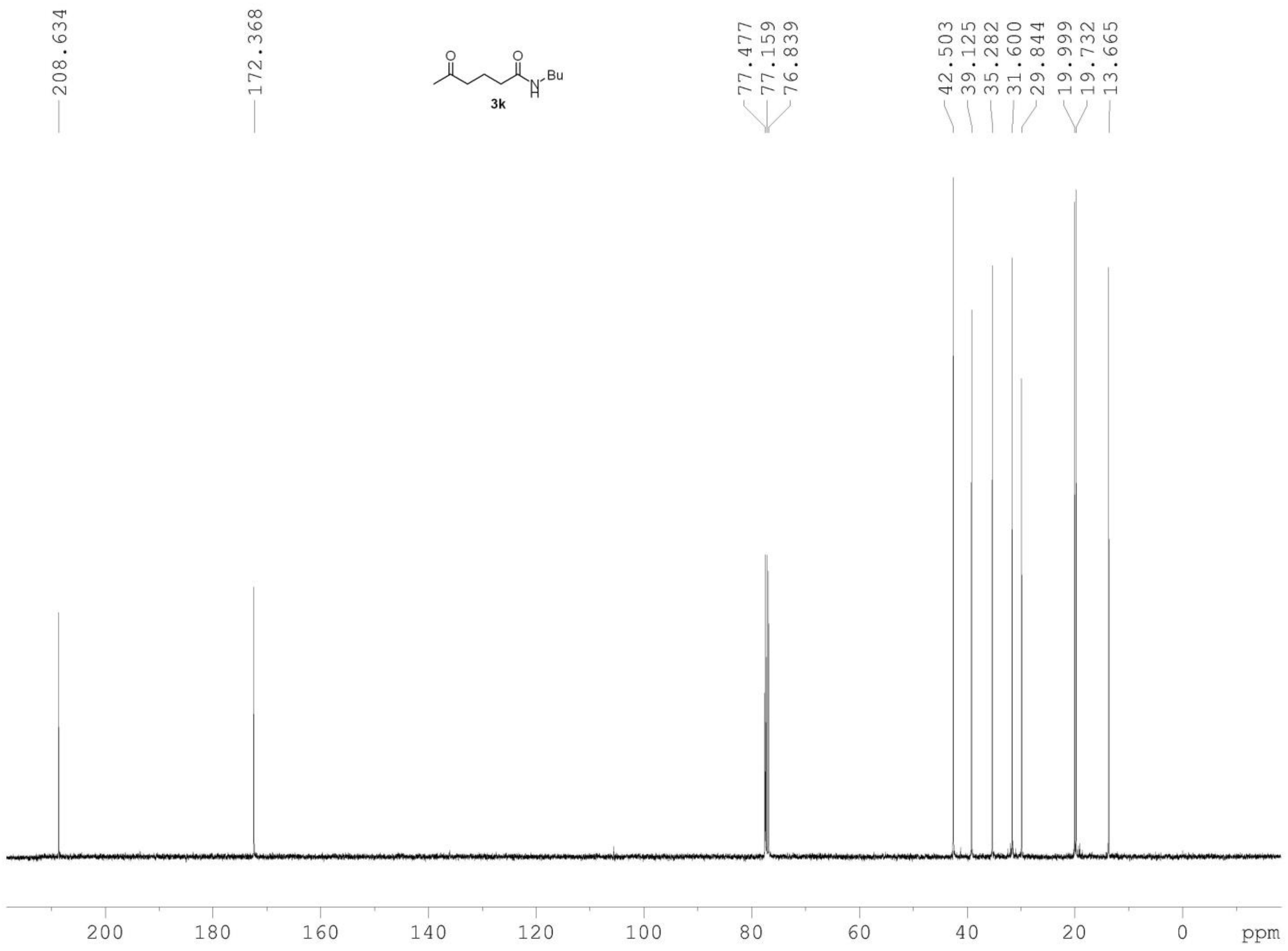

Figure S31. ^{1}H NMR spectrum (400 MHz, $CDCl_3$) of **3m**

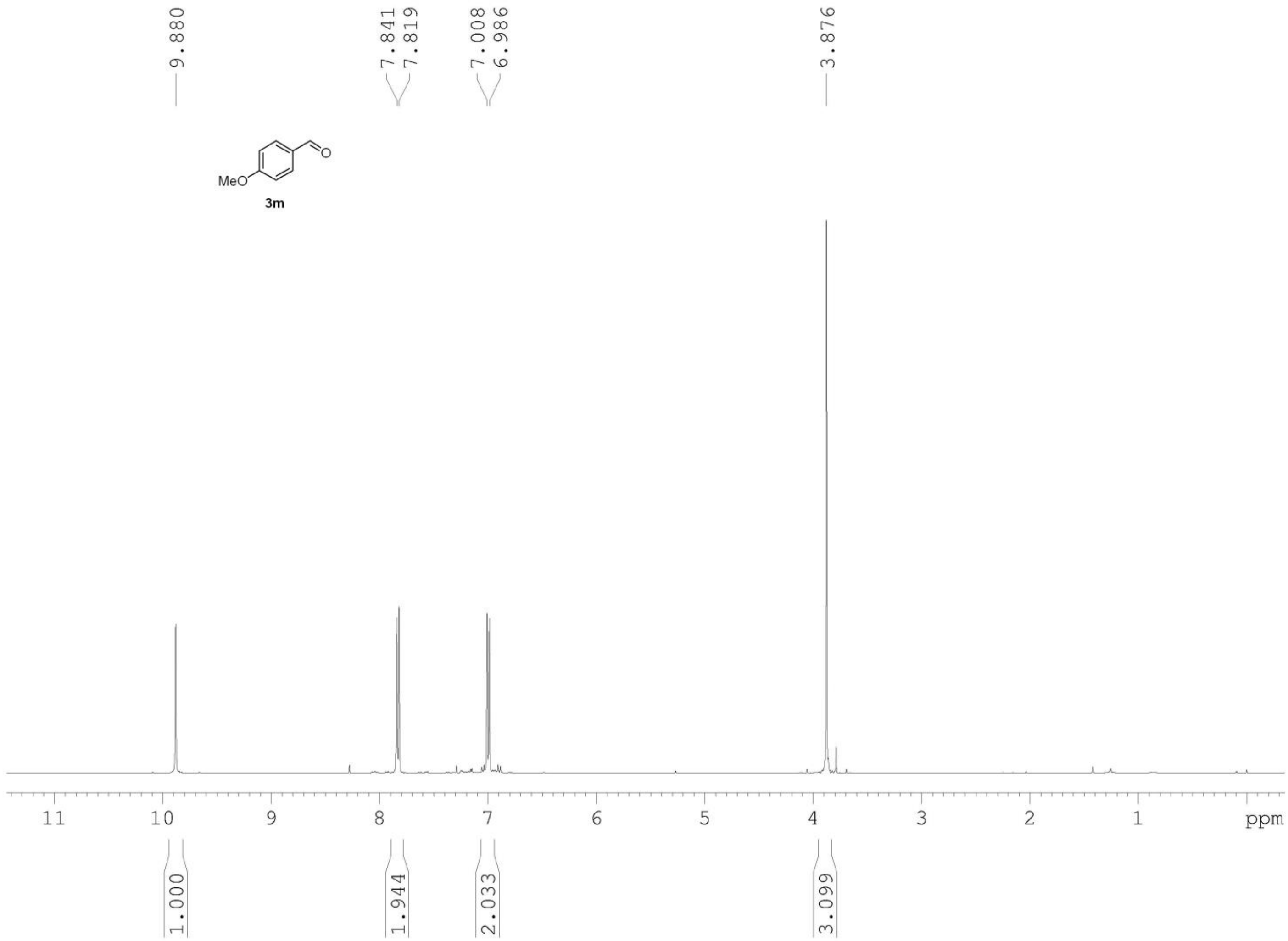

Figure S32. $^{13}C\{^1H\}$ NMR spectrum (100 MHz, $CDCl_3$) of **3m**

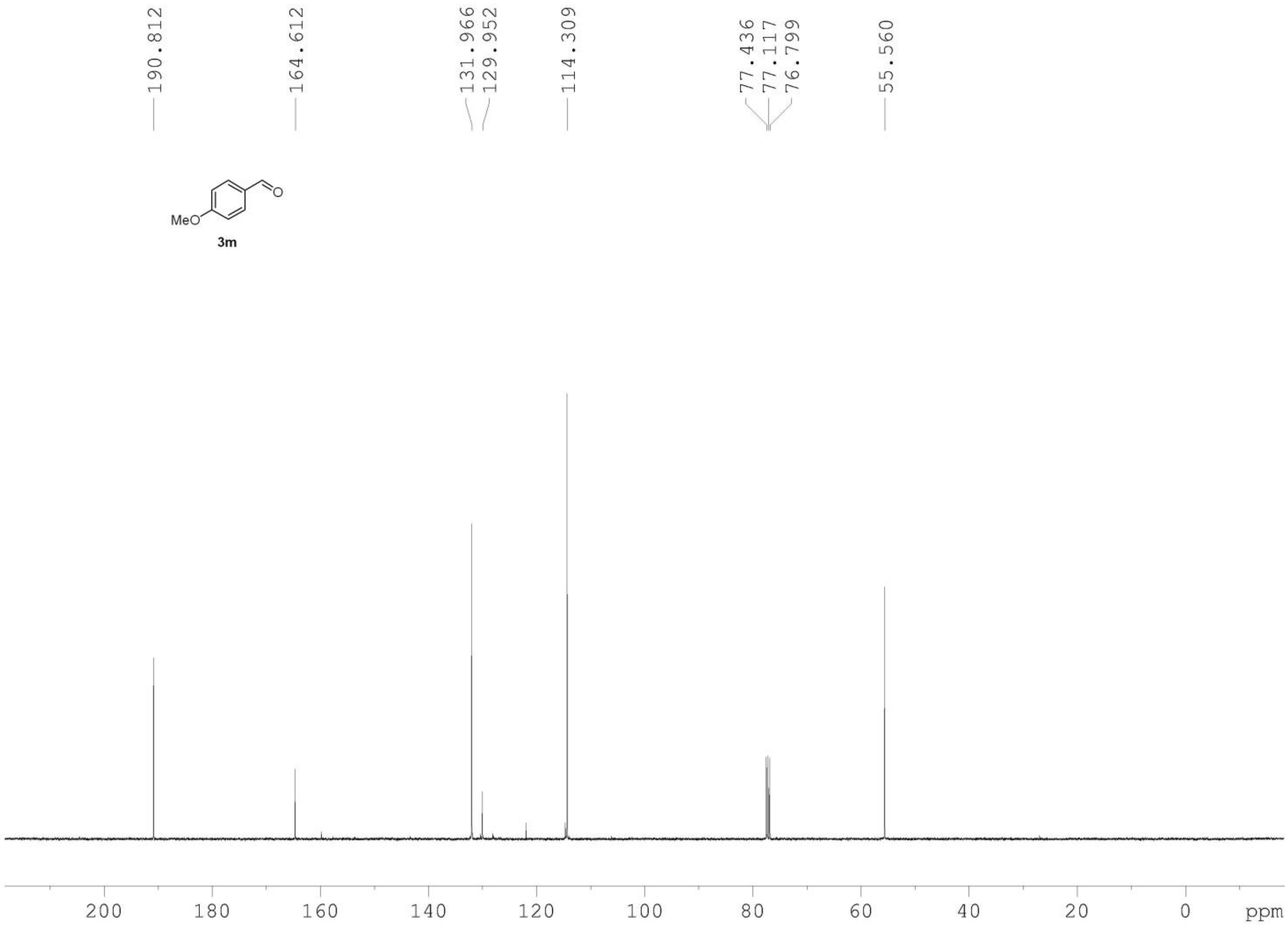

Figure S33. ^{1}H NMR spectrum (400 MHz, $CDCl_3$) of **3n**

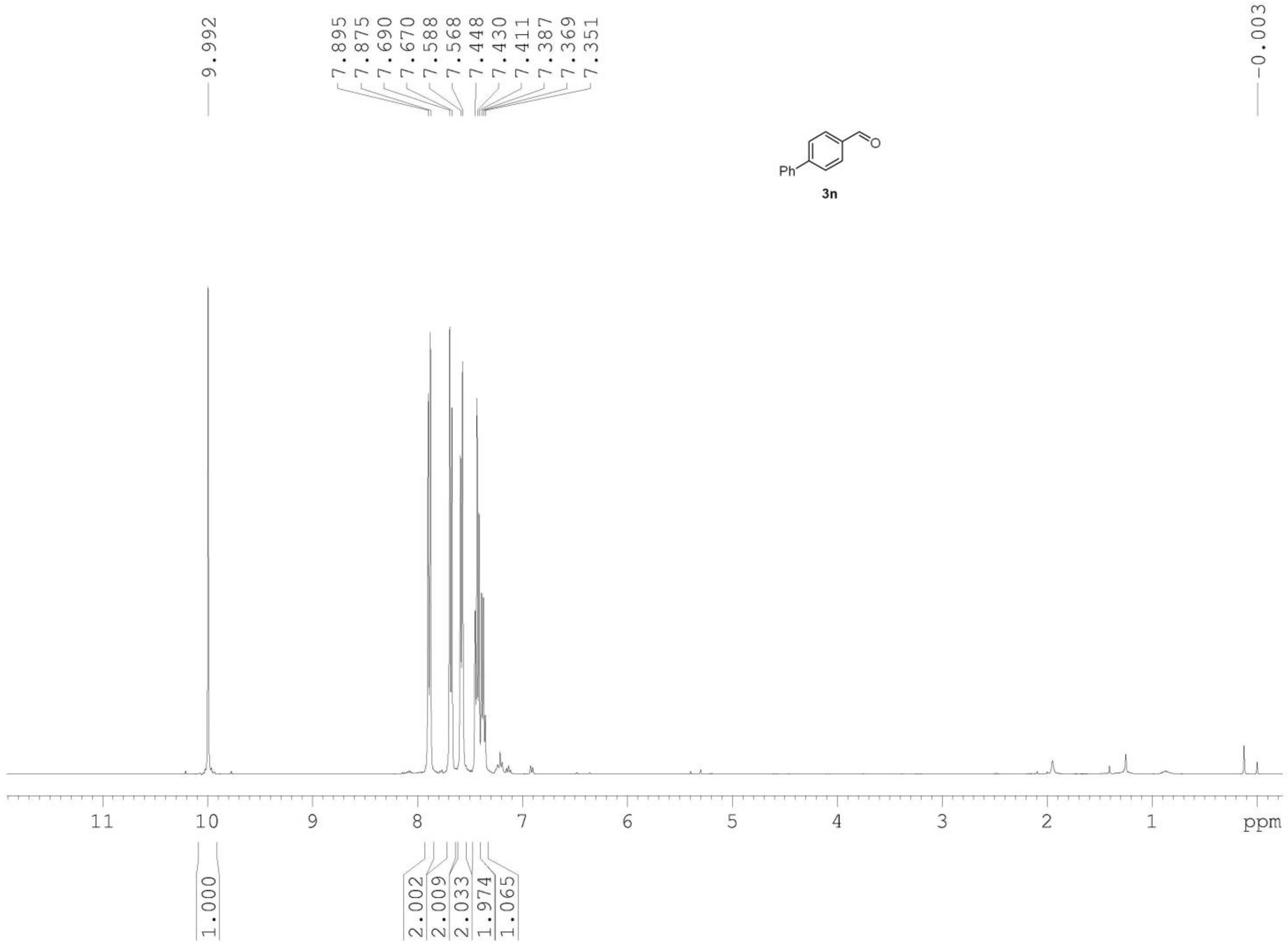

Figure S34. $^{13}C\{^{1}H\}$ NMR spectrum (100 MHz, $CDCl_3$) of **3n**

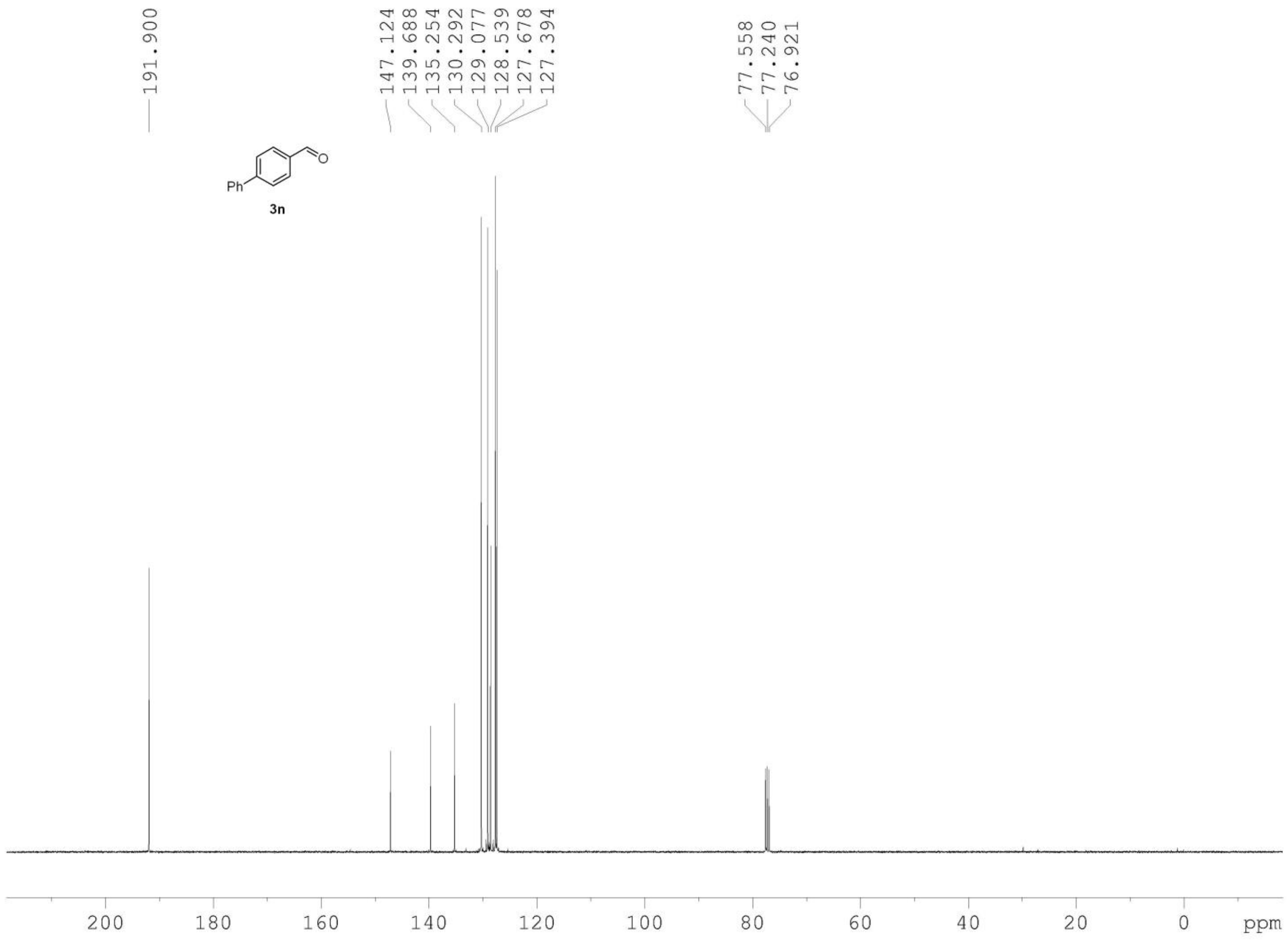

Figure S35. ^{1}H NMR spectrum (400 MHz, $CDCl_3$) of **3o**

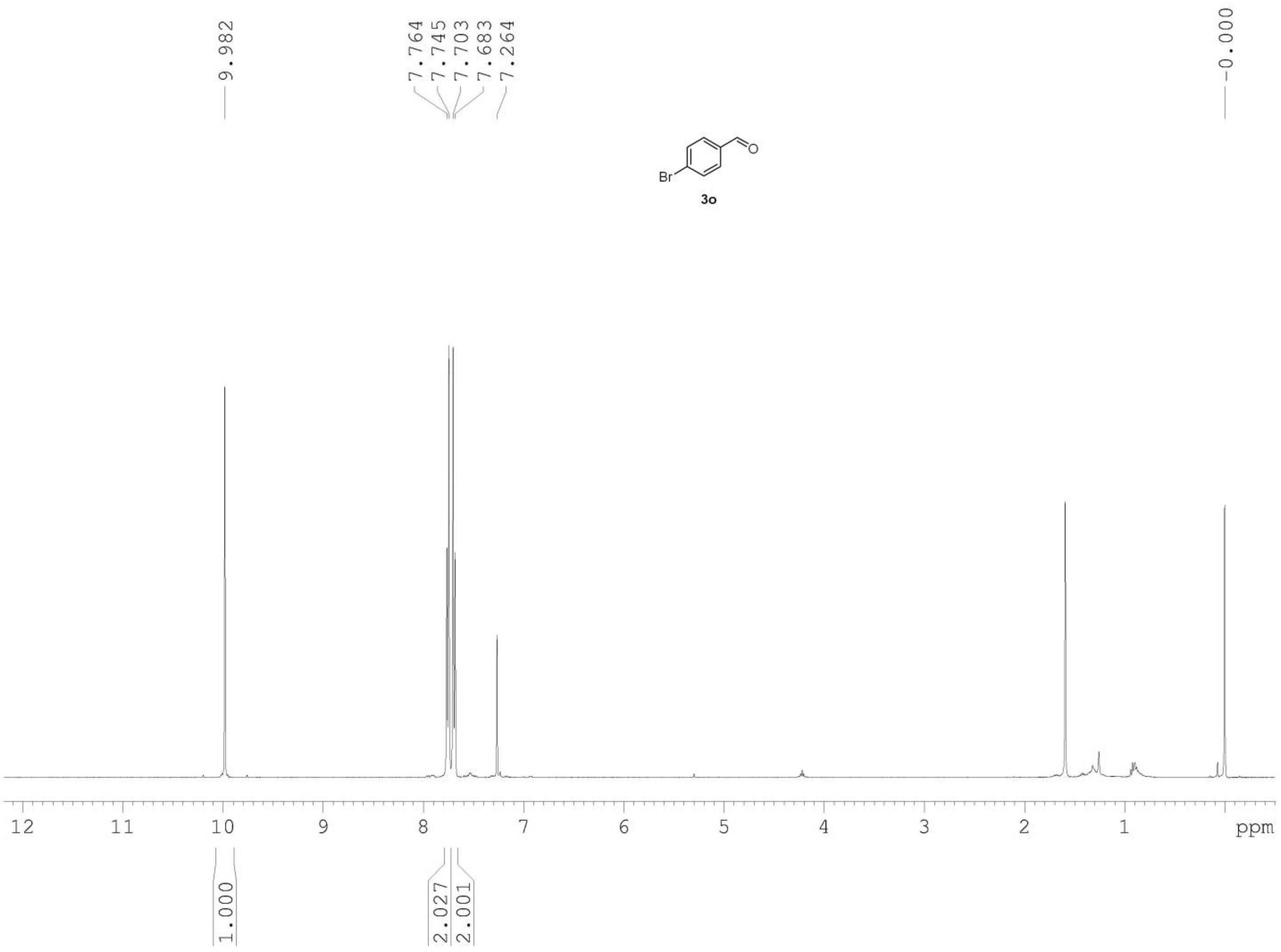

Figure S36. $^{13}C\{^{1}H\}$ NMR spectrum (100 MHz, $CDCl_3$) of **3o**

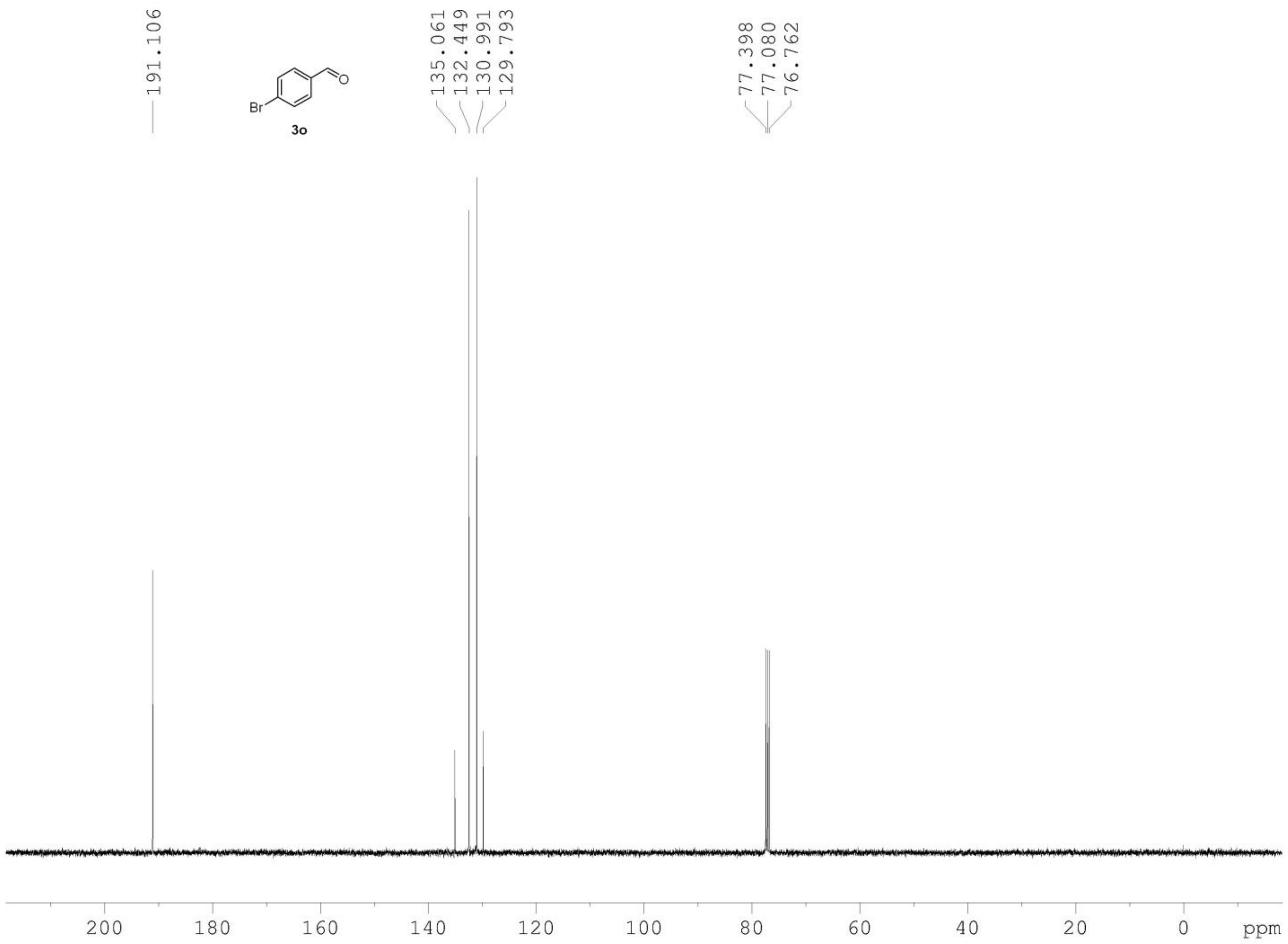

Figure S37. ^{1}H NMR spectrum (400 MHz, $CDCl_3$) of **3p**

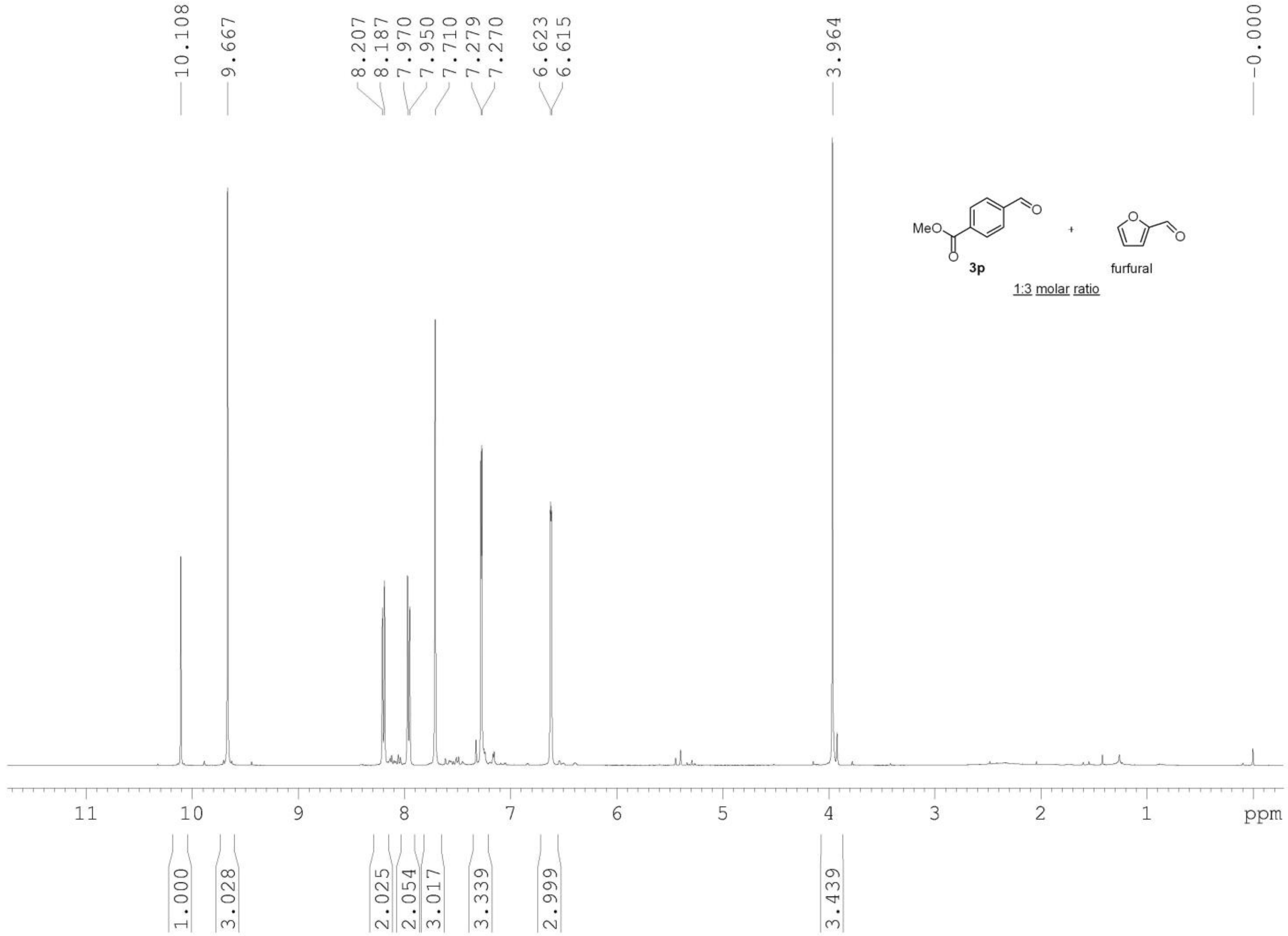

Figure S38. $^{13}C\{^{1}H\}$ NMR spectrum (100 MHz, $CDCl_3$) of **3p**

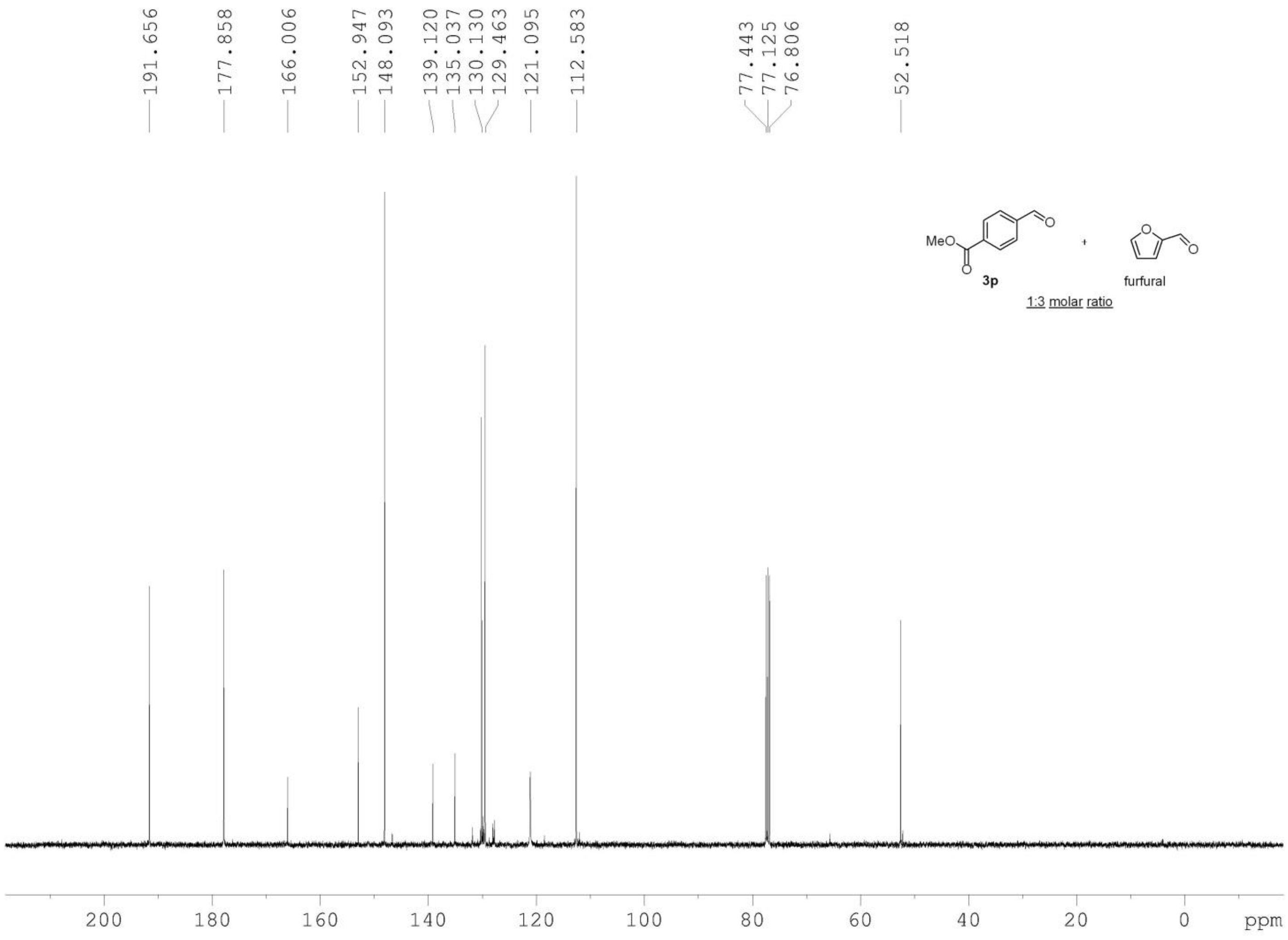

Figure S39. ^1H NMR spectrum (400 MHz, $CDCl_3$) of **3q**

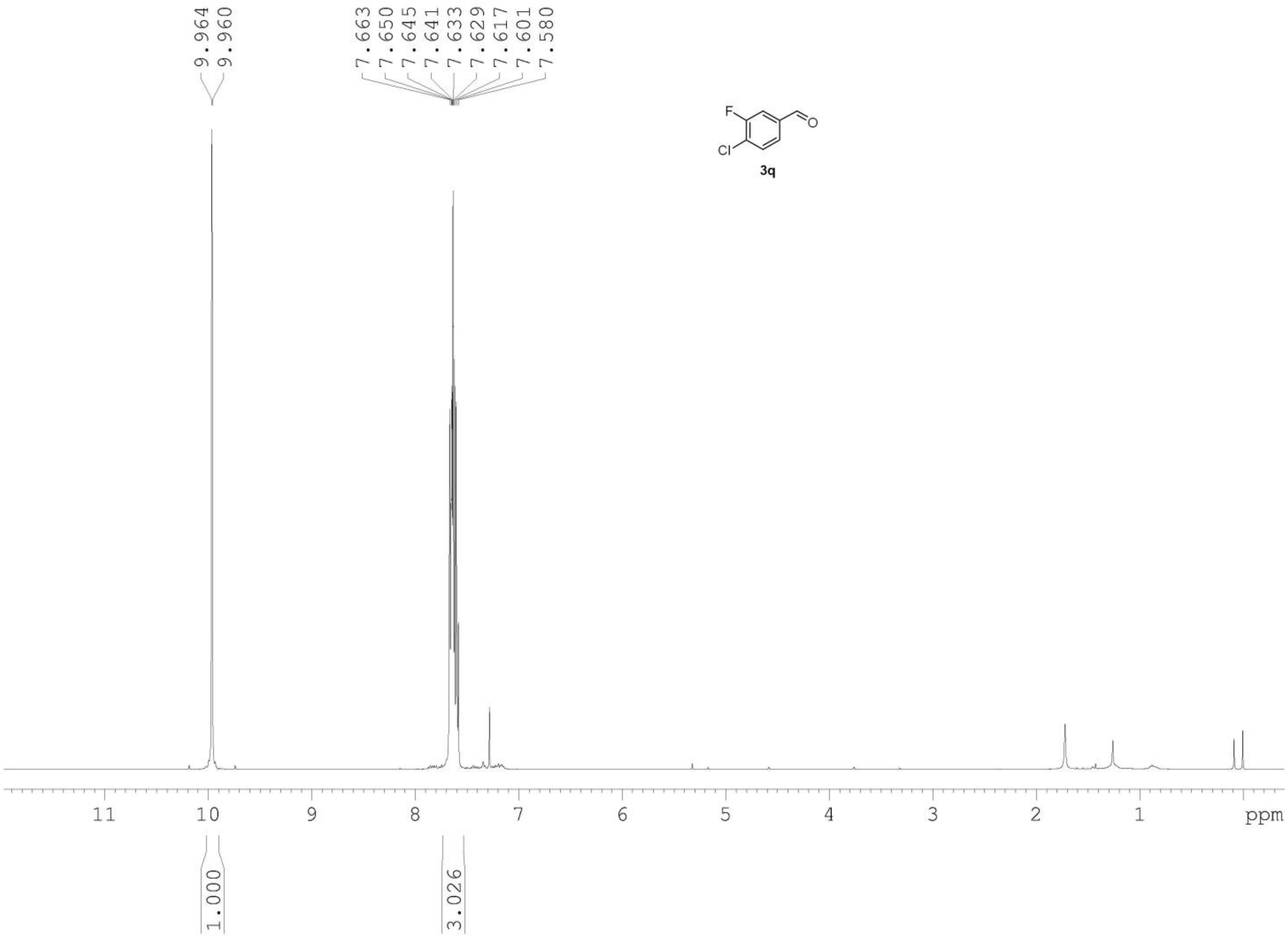

Figure S40. $^{13}C\{^1H\}$ NMR spectrum (100 MHz, $CDCl_3$) of **3q**

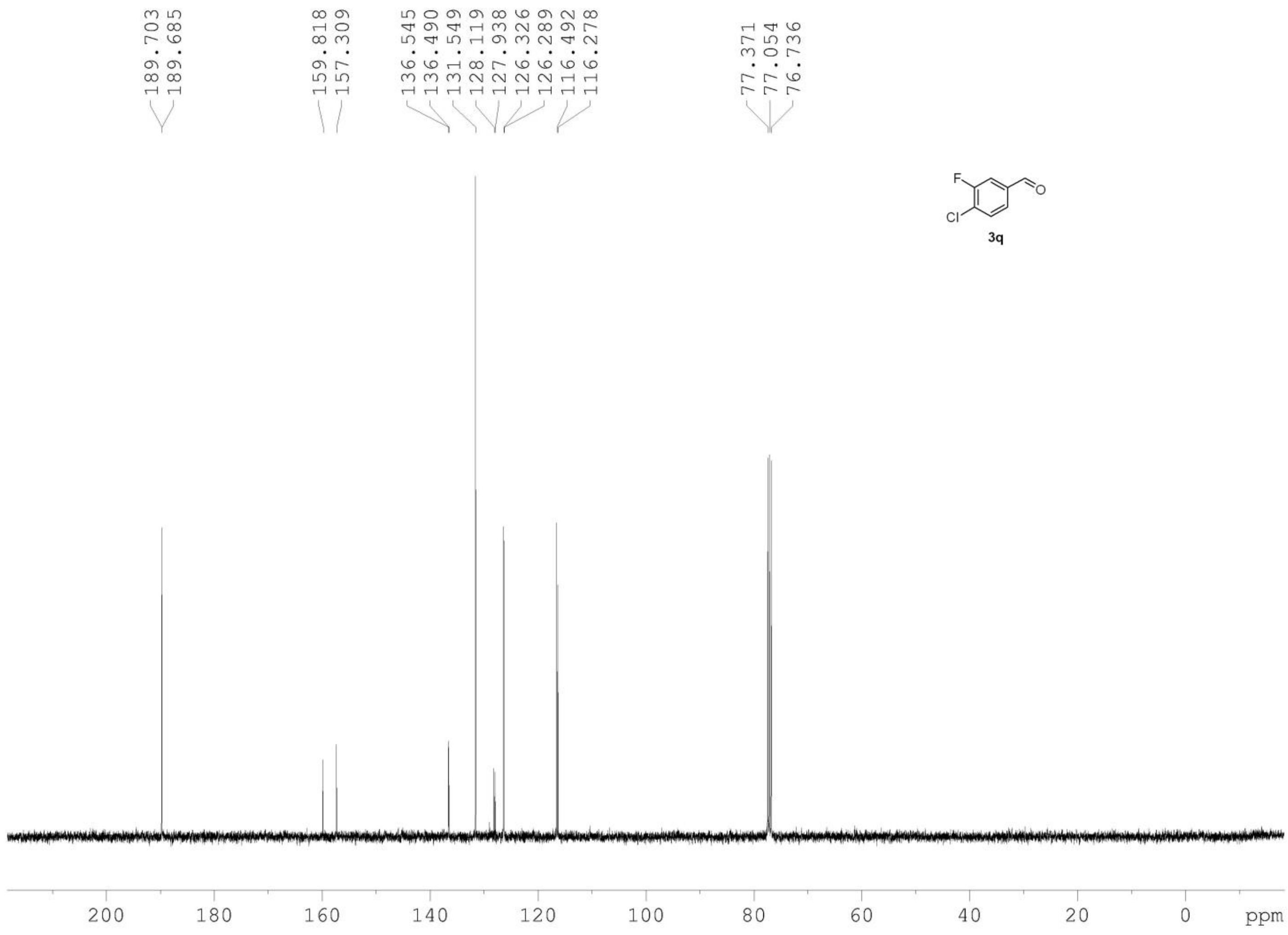

Figure S41. ^{1}H NMR spectrum (400 MHz, $CDCl_3$) of **3s**

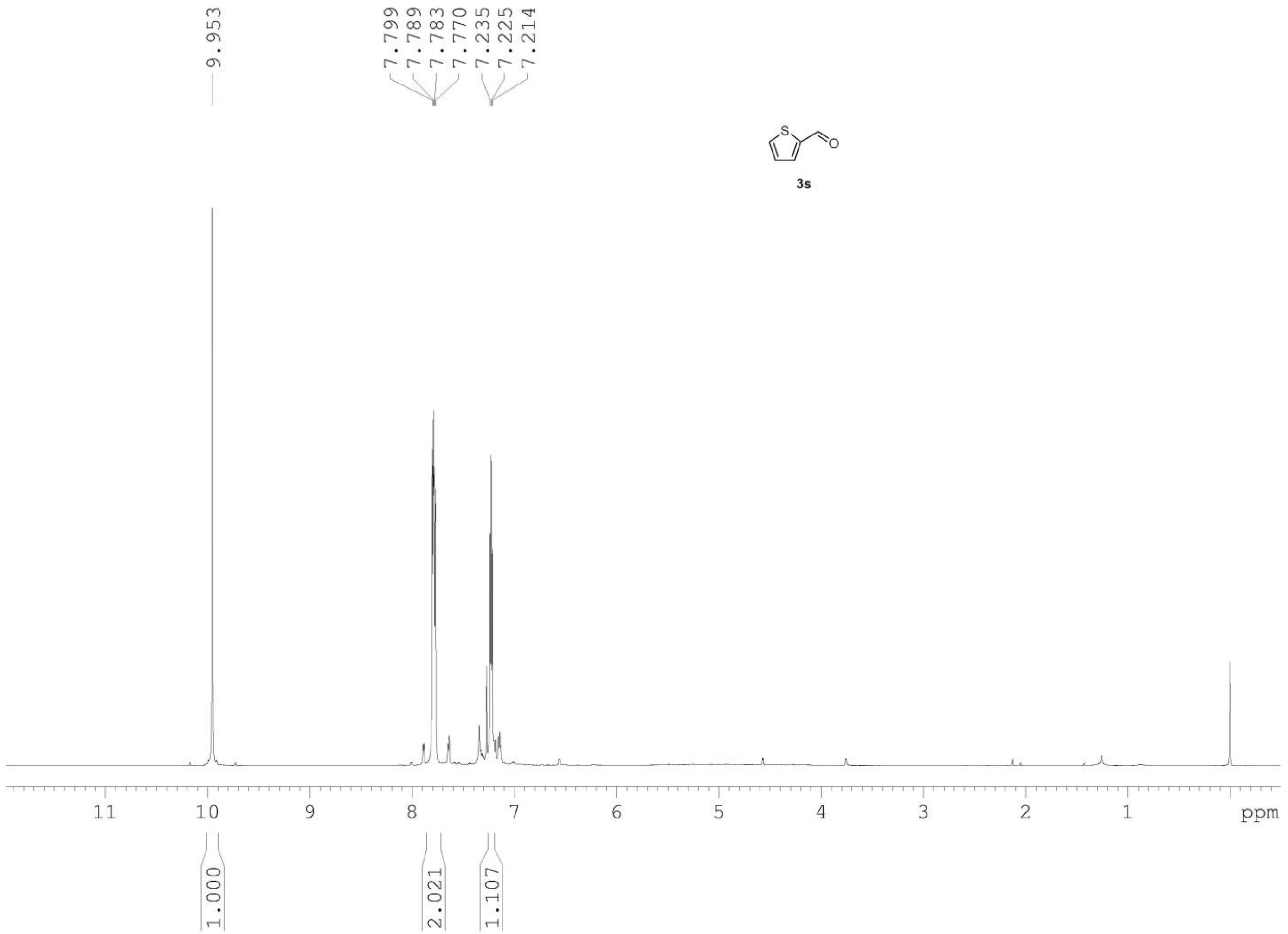

Figure S42. $^{13}C\{^{1}H\}$ NMR spectrum (100 MHz, $CDCl_3$) of **3s**

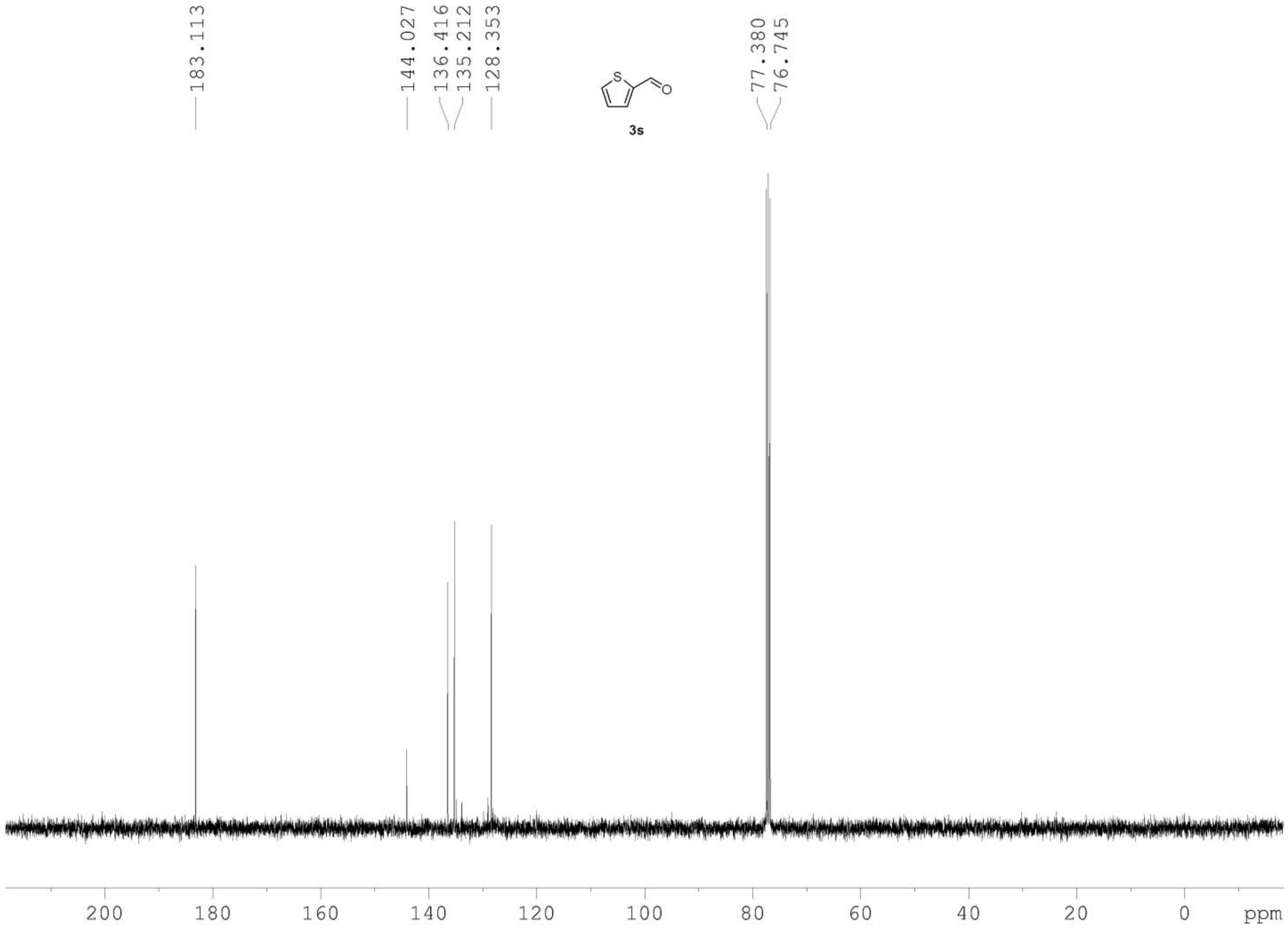

Figure S43. ^{1}H NMR spectrum (400 MHz, $CDCl_3$) of **3t**

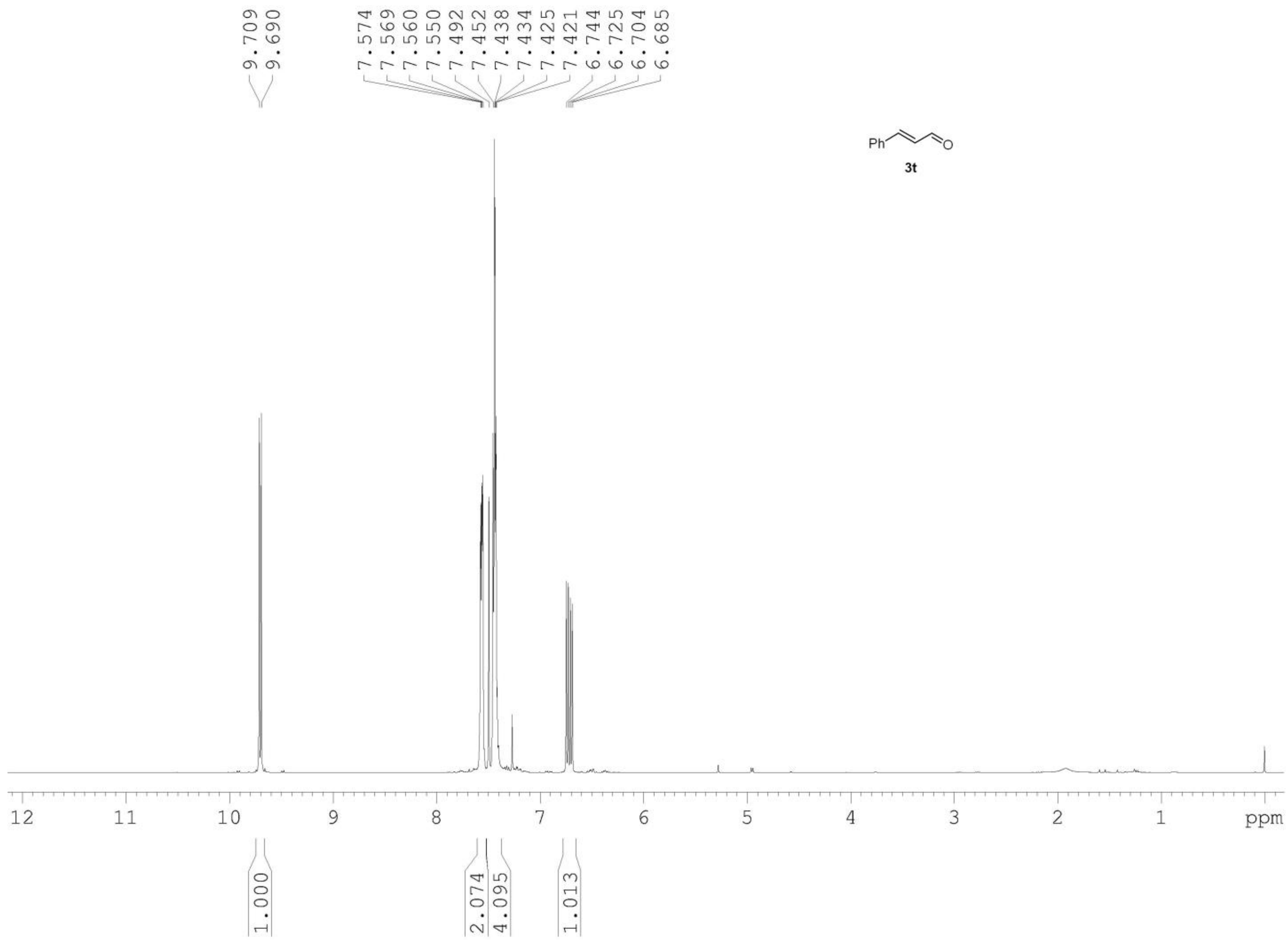

Figure S44. $^{13}C\{^{1}H\}$ NMR spectrum (100 MHz, $CDCl_3$) of **3t**

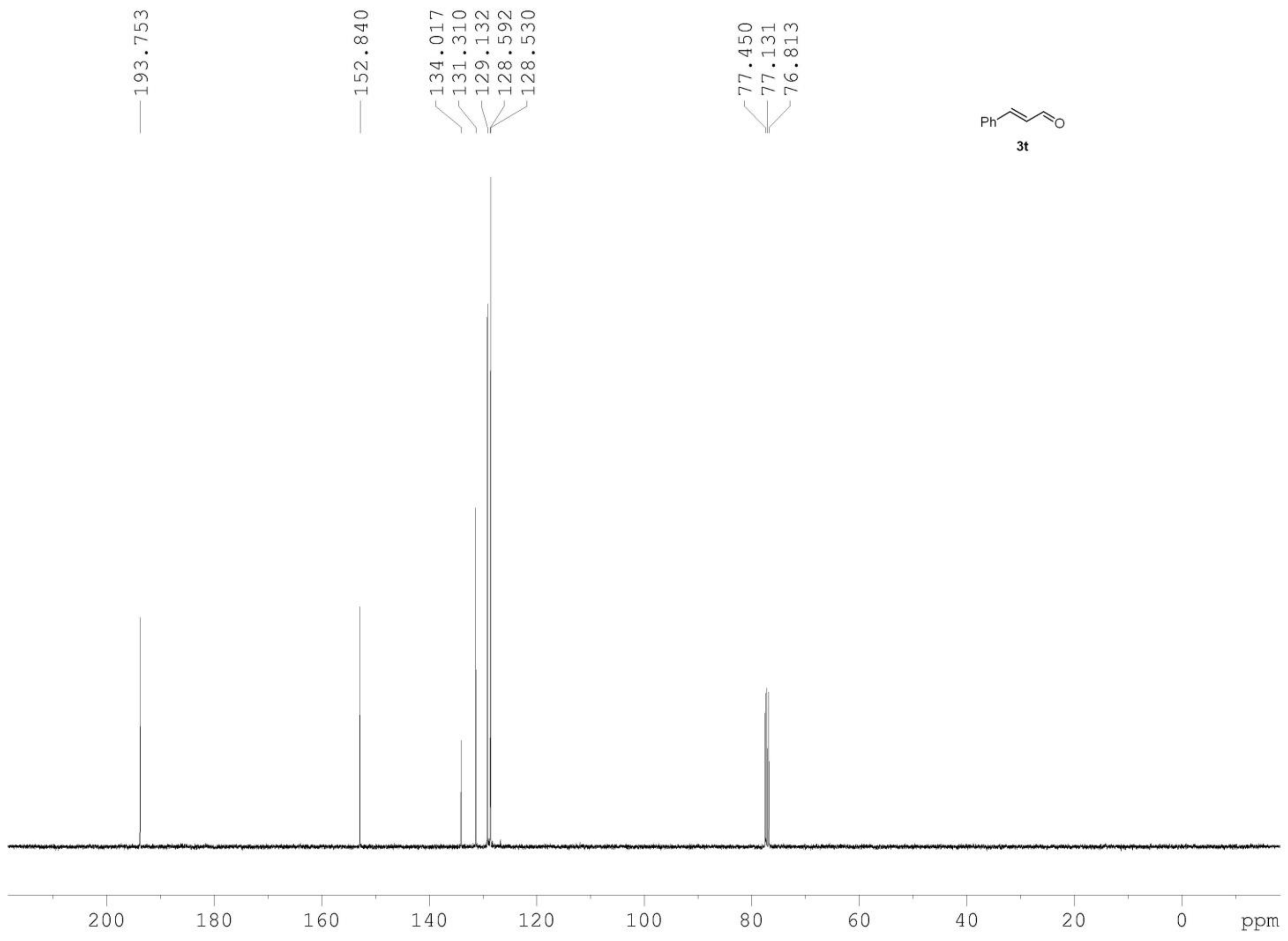

Figure S45. ^{1}H NMR spectrum (400 MHz, $CDCl_3$) of **3u**

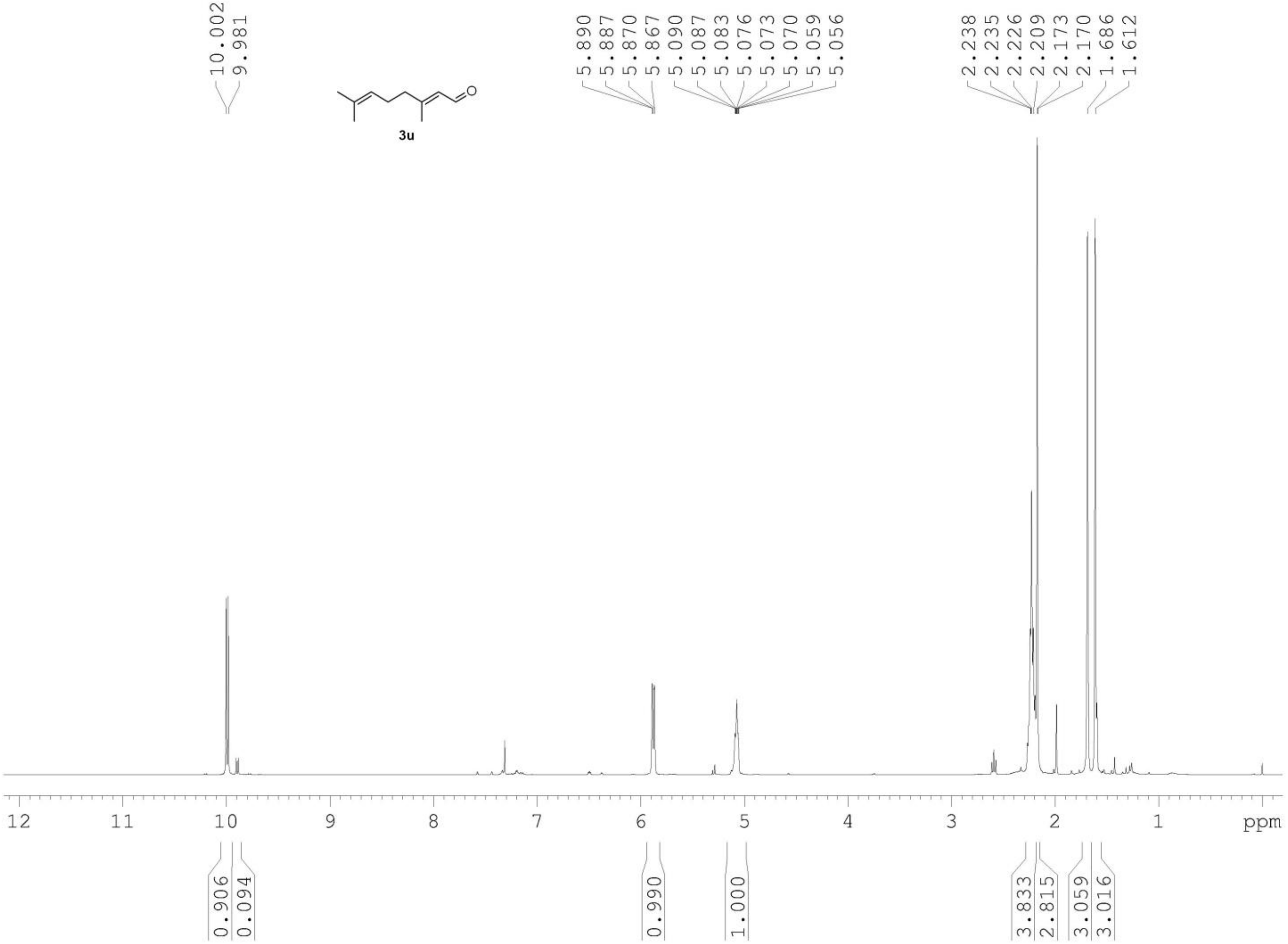

Figure S46. $^{13}C\{^1H\}$ NMR spectrum (100 MHz, $CDCl_3$) of **3u**

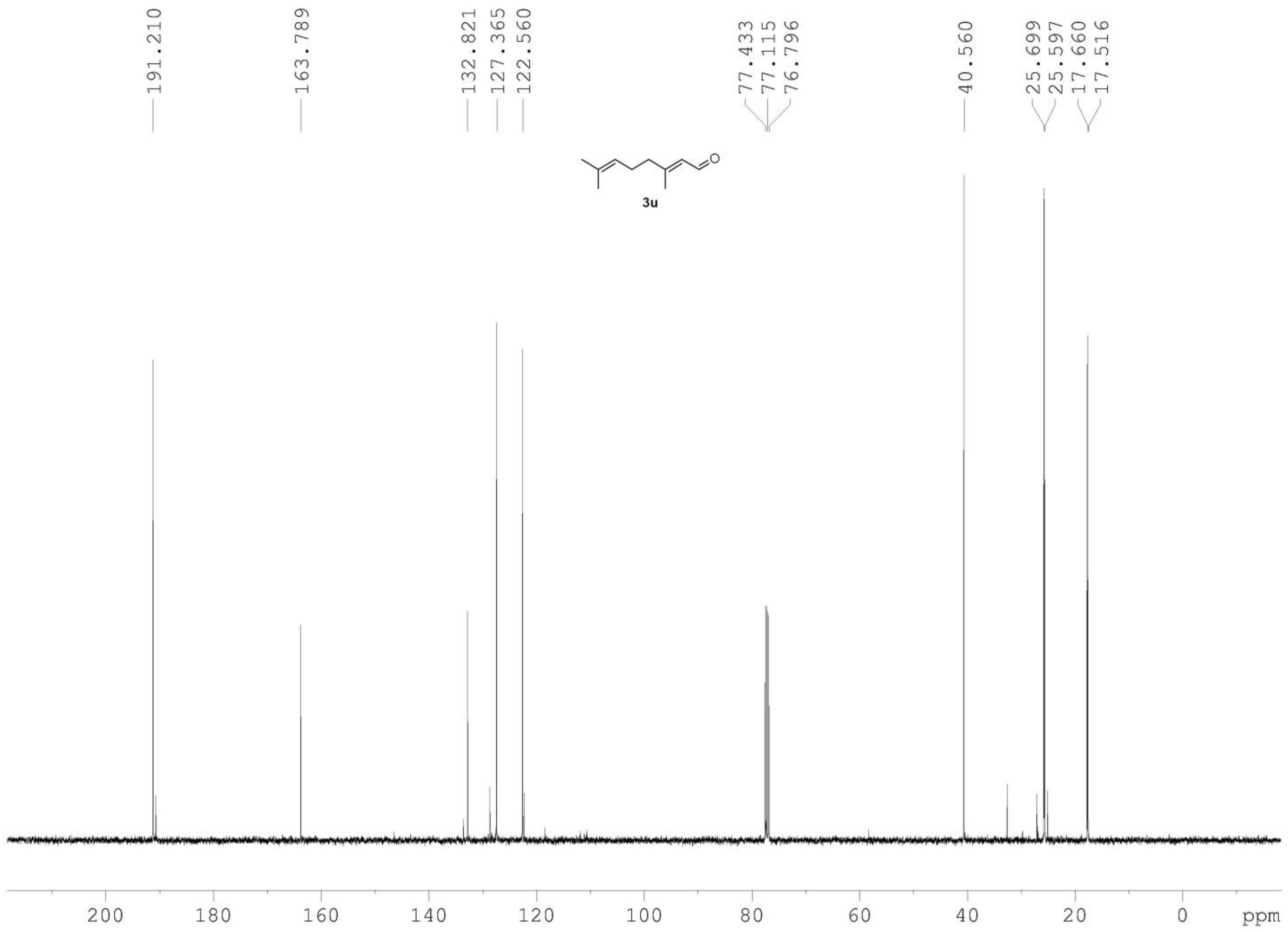

Figure S47. ^{1}H NMR spectrum (400 MHz, $CDCl_3$) of **3v**

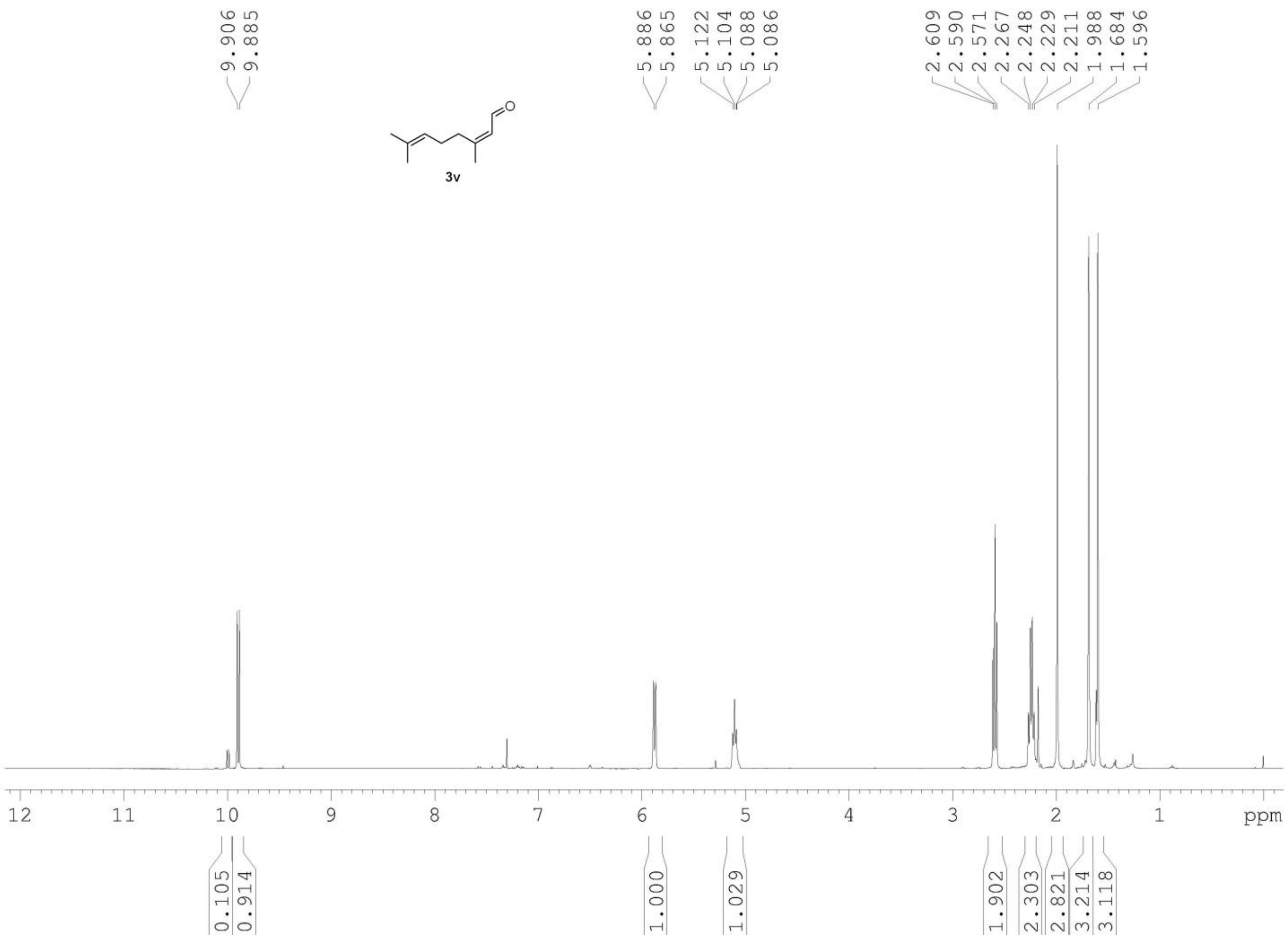

Figure S48. $^{13}C\{^{1}H\}$ NMR spectrum (100 MHz, $CDCl_3$) of **3v**

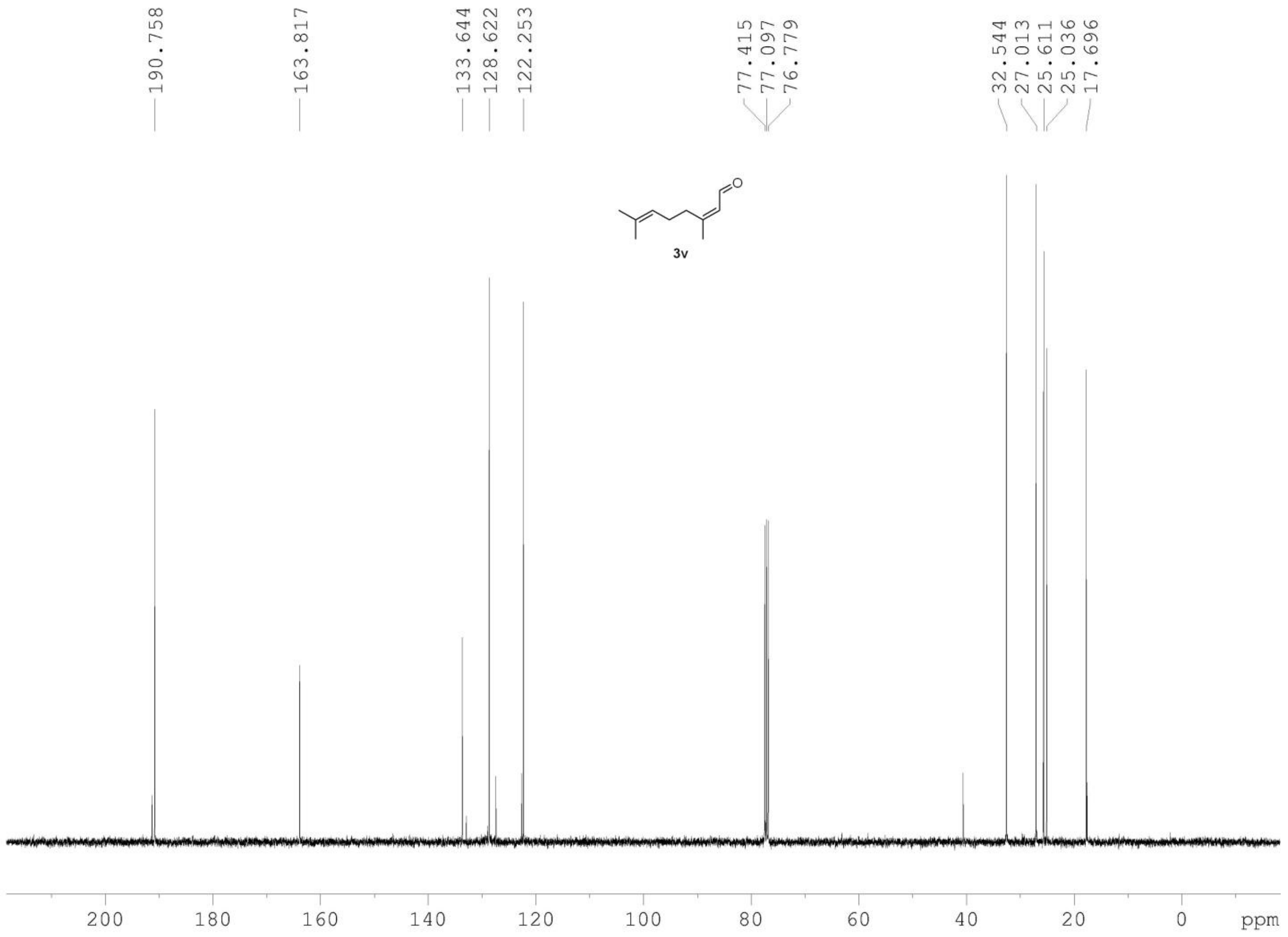

Figure S49. ^{1}H NMR spectrum (400 MHz, $CDCl_3$) of **3w**

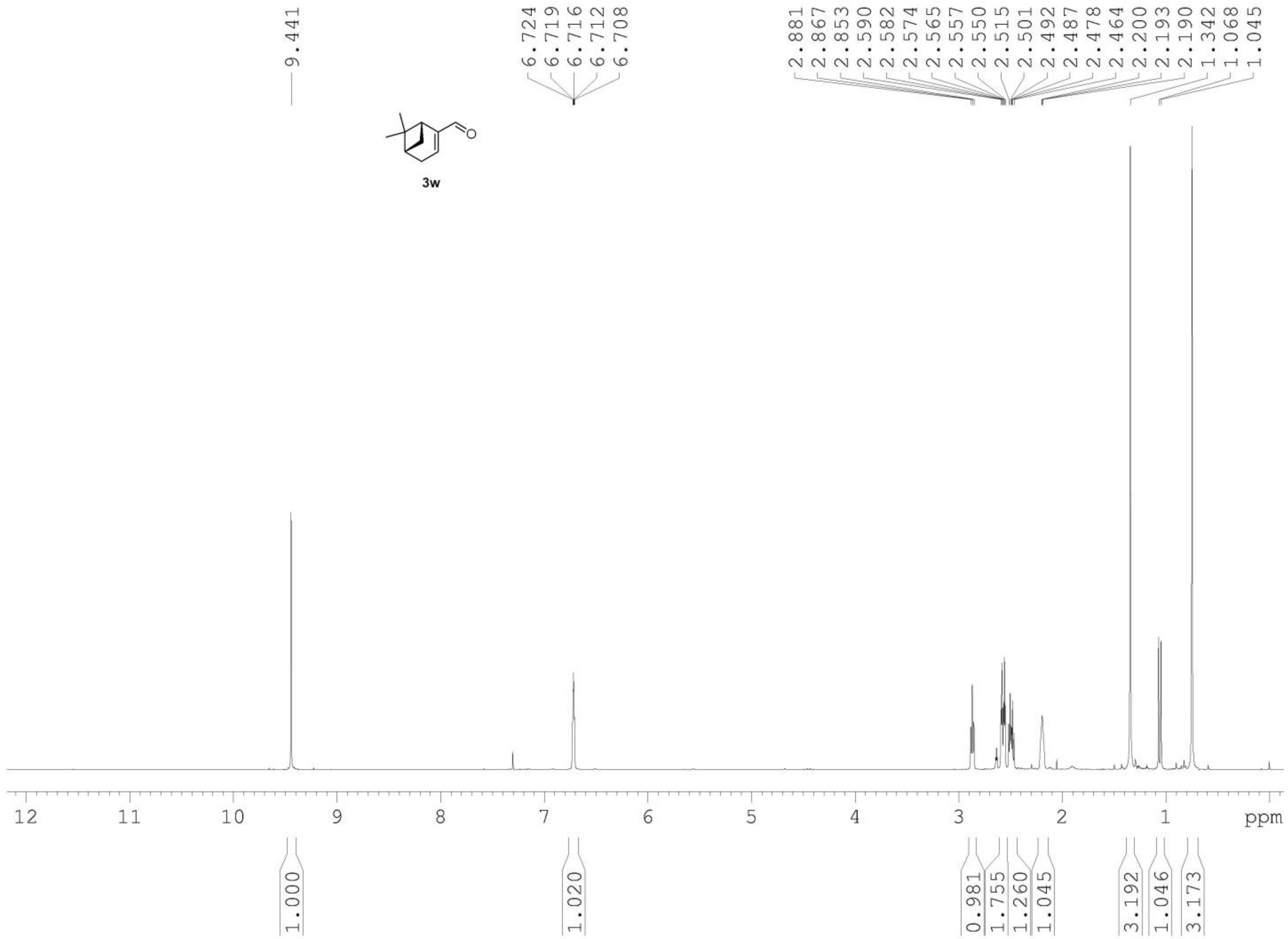

Figure S50. $^{13}C\{^{1}H\}$ NMR spectrum (100 MHz, $CDCl_3$) of **3w**

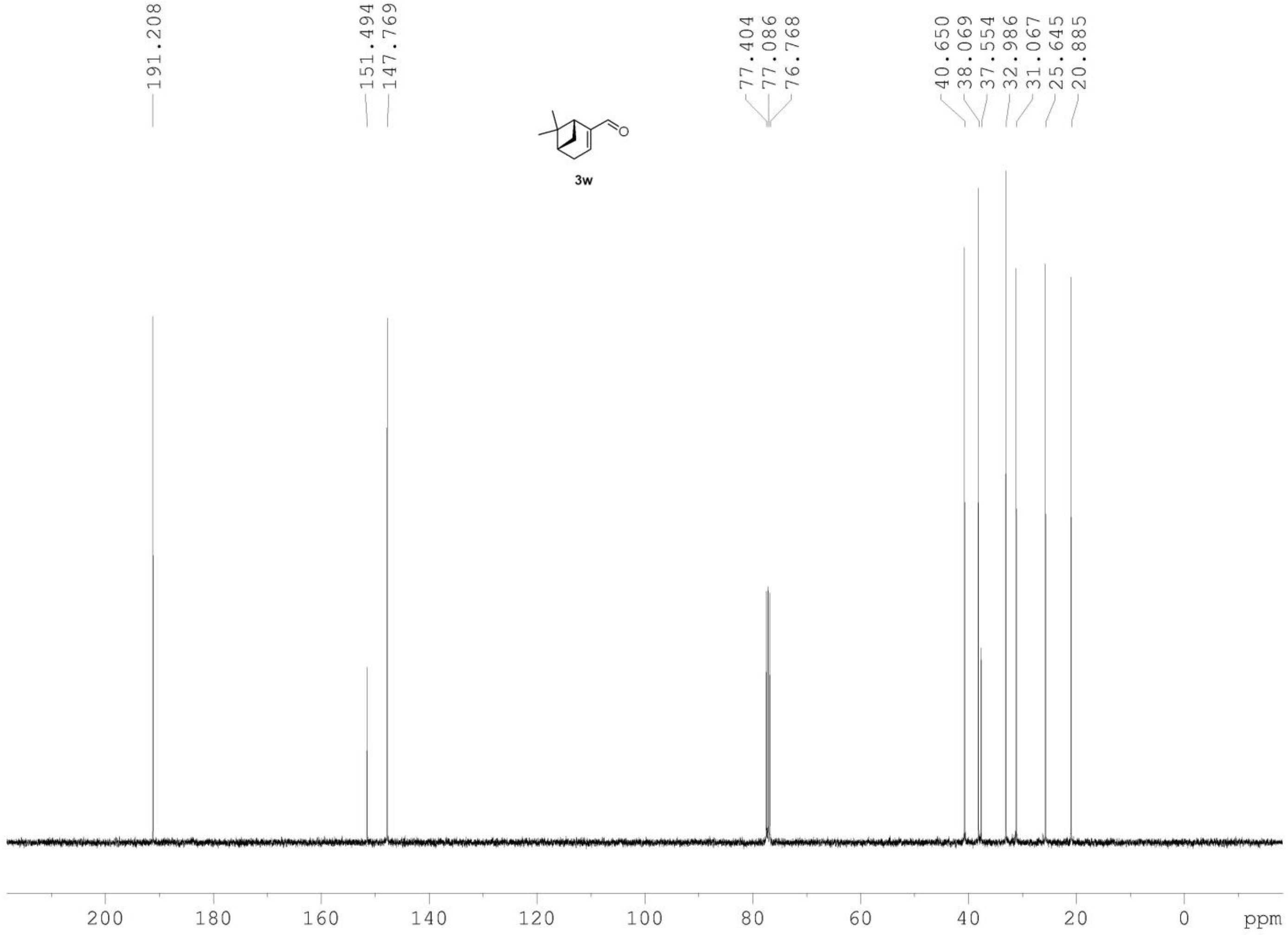

Figure S51. ^{1}H NMR spectrum (400 MHz, 70 °C, C_6D_6) of 2-heptanol oxidation catalyzed by **4**, t = 10 minutes (Figure 3). No peaks were observed between 0 ppm and –30 ppm.

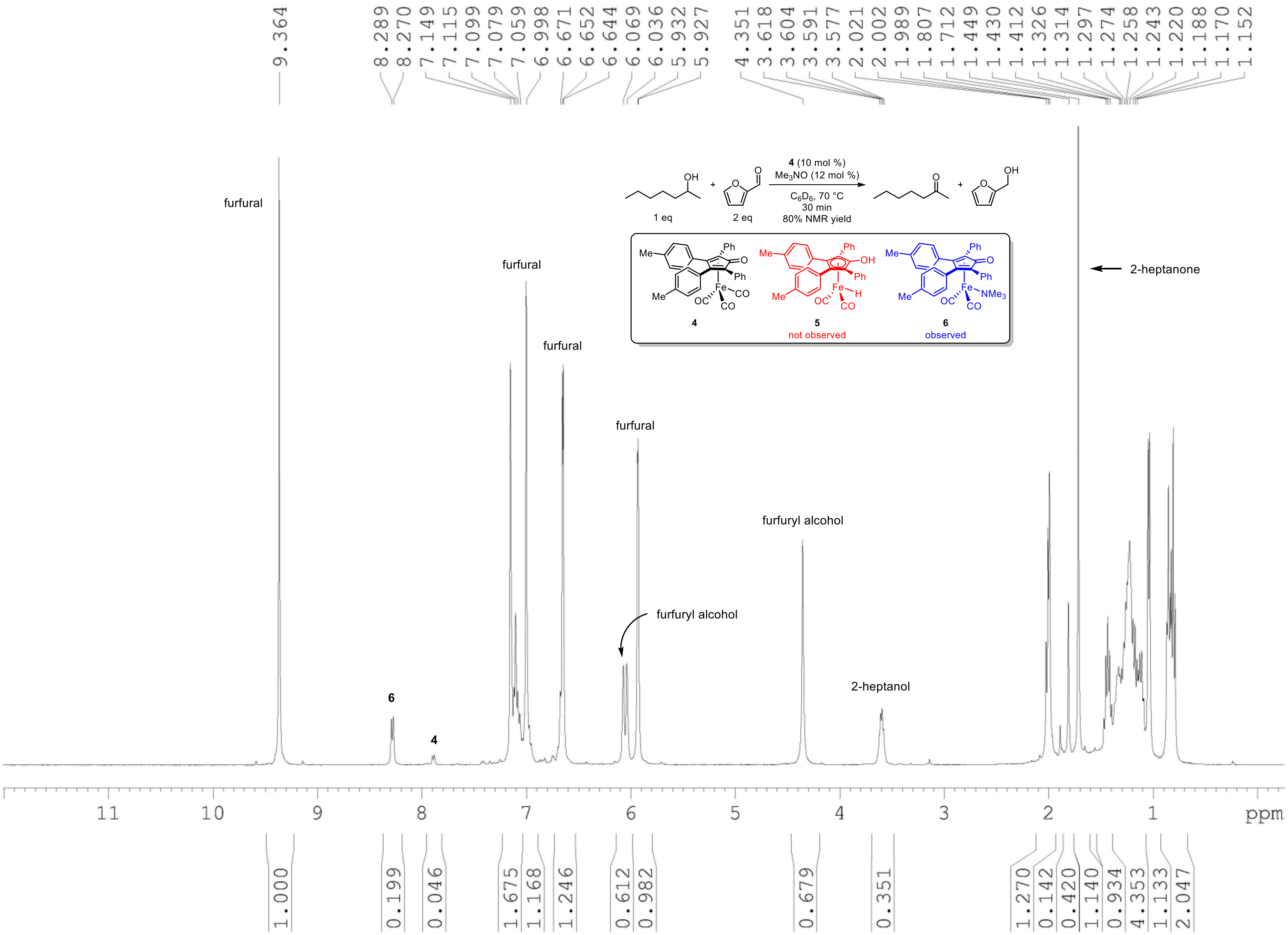

Figure S52. ^{1}H NMR spectrum (400 MHz, 70 °C, C_6D_6) of 2-heptanol oxidation catalyzed by **4**, t = 20 minutes (Figure 3). No peaks were observed between 0 ppm and –30 ppm.

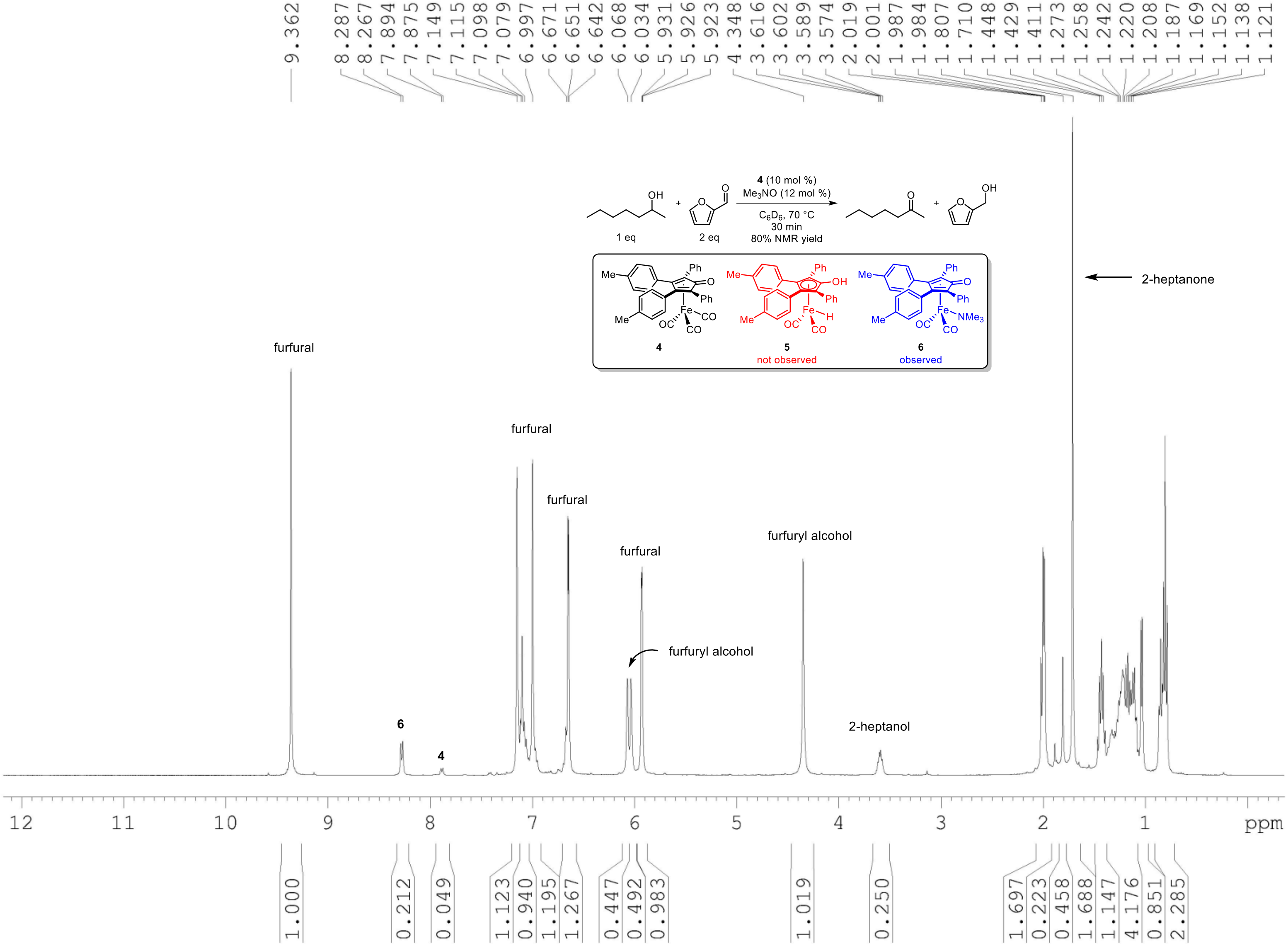

Figure S53. Example ^{1}H NMR spectrum (400 MHz, $CDCl_3$) of **16-Me** oxidation catalyzed by **11** using furfural (Table 9)

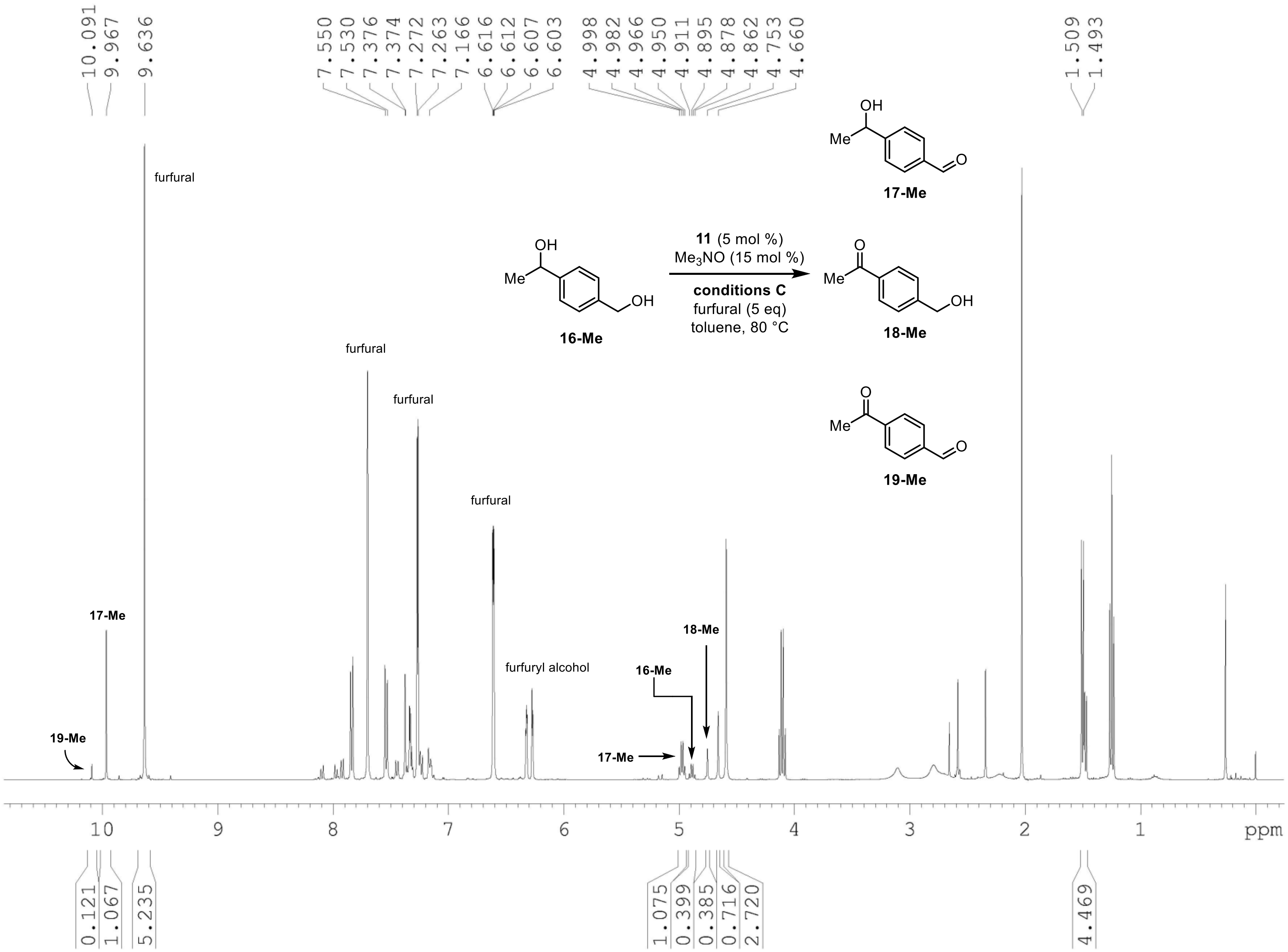

Figure S54. Example ^{1}H NMR spectrum (400 MHz, $CDCl_3$) of **16-Ph** oxidation catalyzed by **1** in acetone (Table 9)

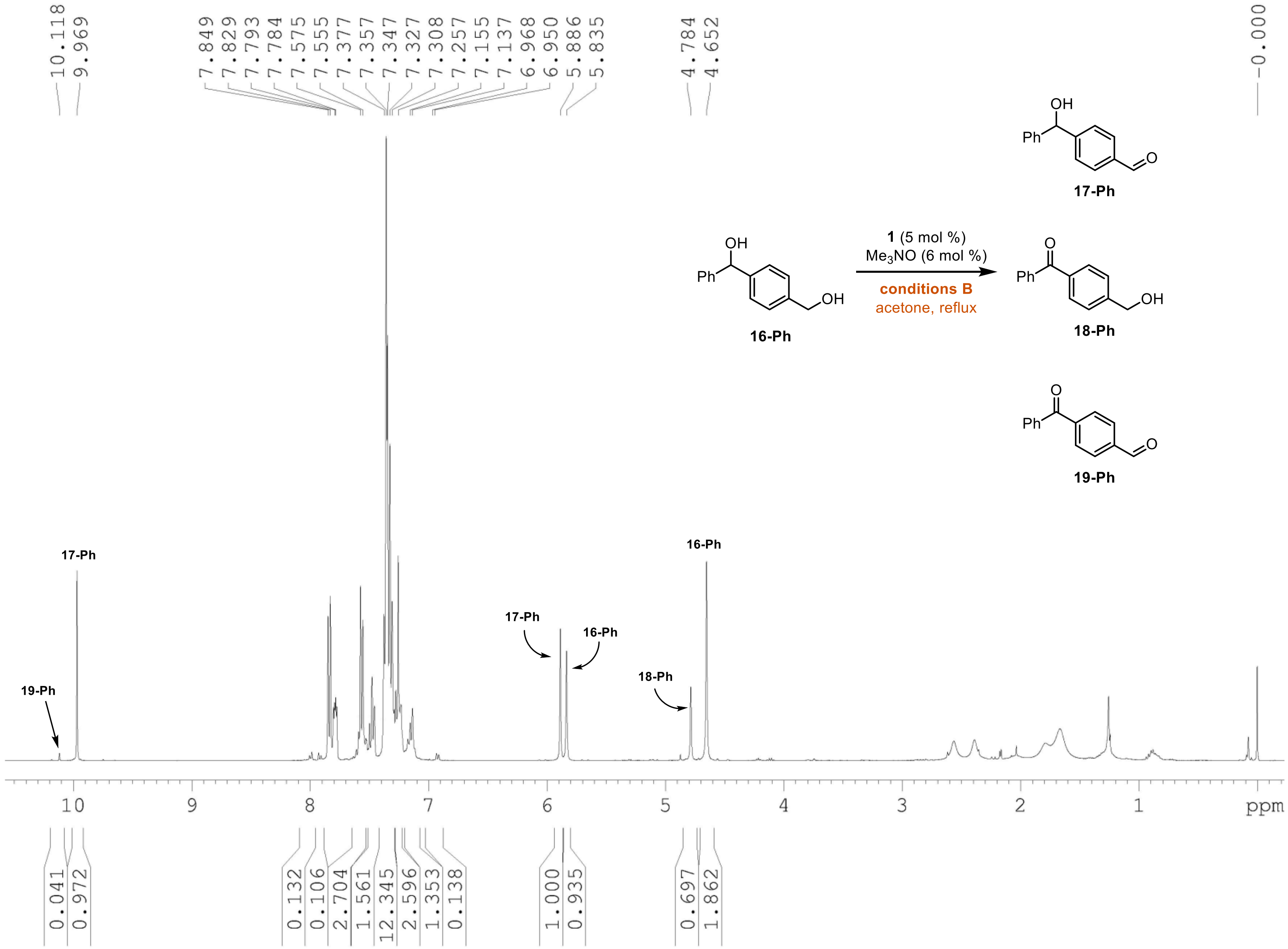

Supplement: Supplementary file 1 — jo4c02846_si_001.pdf [file jo4c02846_si_001.pdf]
